# Outcomes after TIPS in patients with cirrhosis and sarcopenia: A systematic review and meta-analysis

**DOI:** 10.1016/j.jhepr.2025.101699

**Published:** 2025-11-29

**Authors:** Maria de Brito Nunes, Maria Gabriela Delgado, Jaume Bosch, Annalisa Berzigotti

**Affiliations:** 1 Department of Visceral Surgery and Medicine, Inselspital, Bern University Hospital, University of Bern, Switzerland; 2 Department of Internal Medicine, Hospital of Fribourg, Switzerland; 3 Graduate School for Health Sciences (GHS), University of Bern, Bern, Switzerland

**Keywords:** Transjugular intrahepatic portosystemic shunt (TIPS), Cirrhosis, Portal hypertension, Sarcopenia, Hepatic encephalopathy, Mortality after TIPS

## Abstract

**Background & Aims:**

Transjugular intrahepatic portosystemic shunt (TIPS) is an established treatment for complications of portal hypertension (variceal bleeding and refractory ascites). Sarcopenia affects 40–70% of patients with cirrhosis. We evaluated the improvement in sarcopenia after TIPS, the proportion of patients developing overt hepatic encephalopathy (HE) after TIPS and its association with sarcopenia, and the association between sarcopenia and mortality after TIPS.

**Methods:**

We conducted a systematic review and meta-analysis according to the Preferred Reporting Items for Systematic Reviews and Meta-analyses (PRISMA) guidelines. Eligible studies included adults with cirrhosis and sarcopenia assessed by computed tomography (CT), using the skeletal muscle index (L3-SMI), or psoas muscle index (L3-PMI). We searched in eight databases for randomized trials, cohort studies, and case–control, cross-sectional, and case series studies.

**Results:**

Twenty studies were included. The pooled prevalence of sarcopenia in patients with cirrhosis undergoing TIPS was 55.4% (95% CI 41.7–68.2%). Among these, sarcopenia improved in 57% of patients (95% CI 48–65%) after TIPS, with a mean L3-SMI increase of 4.53 cm^2^/m^2^ (range: 2.4–6.9 cm^2^/m^2^). Sarcopenia was associated with higher odds of overt HE (pooled odds ration [OR] = 3.40, 95% CI 1.85–6.25, *p* <0.001) and a higher proportion of overt HE (43%, 95% CI 26–61%) than in patients without sarcopenia (12%, 95% CI 7–20%). The proportionate mortality after TIPS was 18.3% (95% CI 14.6–22.8%), across 6–33.6 months of follow-up. The association between sarcopenia and mortality was not significant (HR: 1.95, 95% CI 0.89–4.31, *p* = 0.078).

**Conclusions:**

In patients with cirrhosis and sarcopenia, TIPS is often followed by an improvement in sarcopenia. Sarcopenia is associated with higher odds of overt HE, whereas the effect of sarcopenia on mortality after TIPS remains uncertain.

**Impact and implications:**

Sarcopenia affects over half of patients with cirrhosis undergoing transjugular intrahepatic portosystemic shunt (TIPS) placement. This meta-analysis shows that TIPS is associated with sarcopenia improvement in >50% of patients, suggesting potential benefits beyond portal pressure reduction. Sarcopenia increases the risk of overt hepatic encephalopathy, whereas its effect on post-TIPS mortality remains inconclusive. These findings support routine assessment of sarcopenia to improve risk stratification and clinical decision-making. However, results should be interpreted cautiously because of study heterogeneity and the retrospective nature of the included data. Prospective studies are needed to confirm these findings and refine patient selection for TIPS.

## Highlights

• Sarcopenia is common in patients with cirrhosis undergoing TIPS (∼55% prevalence).
• TIPS improves sarcopenia in 57% of cases.
• Pre-existing sarcopenia is associated with a 3-fold increased risk of overt HE after TIPS (pooled OR 3.40, 95% CI 1.85–6.25, *p* <0.001).
• Overall mortality after TIPS is 18% (95% CI 14.6–22.8%).

## Graphical abstract

**Figure:**
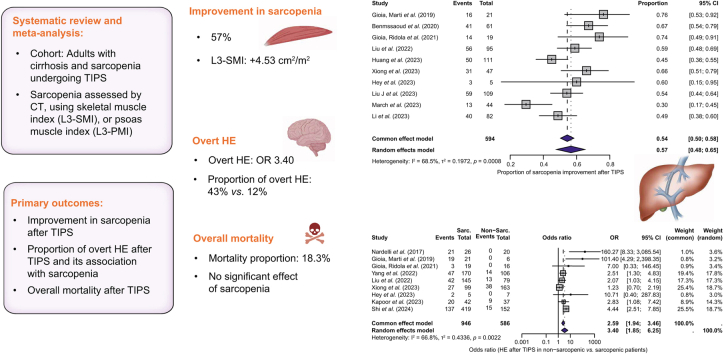

## Introduction

Transjugular intrahepatic portosystemic shunt (TIPS) is an established procedure for managing severe complications of portal hypertension, such as variceal bleeding and refractory ascites.[1], [2], [3], [4] Sarcopenia is a prevalent and debilitating condition in cirrhosis, with an estimated prevalence of 40–70%.[5], [6], [7] Sarcopenia is characterized by progressive loss of skeletal muscle mass and function, which is quantified using imaging. Computed tomography (CT) at the third lumbar vertebra (L3) is a well-validated method, correlating with whole-body muscle mass.^6^^,^^7^ This approach measures the cross-sectional area of major muscle groups (*e.g.* psoas, erector spinae, and abdominal muscles) at L3. This area is adjusted for patient height to obtain the L3-Skeletal Muscle Index (L3-SMI, cm^2^/m^2^), although cut-off values vary by sex, age, and study population. A simpler surrogate marker is the L3-Psoas Muscle Index (PMI), the psoas muscle area at L3 normalized for height (cm^2^/m^2^).^8^

Sarcopenia is associated with increased morbidity and mortality in patients with cirrhosis, before and after liver transplantation.^9^ A recent study identified sarcopenia and portal hypertension as key risk factors for decompensation, ascites, and mortality in patients with cirrhosis,^10^ while a meta-analysis reported a 2-fold higher mortality in patients with cirrhosis and sarcopenia.^11^ In patients with cirrhosis undergoing TIPS, sarcopenia has been associated with a higher risk of hepatic encephalopathy (HE) and increased mortality.^5^^,^^12^^,^^13^ However, emerging data suggest that TIPS improves sarcopenia, with contrasting effects on overt HE and mortality.^14^^,^^15^

Thus, we performed a systematic review and meta-analysis to evaluate: (1) improvement in sarcopenia after TIPS; (2) the proportion of patients developing overt HE after TIPS and its association with sarcopenia; and (3) the association between sarcopenia and mortality after TIPS.

## Patients and methods

### Protocol registration

This systematic review is reported according to the Preferred Reporting Items for Systematic Reviews and Meta-analyses (PRISMA) recommendations.^16^ The PROSPERO registered protocol number is CRD42025646782.

### Selection criteria and search strategy

We included peer-reviewed randomized controlled trials, cohort studies, case–control, cross-sectional studies and case series studies with peer-review. Eligible studies were those that had enrolled patients with cirrhosis who underwent TIPS and had sarcopenia assessed by CT using L3-SMI or L3-PMI.

Primary outcomes in patients with cirrhosis included improvement in sarcopenia after TIPS, the proportion developing overt HE after TIPS and its association with sarcopenia, and the association between sarcopenia and mortality after TIPS.

Sarcopenia was assessed by CT in accordance with the EASL Nutrition Guideline^17^ and AASLD Guidance on Malnutrition and Sarcopenia.^18^ We excluded studies using magnetic resonance imaging (MRI) because of limited standardization, a lack of validated reference values in cirrhosis, and poorer comparability between studies. The cross-sectional area of skeletal muscle at the L3 level (cm^2^) was measured and normalized to patient height to calculate either L3-SMI or L3-PMI.^17^

Improvement in sarcopenia was defined as the transition from sarcopenia to non-sarcopenia, or an increase of at least 10% in L3-SMI or L3-PMI. Estimates were considered statistically significant if the 95% CI excluded the null value. Clinical significance based on functional measures (muscle strength/performance) was not analyzable because these outcomes were seldom reported. Overt HE was defined as grade II or higher according to the West Haven criteria.^19^

We searched electronic databases from inception to January 31, 2025, with no language restrictions. Text words related to the research question and medical subject headings (MeSH) were used to search in Google Scholar, Medline (OVID interface), Embase (via OVIDSP), PubMed, Cochrane Library, Cochrane Central Register of Controlled Trials (CENTRAL), clinicaltrials.gov, EU Clinical Trials, and citations therein. Full search strategies are provided in Table S1.

### Data extraction and quality assessment

Two researchers (MBN and MGD) independently screened titles, abstracts, and full texts for eligibility. Disagreements were resolved by discussion with two additional reviewers (AB and JB). Studies excluded in the full-text analysis were recorded, accompanied by a justification. For studies with multiple publications, we extracted data from the article with the most complete dataset. Using a standardized form, two authors (MBN and MGD) extracted the following data: author/publication year; study design and main outcomes of the study; sample size; duration of follow-up; population characteristics; pre and post-TIPS nutritional supplementation; indication of TIPS; cases of sarcopenia defined by L3-SMI or L3-PMI before TIPS; cases of sarcopenia defined by L3-SMI or L3-PMI after TIPS; cases of overt HE after TIPS; mortality after TIPS; and causes of death.

The risk of bias of each study was assessed by MBN and MGD using the Newcastle-Ottawa Scale (NOS) for cohort studies (Table S2). Studies were classified as high quality (*i.e.* low risk of bias) if ≥7 points, moderate quality if 5 or 6 points, and low quality if ≤4 points (*i.e.* high risk of bias). Twelve studies were classified as high quality,^15^^,^[20], [21], [22], [23], [24], [25], [26], [27], [28], [29], [30] seven studies were as of moderate quality,^14^^,^^29^^,^[31], [32], [33], [34], [35] and one study was of low quality.^36^

### Data analysis

We calculated the odds ratio (OR) with 95% CI to assess the association between sarcopenia and overt HE after TIPS. If studies did not directly report ORs, these were estimated from available event counts and sample sizes. For mortality, we extracted hazard ratios (HRs) with 95% CIs from the original studies and the total number of deaths during the follow-up.

We summarized improvement in sarcopenia, proportion of overt HE, and mortality proportion after TIPS as pooled proportions with 95% CIs. Changes in L3-SMI (cm^2^/m^2^) were visualized using bar plots showing mean L3-SMI improvements across studies.

Given the expected clinical and methodological heterogeneity, we used random-effects models for all primary analyses and common-effect (fixed-effect) models as sensitivity analyses. We quantified heterogeneity with I^2^ and its *p* value (Cochran’s Q). We considered heterogeneity to be substantial when I^2^ ≥50% or *p* ≤0.10. If at least 10 studies were available, we explored heterogeneity via subgroup analyses (categorical modifiers) and meta-regression (continuous modifiers). Candidate effect modifiers included age, L3-SMI/L3-PMI cut-offs, TIPS indication, portal pressure gradient, liver disease severity (model of end-stage liver disease score [MELD] score), and follow-up duration after TIPS.

Publication bias was assessed using funnel plots, if ≥10 studies were available. We inspected funnel plot asymmetry visually and, if appropriate, applied Egger’s regression test. The quality of evidence was evaluated using the GRADE framework.^37^ Details are provided in Table S3. Statistics analyses were performed using R (version 4.2.3, R Foundation for Statistical Computing, Vienna, Austria).

## Results

**Fig. 1:**
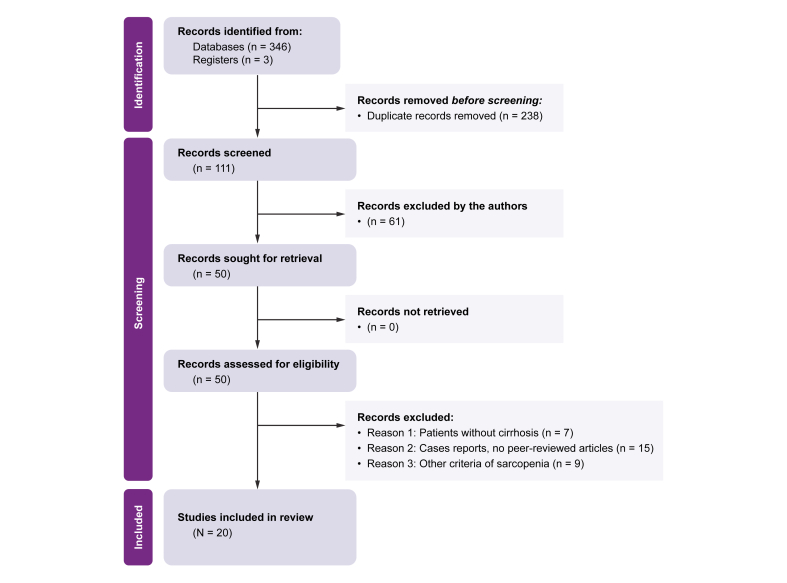
PRISMA flow diagram of the systematic literature search process. PRISMA, Preferred Reporting Items for Systematic Reviews and Meta-analyses.

We identified 346 records after database searches, with three additional records from trial registries. After removing 238 duplicated records, 111 records were screened. We analyzed 50 full-text articles, of which 20 met the inclusion criteria (Fig. 1).

After excluding one study with high risk of bias,^36^ 19 studies were included in the meta-analysis: two were prospective observational studies^20^^,^^28^ and 17 were retrospective observational studies.^14^^,^[21], [22], [23], [24], [25], [26], [27]^,^[29], [30], [31], [32], [33], [34], [35]^,^^38^^,^^39^ The indication for TIPS was refractory ascites and variceal bleeding in 17 studies,^14^^,^^21^^,^^22^^,^^24^^,^^25^^,^[27], [28], [29], [30]^,^[32], [33], [34], [35]^,^^39^ variceal bleeding alone in two studies,^23^^,^^39^ and refractory ascites alone in one study.^26^ The included studies applied different cut-offs values to assess sarcopenia, based on L3-SMI or L3-PMI measured by CT (Table 1).

**Table 1:** Cut-off values for defining sarcopenia used by included studies.

Sarcopenia definition	Female (cm^2^/m^2^)	Male (cm^2^/m^2^)	Studies
EASL/AASLD (L3-SMI)	<39	<50	11 studies^14^^,^^21^^,^^22^^,^[25], [26], [27]^,^^30^^,^^32^^,^^35^
Chinese specific (L3-SMI)	<32.50	<44.77	2 studies^15^^,^^31^
JSH (L3-SMI)	<38	<42	1 study^33^
Alternative cut-offs (L3-SMI)	<39.5	<52	1 study^34^
	<38.5	<50	1 study^20^
	<38.5	<52.4	1 study^28^
	<41	<53	1 study^24^
L3-PMI	<3.2	<4.4	1 study^29^

JSH, Japanese Society of Hepatology; L3-PMI, psoas muscle index at the third lumbar vertebra; L3-SMI, skeletal muscle index at the third lumbar vertebra.

Information on concomitant nutritional supplementation or physical rehabilitation during follow-up was not reported. Baseline characteristics of the studies are summarized in Table S4.

### Improvement in sarcopenia after TIPS

Ten studies involving a total of 1,008 patients (596 with sarcopenia criteria) evaluated improvement in sarcopenia after TIPS, using L3-SMI or L3-PMI measurements. The baseline prevalence of sarcopenia in patients with cirrhosis undergoing TIPS, assessed in eight studies,^20^^,^^23^^,^[25], [26], [27]^,^[33], [34], [35] was 55.4% (95% CI: 41.7–68.2%). Two studies^14^^,^^21^ including only patients with sarcopenia were excluded from this prevalence estimate. The corresponding forest plot is shown in Fig. S1.

**Fig. 2:**
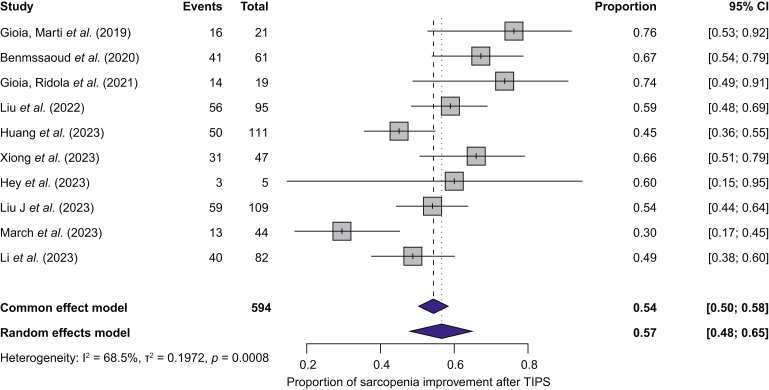
Proportion of patients with improvement of sarcopenia and 95% CI in patients with sarcopenia undergoing TIPS. TIPS, transjugular intrahepatic portosystemic shunt. Between-study heterogeneity was assessed with I² and Cochran’s Q test (*p* value).

**Fig. 3:**
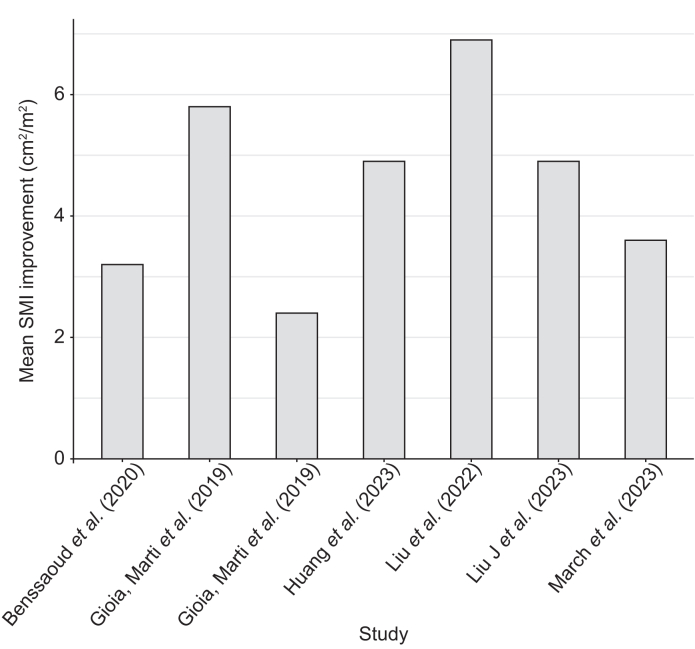
Mean L3-SMI improvement by study. The pooled mean change was estimated separately using a random-effects meta-analysis. L3-SMI, skeletal muscle index at the third lumbar vertebra.

The pooled proportion with improvement in sarcopenia after TIPS was 57% (95% CI 48–65) using a random-effects model. A common-effect (fixed-effect) analysis provided similar results (Fig. 2). Heterogeneity was substantial (I^2^ = 68.5%, *p* <0.0008). Funnel plot asymmetry was suggested visually (Fig. S2) but not confirmed by Egger’s test (z = 1.23, *p* = 0.22). Subgroup analysis by follow-up (≤6 months, >6–12 months, and >12 months) showed no significant differences (*p* = 0.86; Fig. S3). The median L3-SMI improvement after TIPS in patients with sarcopenia was 4.53 cm^2^/m^2^ (range: 2.4–6.9 cm^2^/m^2^) across seven studies (Fig. 3).

Other nonstudied factors, such as having concomitant nutritional supplementation, physical rehabilitation, severity of liver disease, or L3-SMI or L3-PMI cut-offs, could influence this outcome. Subgroup analysis by liver disease severity (MELD score) could not be performed, because separate MELD scores for patients with and without sarcopenia were unavailable. Subgroup analysis by sarcopenia cut-offs was not performed because of limited statistical power, given that some cut-offs appeared in only one or two studies.

### Overt hepatic encephalopathy after TIPS

Eleven studies,^20^^,^^22^^,^^23^^,^[25], [26], [27], [28]^,^[30], [31], [32]^,^^35^ including 1,839 patients (1,080 with sarcopenia), reported the proportion of overt HE after TIPS. The pooled proportion of overt HE after TIPS was 31.5% (95% CI 23.5–40.6%), as estimated by the random-effects model. A common-effect (fixed-effect) analysis gave similar results (30.8%, 95% CI 28.7–32.9), with high heterogeneity (I^2^ = 85.7%, *p* <0.01; Fig. S4).

**Fig. 4:**
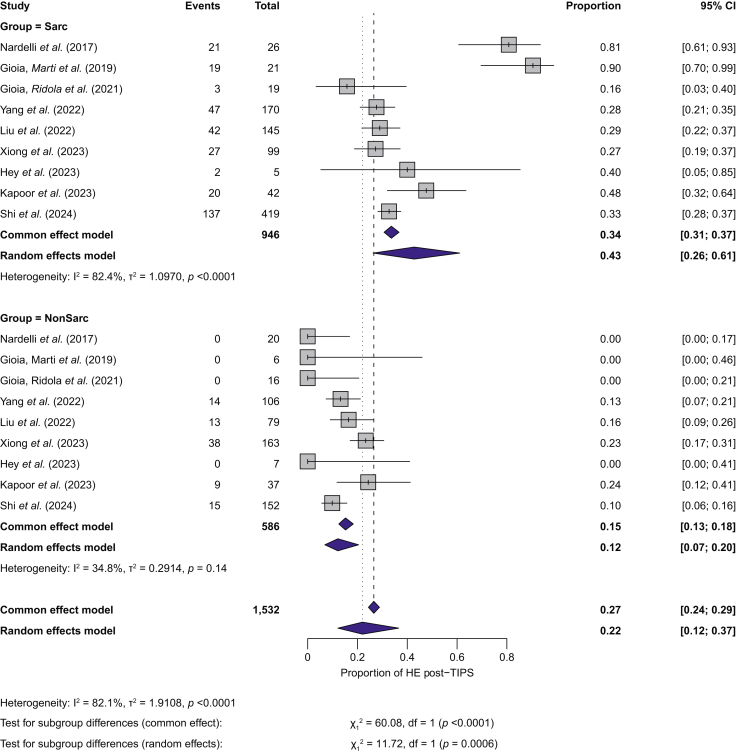
Subgroup analysis comparing the proportion of HE after TIPS between patients with sarcopenia (upper panel) and without sarcopenia (lower panel). Proportions with 95% CIs are shown for each study. Between-study heterogeneity was assessed with I² and Cochran’s Q test (*p* value). HE, Hepatic encephalopathy; TIPS, transjugular intrahepatic portosystemic shunt.

The proportion of overt HE after TIPS was higher in patients with sarcopenia (43%, 95% CI 26–61%) than in patients without sarcopenia (12%, 95% CI 7–20%). The random-effects model confirmed a statistically significative difference (χ^2^ = 11.72, degrees of freedom = 1, *p* <0.01). Substantial heterogeneity was observed in the group with sarcopenia (I^2^ = 82%, *p* <0.01), whereas it was low in the group without sarcopenia (I^2^ = 18.4%, *p* = 0.14), suggesting consistency among studies (Fig. 4).

Visual inspection suggested funnel plot asymmetry (Fig. S5), but Egger’s test did not indicate small-study effects (*p* = 0.51). Observed proportions of 0.00 in several small studies^20^^,^^25^^,^^27^^,^^28^ reflected no overt HE events in one of the subgroups.

**Fig. 5:**
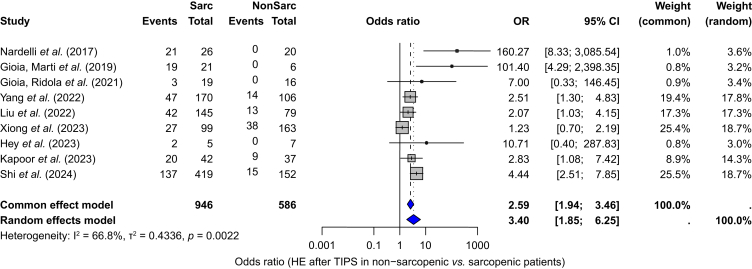
ORs for the development of overt HE after TIPS in patients with and without sarcopenia. ORs with 95% CIs are shown for each study. Between-study heterogeneity was assessed with I² and Cochran’s Q test (*p* value). HE, Hepatic encephalopathy; OR, odds ratio; TIPS, transjugular intrahepatic portosystemic shunt.

Sarcopenia was associated with higher odds of developing overt HE after TIPS (pooled OR, 3.40; 95% CI 1.85–6.25; *p* <0.001; Fig. 5). A continuity correction of 0.5 was applied to handle zero HE events in the group without sarcopenia, especially seen in studies with low samples sizes. Three individual studies reported associations consistent with this finding. Kapoor *et al.*^22^ reported higher odds of overt HE after TIPS in patients with sarcopenia (OR = 2.8, 95% CI 1.08–7.4, *p* = 0.02). On univariate logistic regression model, low L3-SMI was a risk factor for overt HE after TIPS (OR = 0.94, 95% CI 0.89–0.99, *p* = 0.03). Nardelli *et al.*^28^ reported an increased risk of overt HE after TIPS associated with sarcopenia on multivariate competing risk regression analysis (subdistribution HR = 31.3, 95% CI 4.5–218.07, *p* <0.001). Benmassaoud *et al.*^26^ found an HR of 0.95 (95% CI 0.91–0.99, *p* = 0.01) on multivariate Cox regression analysis, indicating that each incremental increase (per unit) in L3-SMI reduced the risk of developing overt HE, and higher L3-SMI was protective against HE. Only Wang *et al.*^31^ reported a nonsignificant association of sarcopenia measured by L3-SMI and overt HE after TIPS in either male patients (OR = 0.94, 95% CI 0.87–1.02, *p* = 0.142) or female patients (OR = 0.95, 95% CI 0.87–1.04, *p* = 0.272).

A subgroup analysis (Fig. S6) comparing studies reporting previous HE (n = 7) *vs.* those not reporting it (n = 4) found no differences in the proportion of overt HE after TIPS (incidence 28.7% *vs.* 31.7%). Heterogeneity remained substantial (I^2^ = 84.5%). The test for subgroup differences was nonsignificant under either the common-effect (*p* = 0.19) or random-effects model (*p* = 0.94). In meta-regression (Fig. S7), follow-up time was not associated with the proportion of overt HE after TIPS (*p* = 0.454) and did not explain heterogeneity (R^2^ = 0.00%). Given limited available data, we could not assess whether patients with sarcopenia had a higher prevalence of overt HE before TIPS compared with patients without sarcopenia. Subgroup analysis based on liver disease severity, L3-SMI/L3-PMI cut-offs, age, or portal pressure gradient were not performed, for the same reasons.

### Mortality after TIPS

**Fig. 6:**
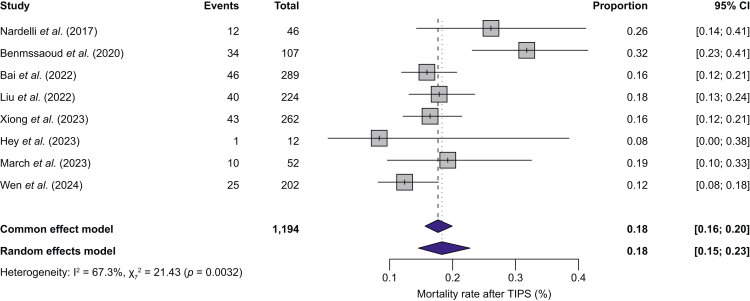
Overall proportionate mortality after TIPS in patients with cirrhosis. Proportions with 95% CIs are shown for each study. Between-study heterogeneity was assessed with I² and Cochran’s Q test (p value). Mortality was reported at the last available follow-up, which ranged from 6 to 33.6 months across studies. TIPS, transjugular intrahepatic portosystemic shunt.

Eight studies^20^^,^^23^^,^^24^^,^^26^^,^^28^^,^^34^^,^^35^^,^^39^ reported overall mortality after TIPS, including 1,194 patients and 211 deaths. The overall mortality proportion was 18.31% (95% CI 14.57–22.77%) according to a random-effects model. Comparable results were obtained considering a common-effect (fixed-effect) model (Fig. 6). The follow-up duration varied across studies, ranging from 6 to 33.6 months (median: 16.5 months).

**Fig. 7:**
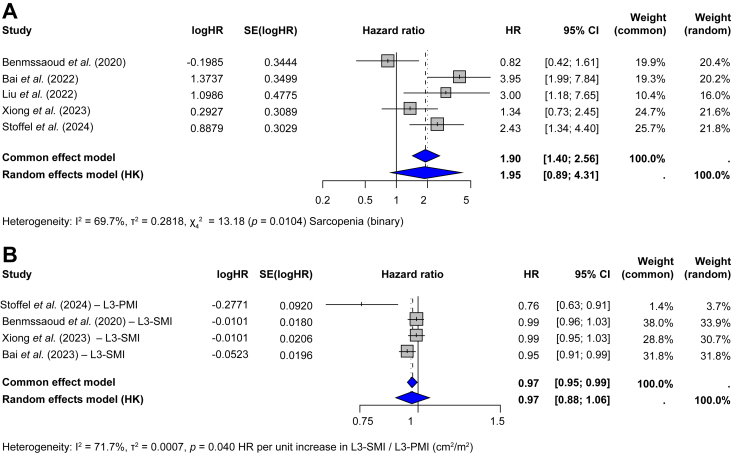
Association between sarcopenia and mortality after TIPS. (A) HRs for mortality comparing patients with and without sarcopenia (binary variable), regardless of adjustment status (univariate and multivariate Cox regression model). (B) HRs per unit increase in L3-SMI/L3-PMI (continuous variable), calculated by univariate cox regression model. HRs with 95% CIs are shown for each study. Between-study heterogeneity was assessed with I² and Cochran’s Q test (*p* value). HR, hazard ratio; L3-PMI, psoas muscle index at the third lumbar vertebra; L3-SMI, skeletal muscle index at the third lumbar vertebra; TIPS, transjugular intrahepatic portosystemic shunt.

The pooled HR from five studies comparing HRs for mortality after TIPS in patients with and without sarcopenia was 1.95 (95% CI 0.89–4.31, *p* = 0.0783), not reaching statistical significance, under a random-effects model (Fig. 7A). In the subset of studies reporting adjusted HRs, the pooled adjusted HR was 2.86 (95% CI 1.51–5.40, *p* = 0.5131; Fig. S9). A meta-analysis of unadjusted HRs for the same give studies also showed no significant association (HR = 2.04, 95% CI 0.84–4.83, *p* = 0.0828; Fig. S8). In a meta-regression pooling adjusted and unadjusted HRs, adjustment status did not influence effect size (HR = 1.12; 95% CI 0.69–1.84; *p* = 0.64; Fig. S10).

In a meta-analysis of four studies reporting HRs per unit increase in skeletal muscle indices (L3-SMI or L3-PMI), the pooled HR was 0.97 (95% CI 0.88–1.06, *p* = 0.34) under a random-effects model, indicating no statistically significant association (Fig. 7B). However, the common-effect (fixed-effect) model showed a significant association (HR = 0.97, 95% CI 0.95–0.99, *p* = 0.013), reporting a 3% reduction in mortality risk per cm^2^/m^2^ increase in muscle mass. Heterogeneity was substantial (I^2^ = 71.7%, *p* = 0.014). All HRs in this analysis were derived from univariate Cox regression models. Given that only five studies reported HRs for mortality after TIPS, subgroup analyses or meta-regression by L3-SMI/L3-PMI cut-offs, liver disease severity (MELD), or follow-up duration after TIPS were not feasible.

## Discussion

This study provides a comprehensive summary of outcomes in patients with cirrhosis and sarcopenia undergoing TIPS. The pooled prevalence of sarcopenia in patients undergoing TIPS was 55.4% (95% CI 41.7–68.2%). Among 596 patients with sarcopenia, 57% (95% CI 48–65%) demonstrated an improvement in sarcopenia after TIPS, with a mean L3-SMI increase of 4.53 cm^2^/m^2^. The pooled proportion of overt HE after TIPS was 31.5% (95% CI 23.5–40.6%). This proportion was higher in patients with sarcopenia (43%) than in those without sarcopenia (12%) (*p* <0.01). Sarcopenia was associated with higher odds of overt HE after TIPS (pooled OR, 3.40, *p* <0.001). The overall mortality proportion after TIPS was 18.3% (95% CI 14.6–22.8%). There was no significant association between sarcopenia and mortality (HR = 1.64, 95% CI 0.91–2.98, *p* = 0.087).

Half of patients with baseline sarcopenia experienced improvement in sarcopenia after TIPS. This aligns with the systematic review by Gădža *et al.*,^40^ which reported improvements in ascites-free body weight, BMI, or body mass area in 10 studies evaluating nutritional outcomes after TIPS. Artru *et al.*.^41^ also observed changes in body composition after TIPS using transversal psoas thickness/height (TPMT/height), a surrogate marker of sarcopenia. Their findings are consistent with ours, but differences in methodology precluded inclusion in our pooled analysis. TIPS reduces portal hypertension, resulting in decreased intestinal congestion and likely improved absorption of nutrients. This is supported by the observation that TIPS can decrease bacterial translocation and associated systemic inflammation.^42^^,^^43^ Recent studies showed that TIPS placement leads to a significant reduction in proinflammatory cytokines, particularly IL-6, which has a central role in the inflammatory response in cirrhosis.^42^ By contrast, elevated levels of IL-8 predicted worse clinical outcomes after TIPS.^44^ The degree of improvement in sarcopenia might still depend on baseline liver function, nutritional status and supplementation, physical activity, and length of follow-up after TIPS, factors that were not reported in the included studies.

The pooled OR for overt HE after TIPS in patients with sarcopenia was 3.40 (*p* <0.001), indicating ∼3-fold higher odds of overt HE after TIPS. Our findings align with those of Ahmed *et al.*,^45^ who reported a pooled risk ratio of 1.68 (95% CI 1.48–1.91; *p* <0.004), identifying sarcopenia as a risk factor of HE after TIPS. This study included heterogeneous definitions of sarcopenia, whereas our analysis focused on L3-SMI or L3-PMI measured by CT. The stronger association observed in our study could be attributed to this stricter imaging-based classification and the higher methodological homogeneity in how sarcopenia was measured. These findings support preprocedural assessment of sarcopenia as a key element in HE risk stratification. The pathophysiological mechanisms underlying this association are well described. First, sarcopenia reduces ammonia detoxification capacity, because skeletal muscle is an extrahepatic site for ammonia metabolism and conversion to glutamine.^46^ The reduction in extrahepatic ammonia metabolism contributes to the accumulation of neurotoxins after TIPS placement, increasing the risk of HE. Second, hyperammonemia accelerates muscle wasting by inducing mitochondrial dysfunction, cellular stress, and transcriptional upregulation of myostatin.^47^^,^^48^ Hyperammonemia is an important negative regulator of protein synthesis and cell proliferation and differentiation.^46^^,^^48^

In our meta-analysis, only three studies^25^^,^^27^^,^^30^ specified that patients did not receive HE prophylaxis before TIPS, even though HE prophylaxis can reduce overt HE after TIPS. A double-blind, placebo-controlled, randomized, multicenter trial showed that rifaximin, started 2 weeks before TIPS placement and continued for at least 6 months, reduced the incidence of overt HE after TIPS by ∼50%.^49^ A recent meta-analysis comparing the effectiveness of rifaximin, lactulose, lactitol, L-ornithine-L-aspartate (LOLA), albumin, and combinations reported that rifaximin plus lactulose was associated with fewer HE events after TIPS.^50^ EASL Clinical Practice Guidelines on TIPS^51^ recommend using lactulose to treat and prevent the recurrence of HE, and rifaximin can be added if lactulose is ineffective or not tolerated. The BAVENO VII consensus^1^ suggests considering rifaximin for preventing HE in patients with a history of HE undergoing elective TIPS.

A previous meta-analysis by Ahmed *et al.*^45^ reported an association (relative risk: 1.70, 95% CI 1.13–1.54, *p* <0.001) between sarcopenia and mortality after TIPS, but our results do not support those findings. Another meta-analysis by Dajti *et al.*,^11^ focusing on patients with cirrhosis (regardless of whether they underwent TIPS), showed that sarcopenia defined by EASL/AASLD CT-based criteria is an independent predictor of mortality. This discrepancy could result from the inclusion in our meta-analysis of two studies, by Benmassaoud *et al.*^26^ and Xiong *et al.*,^15^ reporting no association between sarcopenia and mortality after TIPS. In addition, the improvement in sarcopenia observed in 57% of patients after TIPS in our study could attenuate the adverse prognostic effects of sarcopenia.

The main limitation of our study is the substantial heterogeneity observed across analyses. Although random-effects models were used, our ability to explore the sources of heterogeneity was limited. Most studies did not report information separately for patients with and without sarcopenia, which restricts subgroup analyses. Key effect modifiers, such as concomitant nutritional supplementation, physical rehabilitation, prophylactic use of pharmacological treatment to prevent HE (rifaximin or lactulose), or prevalence of HE stratified by sarcopenia were not systematically reported, even though these factors can influence the outcomes after TIPS. Even with a uniform imaging approach (L3-SMI/L3-PMI measured by CT), heterogeneity persisted, in part, because of different cut-off values for sarcopenia that reflect ethnic differences in body composition. An individual patient data meta-analysis would allow harmonized sarcopenia definitions, adjustment for key confounders, and subgroup analyses by MELD, age, and portal pressure gradient. This was not feasible because of the lack of access to individual patient data. The use of CT imaging enhances internal consistency, but it can limit extrapolation to settings that estimate sarcopenia with other methods (*e.g.* bioelectrical impedance analysis, dual-energy X-ray absorptiometry, or MRI).

This study has important strengths. All included studies assessed sarcopenia using L3-SMI or L3-PMI measured by CT, which is recognized as a reliable method to quantify muscle mass. The meta-analysis was conducted following rigorous methodological practice, including duplicate screening, data extraction, assessment of risk of bias using the NOS, and adherence to PRISMA reporting standards. Finally, this systematic review and meta-analysis evaluated the three key outcomes after TIPS (improvement in sarcopenia, overt HE, and mortality) allowing a balanced appraisal of both benefits and risks in patients with cirrhosis and sarcopenia.

Our results show that sarcopenia is associated with higher odds of overt HE after TIPS, whereas TIPS is followed by improvement in sarcopenia in approximately half of patients. This dual effect challenges the view that TIPS is linked only to adverse outcomes in cirrhosis with sarcopenia, suggesting a potential benefit through improvement in sarcopenia (increase in muscle mass indices). Caution is warranted in generalizing these findings to settings where sarcopenia is assessed using methods other than L3-SMI or L3-PMI measured by CT.

Prospective studies are needed to confirm whether patients who experienced improvement in sarcopenia after TIPS are protected from the elevated risk of overt HE and mortality. Identifying predictive factors of improvement in sarcopenia after TIPS is crucial for accurate risk stratification and patient management. Future studies should incorporate standardized sarcopenia definitions; control for confounders, such as nutritional status and rehabilitation, or administration of prophylactic medication for overt HE; and report longitudinal changes in muscle mass and clinical outcomes.

In summary, patients with cirrhosis and sarcopenia undergoing TIPS have higher odds of overt HE, whereas TIPS is also followed by improvement in sarcopenia in approximately half of patients. Recognizing this dual effect is important for clinical decision-making. However, the association between sarcopenia and mortality after TIPS remains uncertain. Our findings support the importance of individualized risk assessment, careful patient selection, and post-procedural monitoring. Sarcopenia and malnutrition should be systematically evaluated with a nutritional assessment before TIPS to guide both prognosis and follow-up strategies.

## Abbreviations

CT, computed tomography; HE, hepatic encephalopathy; HR, hazard ratio; JSH, Japanese Society of Hepatology; L3-PMI, Psoas muscle index at the third lumbar vertebra; L3-SMI, skeletal muscle index at the third lumbar vertebra; LOLA, L-ornithine-L-aspartate; MELD, model for end-stage liver disease; MeSH, Medical subject headings; MRI, magnetic resonance imaging; NOS, Newcastle–Ottawa Scale; OR, odds ratio; PRISMA, Preferred Reporting Items for Systematic Reviews and Meta-analyses; TIPS, Transjugular intrahepatic portosystemic shunt; TPMT, transversal psoas thickness.

## Financial support

MBN was supported by the Swiss Foundation for Liver Diseases.

## Authors’ contributions

Conceptualization: MBN, MGD, AB. Methodology: MBN, AB. Investigation: MGD. Data curation: MBN, MGD. Formal analysis: MBN, JB. Validation: JB. Writing (original draft): MBN, MGD. Writing – review & editing: JB, AB. Critical review for intellectual content: JB, AB. Supervision: AB.

## Data availability

All data underlying this study are included in the article and its supplementary materials. Additional extraction tables are available from the corresponding author upon reasonable request.

## Declaration of generative AI and AI-assisted technologies in the writing process

During the preparation of this work, the authors used language-editing tools, including ChatGPT and Grammarly, to improve spelling and syntax. After using this tool/service, the authors reviewed and edited the content as needed and take full responsibility for the content of the publication.

## Conflicts of interest

The authors have no conflicts of interest to report.

Please refer to the accompanying ICMJE disclosure forms for further details.
