# Supplementary material for: Outcomes after TIPS in patients with cirrhosis and sarcopenia: A systematic review and meta-analysis
Source: JHEP Rep. 2025 Nov 29;8(2):101699. doi: 10.1016/j.jhepr.2025.101699 (PMC12857352; doi:10.1016/j.jhepr.2025.101699)
Supplement: Multimedia component 1 [file mmc1.pdf]

Outcomes after TIPS in patients with cirrhosis and sarcopenia: A systematic review and meta-analysis

Maria de Brito Nunes, Maria Gabriela Delgado, Jaume Bosch, Annalisa Berzigotti

Table of contents

Table S1……………………………………………………………………………………2
Table S2……………………………………………………………………………………6
Table S3…………………………………………………………………………….....12
Table S3…………………………………………………………………………………13
Fig. S1……………………………………………………………………………………22
Fig. S2……………………………………………………………………………………23
Fig. S3……………………………………………………………………………………24
Fig. S4……………………………………………………………………………………25
Fig. S5……………………………………………………………………………………26
Fig. S6……………………………………………………………………………………27
Fig. S7……………………………………………………………………………………28
Fig. S8……………………………………………………………………………………29
Fig. S9……………………………………………………………………………………29
Fig. S10…………………………………………………………………………….....30

Table S1- Search strategy

	**Free Text**	**MeSH (controlled vocabulary)**	**Search**
**P**: patients with cirrhosis and **sarcopenia**	sarcop*.ti,ab,kw.	exp Sarcopenia/	Search 5
**I:** Transjugular Intrahepatic Portosystemic Shunt (TIPS)	(“Transjugular Intrahepatic Portosystemic Shunt” OR “tips” OR “tipss”).ti,ab,kw	exp Portasystemic Shunt, Transjugular Intrahepatic/	Search 10
**O:** Sarcopenia Reversal defined by L3-SMI or L3-PMI improvement, *de novo* Hepatic Encephalopathy, and overall Mortality post-TIPS			

<u>Key articles identified in google scholar:</u>

Nardelli, S., Lattanzi, B., Torrisi, S., Greco, F., Farcomeni, A., Gioia, S., ... & Riggio, O. (2017). Sarcopenia is risk factor for development of hepatic encephalopathy after transjugular intrahepatic portosystemic shunt placement. *Clinical Gastroenterology and Hepatology*, *15*(6), 934-936.

Liu, J., Ma, J., Yang, C., Chen, M., Shi, Q., Zhou, C., ... & Xiong, B. (2022). Sarcopenia in patients with cirrhosis after transjugular intrahepatic portosystemic shunt placement. *Radiology*, *303*(3), 711-719.

Li, T., Liu, J., Zhao, J., Bai, Y., Huang, S., Yang, C., ... & Xiong, B. (2023). Sarcopenia defined by psoas muscle thickness predicts mortality after transjugular intrahepatic portosystemic shunt. *Digestive Diseases and Sciences*, *68*(4), 1641-1652.

Tsien, C., Shah, S. N., McCullough, A. J., & Dasarathy, S. (2013). Reversal of sarcopenia predicts survival after a transjugular intrahepatic portosystemic stent. *European journal of gastroenterology & hepatology*, *25*(1), 85-93.

Benmassaoud, A., Roccarina, D., Arico, F., Leandro, G., Yu, B., Cheng, F., ... & Tsochatzis, E. (2020). Sarcopenia does not worsen survival in patients with cirrhosis undergoing transjugular intrahepatic portosystemic shunt for refractory ascites. *Official journal of the American College of Gastroenterology| ACG*, *115*(11), 1911-1914.

Search Strategy

WITHOUT FILTERS TO ENGLISH

Ovid MEDLINE(R) ALL <1946 to January 31, 2025>

1 exp Sarcopenia/ 9673

2 sarcop*.ti,ab,kw. 42847

3 1 or 2 43606

4 exp Portasystemic Shunt, Transjugular Intrahepatic/ 3322

5 ("Transjugular Intrahepatic Portosystemic Shunt" or "tips" or "tipss").ti,ab,kw. 39694

6 4 or 5 40426

7 3 and 6 82

(exp Sarcopenia/ or sarcop*.ti,ab,kw.) and (exp Portasystemic Shunt, Transjugular Intrahepatic/ or ("Transjugular Intrahepatic Portosystemic Shunt" or "tips" or "tipss").ti,ab,kw.)

Ovid MEDLINE(R) ALL <1946 to January 31, 2025>

1 exp Sarcopenia/ 9617

2 limit 1 to english language 9208

3 sarcop*.ti,ab,kw. 42688

4 2 or 3 41304

5 exp Portasystemic Shunt, Transjugular Intrahepatic/ 3316

6 ("Transjugular Intrahepatic Portosystemic Shunt" or "tips" or "tipss").ti,ab,kw. 39594

7 5 or 6 38577

8 4 and 7 81

Found: 81 articles

PUBMED RESEARCH

QUERY SEARCH 04.02.2025

("Sarcopenia"[MeSH Terms] OR "sarcop*"[Title/Abstract]) AND (Portasystemic Shunt [MeSH Terms] OR Transjugular Intrahepatic[MeSH Terms] OR "Transjugular Intrahepatic Portosystemic Shunt"[Title/Abstract] OR "tips"[Title/Abstract] OR "tipss"[Title/Abstract])

86 results

EMBASE (VIA OVIDSP)

Search Query

('Sarcopenia'/ or sarcop*.ti,ab,kw.) AND ('Portasystemic Shunt, Transjugular Intrahepatic'/ or "Transjugular Intrahepatic Portosystemic Shunt".ti,ab,kw or "tips".ti,ab,kw or "tipss".ti,ab,kw.)

Embase <1974 to January 31, 2025>

('Sarcopenia'/ or sarcop*.ti,ab,kw.) and ('Portasystemic Shunt, Transjugular Intrahepatic'/ or "Transjugular Intrahepatic Portosystemic Shunt".ti,ab,kw. or "tips".ti,ab,kw. or "tipss".ti,ab,kw.) 179

Or

Embase <1974 to January 31, 2025>

1 ('Sarcopenia'/ or sarcop*.ti,ab,kw.) and ('Portasystemic Shunt, Transjugular Intrahepatic'/ or "Transjugular Intrahepatic Portosystemic Shunt".ti,ab,kw. or "tips".ti,ab,kw. or "tipss".ti,ab,kw.) 179

2 'Sarcopenia'/ 21234

3 sarcop*.ti,ab,kw. 55021

4 2 or 3 57907

5 'Portasystemic Shunt, Transjugular Intrahepatic'/ 5912

6 "Transjugular Intrahepatic Portosystemic Shunt".ti,ab,kw. 4779

7 "tips".ti,ab,kw. 47698

8 "tipss".ti,ab,kw. 680

9 6 or 7 or 8 49413

10 4 and 9 143

COCHRANE LIBRARY:

Search 04.02.2025

Trials and reviews

"Sarcopenia" OR sarcop* AND "Portasystemic Shunt, Transjugular Intrahepatic" OR "Transjugular Intrahepatic Portosystemic Shunt" OR tips OR tips

clinicaltrials.gov

search: 03.02.2025

condition/Disease: Sarcopenia in Liver Cirrhosis

intervention/treatment: Transjugular intrahepatic portosystemic shunt \(TIPS\)

NO FILTERS USED

all studies, 3 studies found

EU clinical Trials

0 STUDIES FOUND, No filters used

Table S2- Risk bias assessment considering the selected studies (Newcastle-Ottawa Scale for cohort studies)

	**Representativeness of the exposed cohort**	**Selection of the non-exposed cohort**	**Ascertainment of exposure**	**Demonstration that outcome of interest was not present at start of study**	**Comparability**	**Assessment of outcome**	**Was follow-up long enough for outcomes to occur**	**Adequacy of follow-up of cohorts**	**TOTAL SCORE**
The Added Value of Sarcopenia on Existing Risk Scores to Predict Mortality after TIPS Placement: A Multicenter Study.	*	No applicable (NA)	*	*	*	*	*	*	7
Transjugular intrahepatic portosystemic shunt insertion improves muscle mass but not muscle function or frailty measures.	*	NA	*	*	*	*	*	*	7

Improvement of sarcopenia is beneficial for prognosis in cirrhotic patients after TIPS placement.	*	*	*	*	**	*	*	*	9
Post-transjugular Intrahepatic Portosystemic Shunt HepaticEncephalopathy: Sarcopenia Adds Insult to Injury	*	NA	*	*	**	*	*		7
Predictors of Improvement of Sarcopenia after Transjugular Intrahepatic Portosystemic Shunt Creation in Cirrhotic Patients		NA	*	*	*	*	*	*	6
TIPS placement as the first-line therapy to prevent variceal rebleeding in patients with cirrhosis and sarcopenia.	*	*	*	*	**	*	*		8
Inclusion of sarcopenia improves the prognostic value	*	*	*	*	**	*	*	*	9

of MELD score in patients after transjugular intrahepatic portosystemic shunt									
Sarcopenia in Patients with Cirrhosis after Transjugular Intrahepatic Portosystemic Shunt Placement.	-	NA	*	*	*	*	*	*	6
The improvement in body composition including subcutaneous and visceral fat reduces ammonia and hepatic encephalopathy after transjugular intrahepatic portosystemic shunt	-	NA	*	*	*	*	*	*	7
Sarcopenia Does Not Worsen Survival in Patients With Cirrhosis Undergoing Transjugular Intrahepatic Portosystemic Shunt	*	NA	*	*	*	*	*	*	7

for Refractory Ascites.									
Study 11 The modification of quantity and quality of muscle mass improves the cognitive impairment after TIPS.	*	NA	*	*	*	*	*	*	7
Sarcopenia Is Risk Factor for Development of Hepatic Encephalopathy After Transjugular Intrahepatic Portosystemic Shunt Placement.	*	NA	*	*	*	*	*	*	7
Increase of radiologically determined muscle area in patients with liver cirrhosis after transjugular intrahepatic portosystemic shun	*	NA	*	*	*	*	*	-	6
Sarcopenia Defined by Psoas Muscle Thickness Predicts Mortality After TIPS	*	NA	*	*	*	*	*	-	6
	-	NA	-	*	-	*	*	*	4

Effects of transjugular intrahepatic portosystemic shunt on abdominal muscle mass in patients with decompensated cirrhosis									
Development and Validation of Prognostic Models to Estimate the Risk of Overt Hepatic Encephalopathy After TIPS Creation: A Multicenter Study	*	NA	*	-	*	*	*	*	6
Low adipose tissue index as an indicator of hepatic encephalopathy in cirrhotic patients following transjugular intrahepatic portosystemic shunt.	*	NA	*	-	*	*	*	*	6
Sarcopenia is an independent risk factor for short-term mortality in patients undergoing transjugular	*	*	*	*	*	*	*	*	8

intrahepatic portosystemic shunt									
Body Compositions Correlate With Overt Hepatic Encephalopathy after Transjugular Intrahepatic Portosystemic Shunt	-	*	*	*	*	*	*	*	7
Validating the prognostic value of muscle changes in patients with cirrhosis undergoing transjugular intrahepatic portosystemic shunt	-	*	*	*	*	*	*	*	7

Table S3- GRADE Summary of Findings

Outcome	No. of Studies (Patients)	Effect Estimate (95% CI)	Certainty of Evidence	Main Limitations
**Sarcopenia improvement after TIPS**	10 studies (1,008 patients; 596 with sarcopenia)	-57% improvement (95% CI: 48–65%)<br>-Mean SMI ↑ 4.53 cm^2/m^2	●●○○ Low	Observational design; substantial heterogeneity (I^2 = 68.5%); limited subgroup analyses
**Overt hepatic encephalopathy after TIPS**	11 studies (1,839 patients; 1,080 with sarcopenia)	-Sarcopenia vs. no sarcopenia: OR = 3.40 (95% CI: 1.85–6.25), $p < 0.001$<br>-Incidence: 43% vs. 12%	●●○○ Low	Observational studies; high heterogeneity (I^2 = 85.7%); visual funnel asymmetry (not confirmed)
**Mortality after TIPS**	8 studies (1,194 patients; 211 deaths)<br>5 with HRs	-HR = 1.95 (95% CI: 0.89–4.31), p = 0.078<br>-Per unit ↑ SMI: HR = 0.97 (95% CI: 0.88–1.06), p = 0.34	●●○○ Low	Wide CIs; non-significant results; substantial heterogeneity (I^2 = 71.7%); limited stratified data

Note: The certainty of evidence was assessed using the GRADE approach. All outcomes start at low certainty due to observational study designs. Further downgrades were applied for inconsistency and imprecision where applicable.

Table S4 – Baseline characteristics of the included studies

Study	Title	Authors	Year of publication	Design	Sample size	Population characteristics	Indication of TIPS	Duration of follow-up	Main Outcome
1	The Added Value of Sarcopenia on Existing Risk Scores to Predict Mortality after TIPS Placement: A Multicenter Study.	Xiong, B. et al	2023	Retrospective	386 derivation cohort 198 validation cohort	Derivative cohort gender: 249 (64.5%) male Etiology: 228 (59.1) Hepatitic B CHILD 7.5 MELD score 11.5<br><br>validation cohort gender: 120 (60.6%) male Etiology: 61 (52.1%) Hepatitic B CHILD 7.28 MELD score 11.3	Derivative cohort: variceal bleeding: 349 refractory ascites: 37	24 months (1,3,6, 12 months after TIPS Then annually until OLT, death or end of the study)	FIPS score was highly correlated with the severity of sarcopenia and sarcopenia reversal after TIPS.<br><br>Sarcopenia could improve the prognostic ability of existing scores
2	Transjugular intrahepatic portosystemic shunt insertion improves muscle mass but not muscle function or frailty measures.	Hey, P. et al	2023	Prospective	12	Gender: 8 (67%) male Etiologie: 50% ArLD, 25% MASLD MELD score 16 ± 5	Refractory Ascites: 11 patients recurrent variceal bleeding: 1 patient	6 months	Muscle mass increased following TIPS insertion
3	Improvement of sarcopenia is beneficial for prognosis in cirrhotic patients after TIPS placement.	Liu, J. et al	2023	Retrospective	109	Gender: male 79 (72.5%) Etiology: HBV 71 (65%) CHILD 7.0 (6.0 - 8.0)	Varicela bleeding:103 (94.5%) refractory ascites: 6 (5.5%)	60 Months (1 month, 3 months, 6 months and every 1 year thereafter)	Reversal of sarcopenia or significant SMI improvement by TIPS was associated with a reduced risk

					MELD score: 11.4 ± 3.6			of death and overt HE.
4	Post-transjugular Intrahepatic Portosystemic Shunt HepaticEncephalopathy: Sarcopenia Adds Insult to Injury	Kapoor, P. et al	2023	Retrospective	79	Gender: male 68 (86%)<br>Etiology: ArLD (56%), MASH 16 (20%)<br>CHILD A/B/C: 3 (4%) / 56 (71%) / 20 (25%)<br>MELD score 15.75 ± 6.38	refractory ascites 56 (71%)<br>Variceal bleed 23 (29%)	± 9 months	Increased skeletal muscle index post-TIPS is associated with decreased risk of hepatic encephalopathy
5	TIPS placement as the first-line therapy to prevent variceal rebleeding in patients with cirrhosis and sarcopenia.	Xiong, B. et al	2023	Retrospective	262	Total<br>gender: 173 (66%) male<br>Etiology: Hepatitis B:157 (59.9%)<br>CHILD 7.4 (1.5)<br>MELD 11.2 (3.4)<br>MELD Na score 12.2 (4.3)<br><br>Non-sarcopenia<br>gender: 94 (57.7%) male<br>Etiology: Hepatitis B: 93 (57.1%)<br>CHILD 7.2 (1.4)<br>MELD 10.7 (2.8)<br>MELD Na score 11.8 (4.0)	Prevention of rebleeding	36 Months (1, 3, 6, 12 months after TIPS. Then annually until OLT, death or end of the study)	Baseline sarcopenia did not increase the risk of post-TIPS mortality and overt HE

						Sarcopenia gender: 79 (79.8%) male<br>Etiology: Hepatitis B: 64 (64.6%)<br>CHILD 7.8 (1.6)<br>MELD 12 (3.9)<br>MELD Na score 12.9 (4.6)			
6	Increase of radiologically determined muscle area in patients with liver cirrhosis after transjugular intrahepatic portosystemic shunt	March, C. et al	2023	Retrospective	52	Gender: 42 (80.8%) male<br>Etiology: ArLD (69.2%), MASH<br>CHILD A/B/C: 3 (7.7%)/26 (66.7%)/ 10 (25.6%)<br>MELD score 12.8 ± 4.6<br>Sarcopenia Pre-TIPS: 84.6% men, 92.3% women	Refractory ascites: 39 (75%), variceal bleeding: 11 (21.1%)	median follow-up of 16.5 months (6-93)	TIPS procedure has a positive impact on muscle area in patients with liver cirrhosis and sarcopenia. Higher albumin levels were observed in patients with increased SMI after TIPS compared to patients without an increase
7	Low adipose tissue index as an indicator of hepatic encephalopathy in cirrhotic patients following transjugular	Wang, C. et al	2023	Retrospective	191	Gender: 116 male<br>Etiology: HBV 79 (41.4%), Schistosomiasis 42 (22%)<br>CHILD 7.4 ± 1.7<br>MELD score 10.7 ±	variceal bleeding (179, 93.7%)	median follow-up time was 28 months	Body composition indexes (VFAI, SFAI, SMI) could be utilized to predict the HE

						3.4<br>MELD-Na score 11.3 ± 4.2<br>Sarcopenia Pre-TIPS: 73 (38.2%) patients			risk of cirrhotic patients after TIPS
8	Sarcopenia Defined by Psoas Muscle Thickness Predicts Mortality After TIPS	Li, T. et al	2023	Retrospective	249	Gender: 162 (65.1%) male<br>Etiology: HBV 150 (60.2%)<br>CHILD: 7.5 ± 1.58<br>MELD score 11.78 ± 1.58 (A: 62/B 164/ C 23 patients)<br>Meld-Na score: 12.81 ± 5.0<br>Sarcopenia Pre-TIPS 82 (32.9%) patients	variceal bleeding (219, 88%), refractory ascites (3, 12%)	mean follow-up time was 22.4 ± 15.9 months (1, 6, and 12 months, every 12 months)	Overall survival was significantly lower in the sarcopenia group
9	Predictors of Improvement of Sarcopenia after Transjugular Intrahepatic Portosystemic Shunt Creation in Cirrhotic Patients	Huang, S. et al	2022	Retrospective	111	Gender: 81 (73%) male<br>Etiology: HBV 71 (64%)<br>CHILD 7.5 ± 1.5<br>MELD score 11.4 ± 3.6	Variceal bleeding: 105 patients refractory ascites: 6 patients	6 months	Pre-TIPS SMI and changes in portal pressure gradient were found to be independent risk factor for experiencing substantial improvement in post-TIPS SMI.

10	Inclusion of sarcopenia improves the prognostic value of MELD score in patients after transjugular intrahepatic portosystemic shunt	Bai, Y-W. et al	2022	Retrospective	289	Total<br>Gender: 191 (66.1%) male<br>Etiology: Hepatitis B:187 (64.7%)<br>CHILD 7.6 ± 1.8<br>MELD 11.9 ± 4.0<br>MELD Na score 12.8 ± 5.1<br><br>Non-sarcopenia<br>Gender: 93 (61.6%) male<br>Etiology: Hepatitis B: 97 (64.2%)<br>CHILD 7.3±1.8<br>MELD 11.3 ± 3.4<br>MELD Na score 12.1 ± 4.5<br><br>Sarcopenia<br>Gender: 98 (71%) male<br>Etiology: Hepatitis B: 90 (65.2%)<br>CHILD 7.9 ± 1.8<br>MELD 12.6 ± 4.4<br>MELD Na score 13.7 ± 5.5	Variceal bleeding: 256 (88%), refractory ascites 33 (11.4%)	24 months	Sarcopenia is independently correlated with post-TIPS mortality.<br>MELD-Sarcopenia score showed the best performance in predicting post-TIPS mortality than the traditional predictive models

11	Sarcopenia in Patients with Cirrhosis after Transjugular Intrahepatic Portosystemic Shunt Placement.	Liu, J. et al	2022	Retrospective	224	Total gender: 159 male<br>Etiology: Hepatitis B:153 (68%)<br>CHILD 7.7 ± 1.6<br>MELD 11.9 ± 3.9<br>MELD Na score 12.9 ± 4.8<br>Sarcopenia Pre-TIPS: 145 patients	Variceal bleeding: 193 (86.2%), refractory ascites 31 (13.8%)	12 Months (2, 5 and 12 months after TIPS)	In cirrhotic patients with sarcopenia, skeletal muscle, and fat mass increased after TIPS placement.<br>The reversal of sarcopenia could reduce the mortality
12	Development and Validation of Prognostic Models to Estimate the Risk of Overt Hepatic Encephalopathy After TIPS Creation: A Multicenter Study	Yang, C. et al	2023	Retrospective	Derivation cohort: 276<br>Validation cohort 182	Derivation cohorte: Gender: 181 (65.6%) male<br>Etiology. Viral 187 (67.8%)<br>CHILD 7.68 (1.67)<br>MELD score 11.8 (3.86)<br>MELD-Na score 12.6 (4.77)<br>Sarcopenia Pre-TIPS: 170 (61.6%) patients	variceal bleeding (248, 89.9%), refractory ascites (28, 10.1%)	3 months	Sarcopenia was associated with an increased risk of post-TIPS overt HE
13	The improvement in body composition including subcutaneous and visceral fat reduces ammonia and hepatic encephalopathy after transjugular	Gioia, S. et al	2021	Retrospective	35	Gender: 28 male<br>etiology:Alcohol 16 patients, viral 12 patients<br>CHILD (A/B/C): 17/16/2<br>MELD score 11.9 ± 4	variceal bleeding (16 patients), refractory ascites (19 patients)	mean follow-up 19 ± 15 months (1,3, 6 month and every 6 months)	Overt HE was significantly lower in the patients with improved SMI and in

	intrahepatic portosystemic shunt					sarcopenia Pre-TIPS 19 patients			the patients with improved subcutaneous adipose tissue
14	Sarcopenia Does Not Worsen Survival in Patients With Cirrhosis Undergoing Transjugular Intrahepatic Portosystemic Shunt for Refractory Ascites.	Benmassaoud, A. et al	2020	Retrospective	107	Total<br>Gender: 65 (60.71%) male<br>Etiology: ArLD: 69 (64.5%)<br>CHILD 8 (2) /B: 96, C:11<br>MELD Na score 11 (8)<br><br>Non-sarcopenia<br>Gender: 20 (43.5%) male<br>Etiology: ArLD: 26 (56.5.2%)<br>CHILD 8 (2)<br>MELD Na score 12 (6)<br><br>Sarcopenia<br>Gender: 45 (73.8%) male<br>Etiology: ArLD: 43 (70.5%)<br>CHILD 8 (2)<br>MELD Na score 11 (8)	refractory ascites	5 years	Sarcopenia<br><br>is not associated with de novo HE or increased mortality post-TIPS

15	The modification of quantity and quality of muscle mass improves the cognitive impairment after TIPS.	Gioia, S. et al	2019	Retrospective	27	Gender: 23 male<br>Etiology: Viral 12, ArLD 8<br>CHILD 7.1 ± 13<br>MELD score 11.3 ± 4.2<br>Sarcopenia Pre-TIPS: 21 (78%) patients	refractory ascites (15 patients), variceal bleeding (12 patients)	8.3 months (range 5.8-19)	Overt HE was significantly lower in the patients with the improvement of sarcopenia during the follow-up
16	Sarcopenia Is Risk Factor for Development of Hepatic Encephalopathy After Transjugular Intrahepatic Portosystemic Shunt Placement.	Nardelli, S. et al	2017	Prospective	46	Gender: 34 male<br>Etiology: ArLD, viral<br>CHILD 7.6 ± 1.5<br>MELD score 11.3 ± 3.3<br>Sarcopenia Pre-TIPS: 26 (57%) patients	variceal bleeding, refractory ascites (equal)	7 ± 9 months	MELD and sarcopenia were associated independently with the development of HE after TIPS placement
17	Sarcopenia is an independent risk factor for short term mortality in patients undergoing transjugular intrahepatic portosystemic shunt	Stoffel, E. et al	2024	Retrospective	232	Gender: 105 male<br>Etiology: hepatitis C, ArLD, MASH<br>Sarcopenia Pre-TIPS: 136 patients<br>MELD score12.82 ± 4.08	variceal bleeding (29.74%), refractory ascites (47.84%)	12 months or until death	Sarcopenia was an independent risk factor for 1-year mortality in patients undergoing TIPS
18	Body Compositions Correlate With Overt Hepatic Encephalopathy after Transjugular	Shi, W. et al	2024	Retrospective	571	Gender: 439 male<br>Etiology: hepatitis, ArLD, cholestatic	variceal bleeding and ascites	12 months	SATI and myosteatosis are independent risk factors for

	Intrahepatic Portosystemic Shunt								post-TIPS overt HE
19	Validating the prognostic value of muscle changes in patients with cirrhosis undergoing transjugular intrahepatic portosystemic shunt	Wen, Z. et al	2024	Retrospective	202	Gender:109 male<br>Etiology: viral hepatitis, ArLD, autoimmune<br>MELD: 10.0 (8.0-12.0)<br>CHILD A 36.6%, CHILD B 58.4%, CHILD C 5%	Variceal bleeding (99%)	Median of 33.6 months	The presence of muscle changes before TIPS significantly increased the risk of post-TIPS mortality but did not increase the risk of decompensation events
20	Effects of Transjugular intrahepaticpostosystemic shunt on abdominal muscle mass in patients with decompensated cirrhosis	Wu et al	2023	Retrospective	25	Gender: 19 male<br>Etiology: viral hepatitis, alcohol liver disease<br>MELD 11<br>CHILD A 32%, CHILD B 56%, CHILD C 12%	Variceal bleeding and refractory ascites and hydrothorax	6 and 12 months	The combination of sarcopenia and reduced BMI were prognostic factors for reduced overall survival in multivariate analysis

TIPS: transjugular intrahepatic portosystemic shunt. ArLD: Alcohol related liver disease. MASLD: metabolic dysfunction associated steatotic liver disease. VFAI: visceral fat area index subcutaneous fat area index. SFAI: subcutaneous fat area index, SMI: skeletal muscle index, SATI: subcutaneous adipose tissue

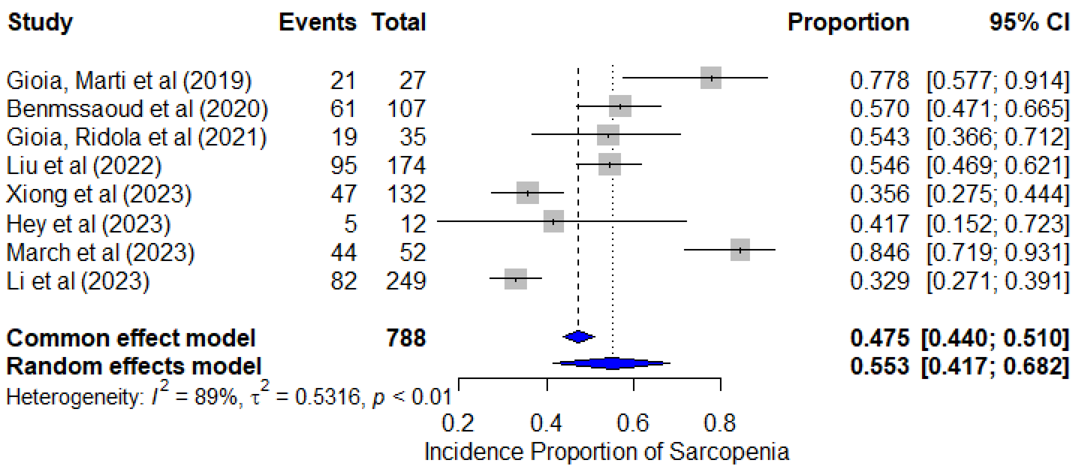

Fig. S1- Forest plot illustrating the pooled prevalence of sarcopenia across the studies. Proportions with 95% confidence intervals (CIs) are shown for each study.

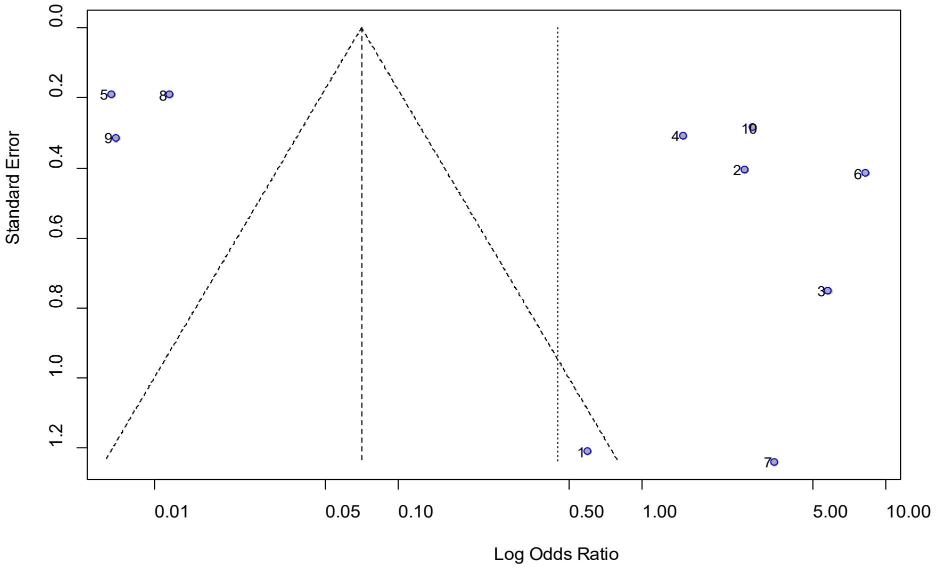

Fig. S2 - Funnel plot assessing potential publication bias among studies evaluating the proportion of patients demonstrating improvement in sarcopenia after TIPS. Asymmetry in the distribution of studies may suggest the presence of publication bias or small-study effects.

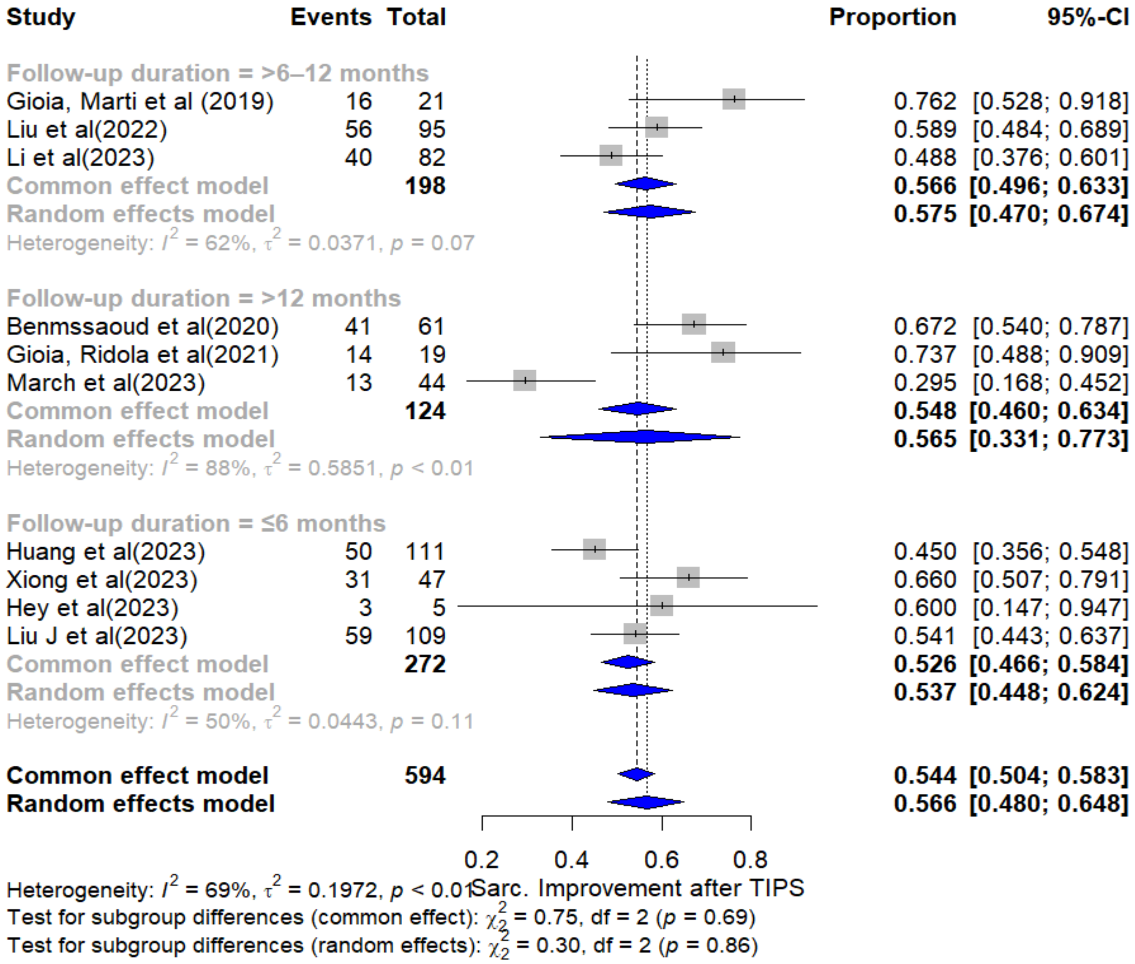

Fig. S3-Forest plot showing a pooled and subgroup analysis of the proportion of sarcopenia improvement after TIPS, with results stratified by follow-up duration. Proportions with 95% confidence intervals (CIs) are shown for each study.

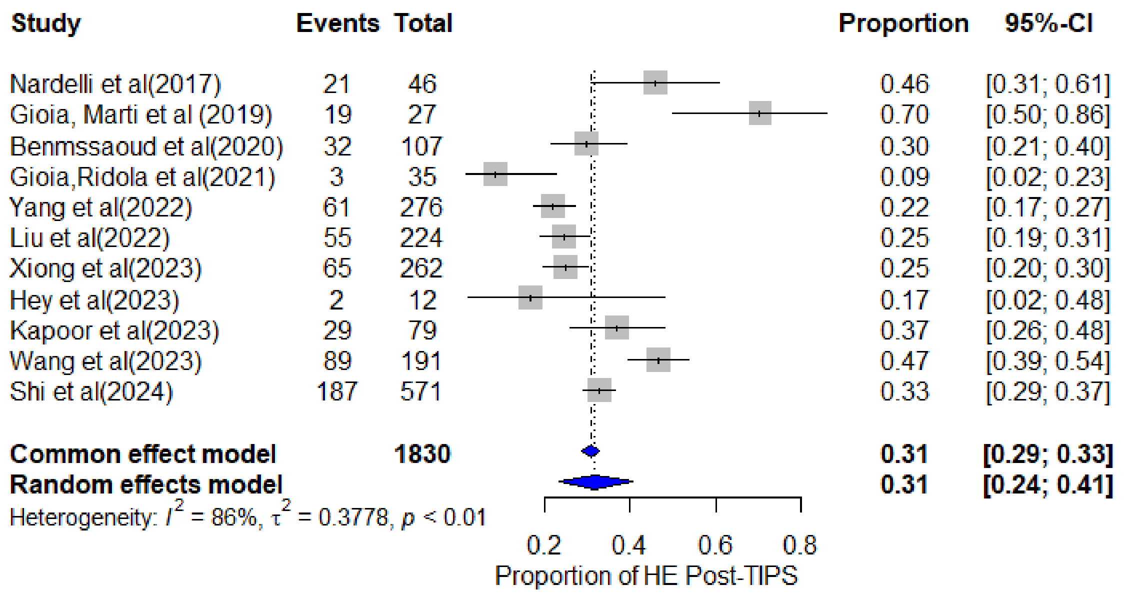

Fig. S4-Forest plot showing the proportion of hepatic encephalopathy after TIPS. Proportions with 95% confidence intervals (CIs) are shown for each study.

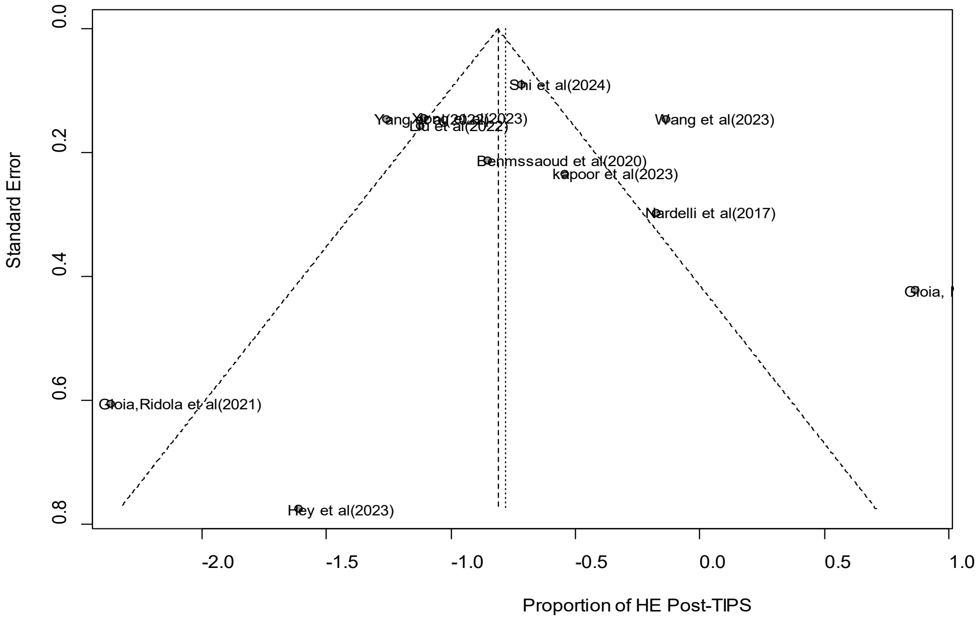

Fig. S5-Funnel plot assessing potential publication bias among studies evaluating the proportion of HE after TIPS in patients with and without sarcopenia. Asymmetry in the distribution of studies may suggest the presence of publication bias or small-study effects.

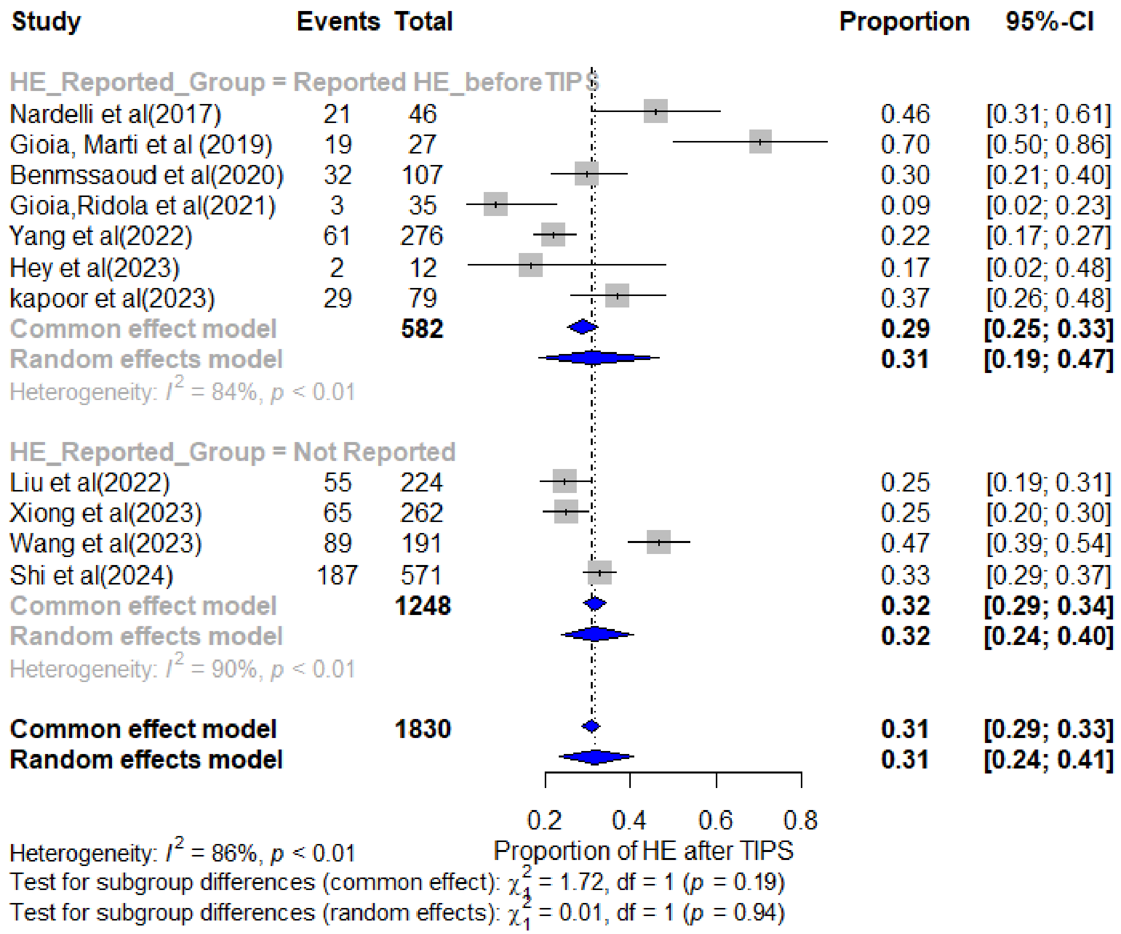

Fig. S6- Forest plot of subgroup analysis comparing studies reporting and studies not reporting presence of HE before TIPS. Proportions with 95% confidence intervals (CIs) are shown for each study.

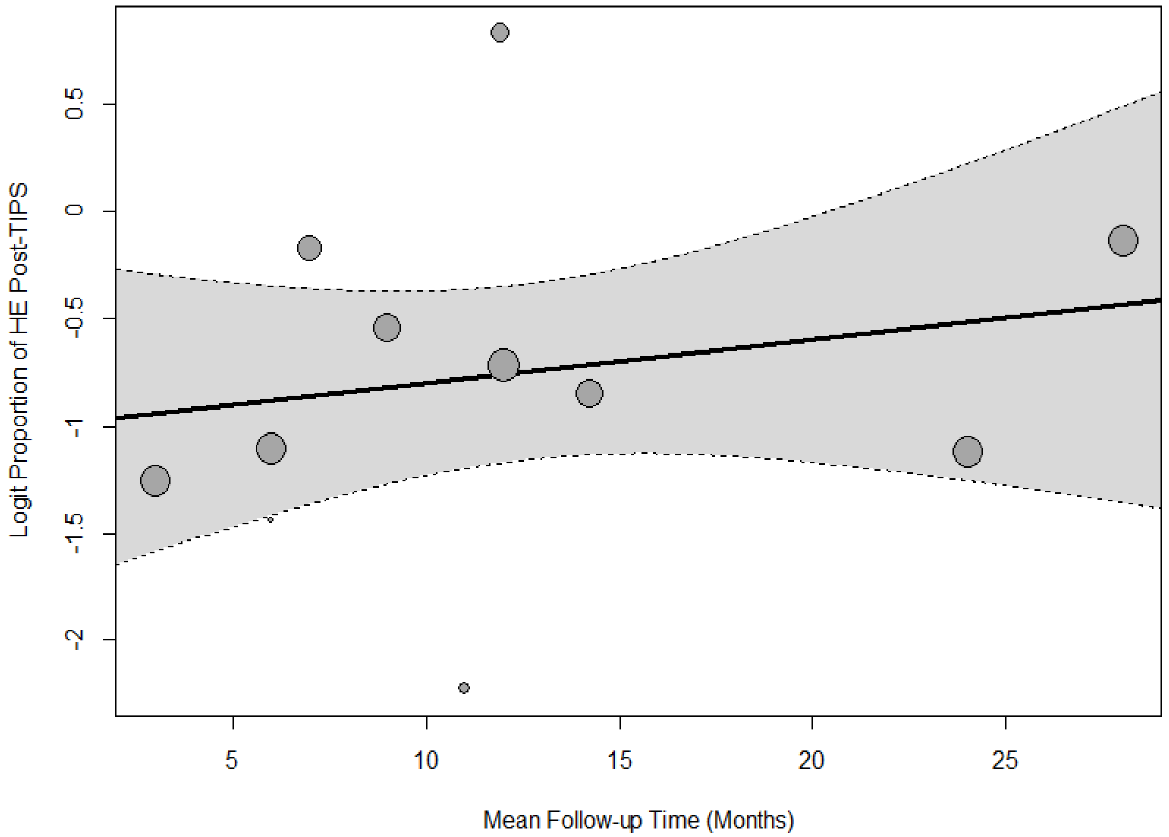

Fig. S7- Bubble plot showing a meta-regression analysis of the association between mean follow-up duration (months) and the logit-transformed proportion of hepatic encephalopathy (HE) events after TIPS. Each circle represents an individual study, with circle size proportional to the study weight in the analysis. The solid line represents the fitted regression line, and the shaded area indicates the 95% confidence interval.

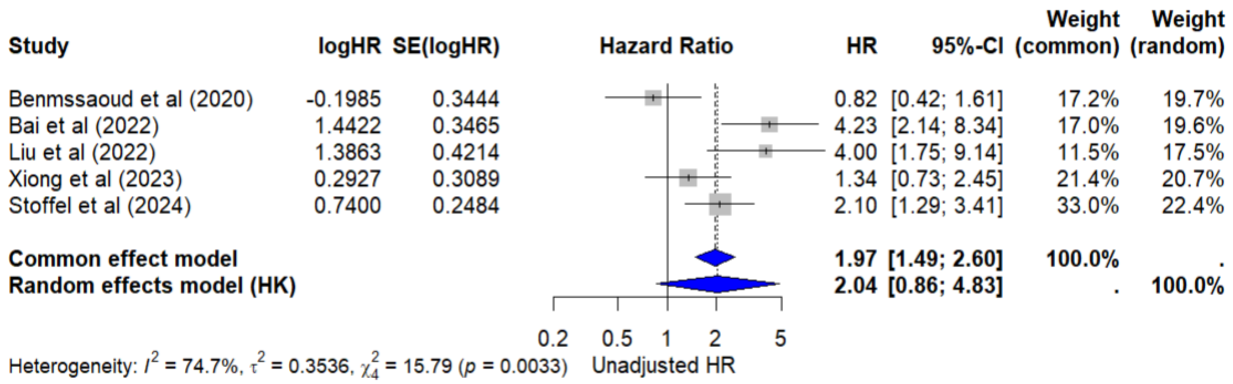

Fig. S8- Forest plot showing unadjusted Hazard Ratios (HR) for the association between sarcopenia and mortality after TIPS. Unadjusted HR was obtained in each study by univariate cox regression model. HR with 95% confidence intervals (CIs) are shown for each study.

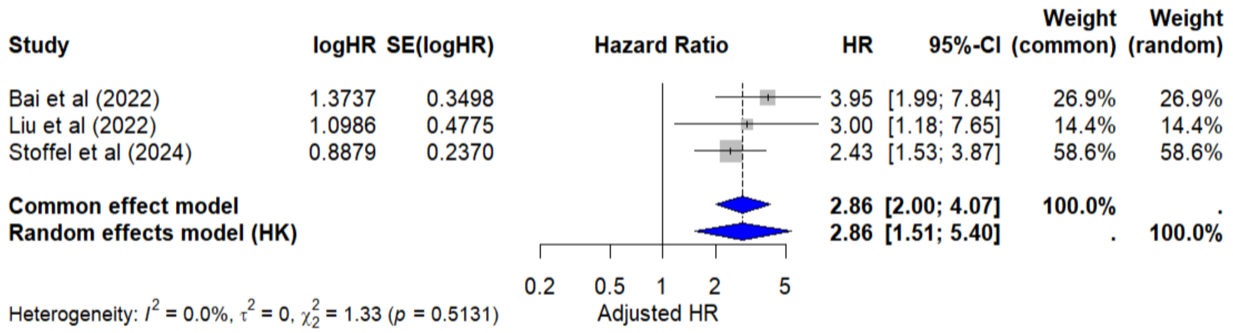

Fig. S9- Forest plot showing adjusted Hazard Ratios (HR) for the association between sarcopenia and mortality after TIPS. Adjusted HR was obtained in each study by multivariate cox regression model. HR with 95% confidence intervals (CIs) are shown for each study.

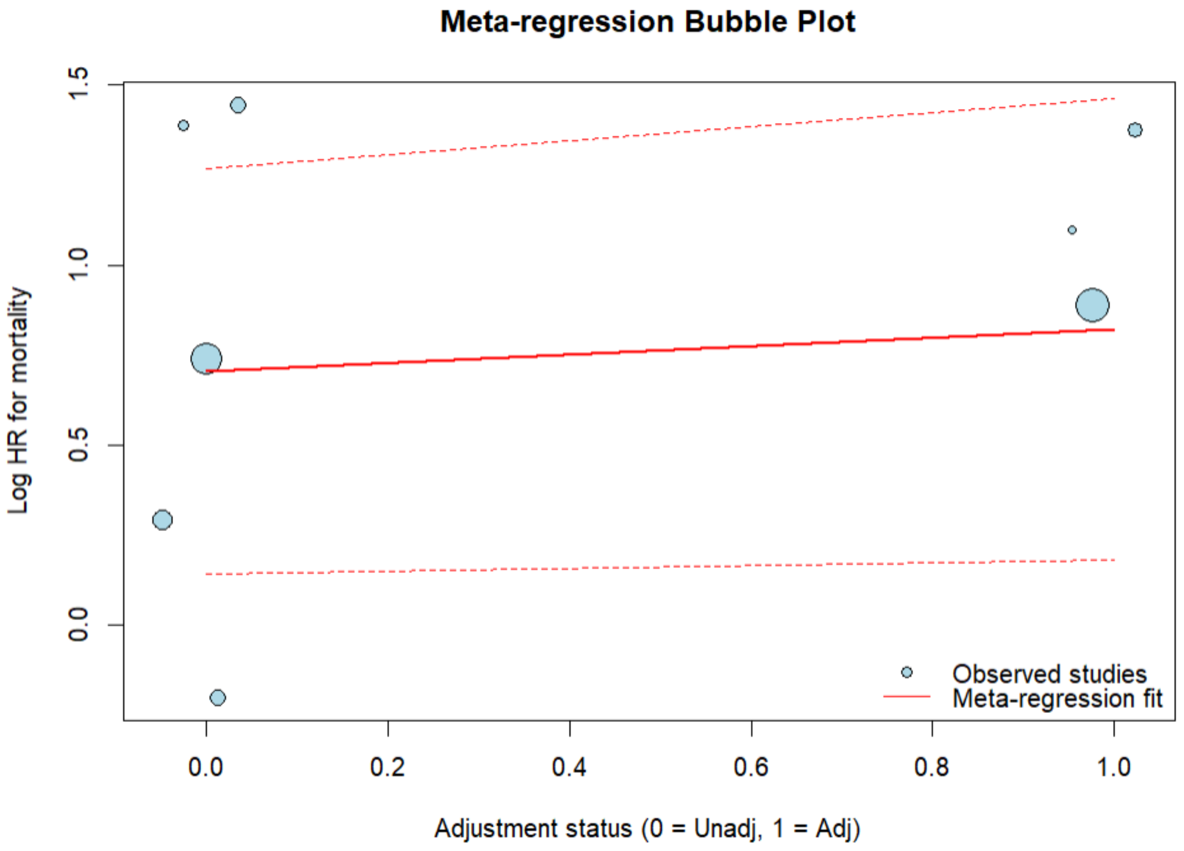

**Multivariate Meta-Analysis Model (k = 8; method: REML)**	logLik Deviance AIC BIC AICc<br>-4.5456 9.0912 15.0912 14.4665 27.0912
**Variance Components**	estim sqrt nlvls fixed factor

	sigma^2 0.3049 0.5521 5
**Test for Residual Heterogeneity**	QE(df = 6) = 17.1209, p-val = 0.0088
**Test of Moderators (coefficient 2)**	QM(df = 1) = 0.2161, p-val = 0.6420
**Model Results**	**estimate se zval pval ci.lb ci.ub**<br>**intrcpt 0.7051 0.2873 2.4546 0.0141 0.1421 1.2681 ***<br>**adjusted 0.1169 0.2515 0.4649 0.6420 -0.3760 0.6098**

Fig. S10- Meta-regression bubble plot showing hazard ratios (HRs) for mortality after TIPS according to adjustment status. Each bubble represents an individual study's log-transformed HR, with bubble size proportional to the study's weight (inverse of variance). The horizontal axis indicates the adjustment status (0 = unadjusted; 1 = multivariate adjusted). The solid red line shows the fitted meta-regression slope, with dashed lines representing its 95% confidence interval. The regression line is almost horizontal, reflecting that multivariate adjustments did not modify the association between sarcopenia and mortality risk. This non-significant slope indicates that adjustment for confounders did not influence the reported HRs (β = 0.12; 95% CI: –0.38 to 0.61; p = 0.64).
