# Supplementary material for: Outcomes after TIPS in patients with cirrhosis and sarcopenia: A systematic review and meta-analysis
Source: JHEP Rep. 2025 Nov 29;8(2):101699. doi: 10.1016/j.jhepr.2025.101699 (PMC12857352; doi:10.1016/j.jhepr.2025.101699)
Supplement: Multimedia component 2 [file mmc2.docx]

**JHEP Reports**

**CTAT methods**

Tables for a “Complete, Transparent, Accurate and Timely account” (CTAT) are now mandatory for all revised submissions. The aim is to enhance the reproducibility of methods.

- Only include the parts relevant to your study
- Refer to the CTAT in the main text as ‘Supplementary CTAT Table’
- Do not add subheadings
- Add as many rows as needed to include all information
- Only include one item per row

**If the CTAT form is not relevant to your study, please outline the reasons why:**

| This submission is a systematic review and meta-analysis of published studies. We did not use laboratory methods, cell lines, organisms, sequence-based reagents, or biological samples. Accordingly, sections 1.1–1.5 are not applicable. |
| --- |

- 1. **Antibodies**

| **Name** | **Citation** | **Supplier** | **Cat no.** | **Clone no.** |
| --- | --- | --- | --- | --- |
|  |  |  |  |  |

- 1. **Cell lines**

| **Name** | **Citation** | **Supplier** | **Cat no.** | **Passage no.** | **Authentication test method** |
| --- | --- | --- | --- | --- | --- |
|  |  |  |  |  |  |

- 1. **Organisms**

| **Name** | **Citation** | **Supplier** | **Strain** | **Sex** | **Age** | **Overall n number** |
| --- | --- | --- | --- | --- | --- | --- |
|  |  |  |  |  |  |  |

- 1. **Sequence based reagents**

| **Name** | **Sequence** | **Supplier** |
| --- | --- | --- |
|  |  |  |

- 1. **Biological samples**

| **Description** | **Source** | **Identifier** |
| --- | --- | --- |
|  |  |  |

- 1. **Deposited data**

| **Name of repository** | **Identifier** | **Link** |
| --- | --- | --- |
|  |  | Data were extracted from published articles; extraction sheets and analytic code available upon reasonable request to the corresponding author. |

- 1. **Software**

| **Software name** | **Manufacturer** | **Version** |
| --- | --- | --- |
| **R studio** | **R foundation for statistical computing** | **4.2.3** |

- 1. **Other (*e.g*. drugs, proteins, vectors etc.)**

|  |  |  |
| --- | --- | --- |
|  |  |  |

- 1. **Please provide the details of the corresponding methods author for the manuscript:**

| \| **Field** \| **Details** \| \| --- \| --- \| \| Name \| **Maria de Brito Nunes, MD** \| \| Affiliation \| Hepatology, University Clinic for Visceral Surgery and Medicine, Bern University Hospital, University of Bern, Switzerland \| \| Address \| MEM F808, Murtenstrasse 35, CH-3008 Bern, Switzerland \| \| Telephone \| +41 31 632 30 26 \| \| Email \| maria.debritorodriguesnunes@unibe.ch \| |
| --- | --- | --- | --- | --- | --- | --- | --- | --- | --- | --- | --- | --- |

**2.0 Please confirm for randomised controlled trials all versions of the clinical protocol are included in the submission. These will be published online as supplementary information.**

| Not applicable. This work is a systematic review and meta-analysis; no randomized controlled trial was conducted by the authors. The study protocol was registered in PROSPERO (CRD42025646782) and is cited in the manuscript. |
| --- |
