# Supplementary material for: Outcomes after TIPS in patients with cirrhosis and sarcopenia: A systematic review and meta-analysis
Source: JHEP Rep. 2025 Nov 29;8(2):101699. doi: 10.1016/j.jhepr.2025.101699 (PMC12857352; doi:10.1016/j.jhepr.2025.101699)
Supplement: Multimedia component 4 [file mmc4.pdf]

Outcomes after TIPS in patients with cirrhosis and sarcopenia: A systematic review and meta-analysis

Authors

Maria de Brito Nunes, Maria Gabriela Delgado, Jaume Bosch, Annalisa Berzigotti

Correspondence

maria.debritorodriguesnunes@unibe.ch (M. de Brito Nunes).

Graphical abstract

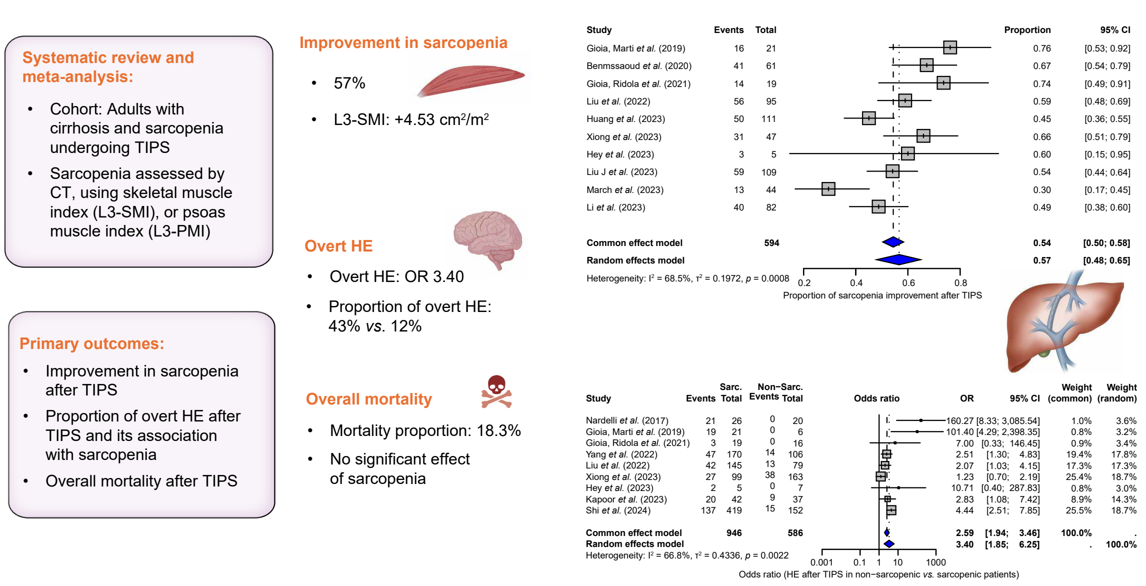

Highlights:

- Sarcopenia is common in patients with cirrhosis undergoing TIPS (~55% prevalence).
- TIPS improves sarcopenia in 57% of cases.
- Pre-existing sarcopenia is associated with a 3-fold increased risk of overt HE after TIPS (pooled OR 3.40, 95% CI 1.85–6.25, p <0.001).
- Overall mortality after TIPS is 18% (95% CI 14.6–22.8%).

Impact and implications:

Sarcopenia affects over half of patients with cirrhosis undergoing transjugular intrahepatic portosystemic shunt (TIPS) placement. This meta-analysis shows that TIPS is associated with sarcopenia improvement in >50% of patients, suggesting potential benefits beyond portal pressure reduction. Sarcopenia increases the risk of overt hepatic encephalopathy, whereas its effect on post-TIPS mortality remains inconclusive. These findings support routine assessment of sarcopenia to improve risk stratification and clinical decision-making. However, results should be interpreted cautiously because of study heterogeneity and the retrospective nature of the included data. Prospective studies are needed to confirm these findings and refine patient selection for TIPS.

https://doi.org/10.1016/j.jhepr.2025.101699
© 2025 The Authors. Published by Elsevier B.V. on behalf of European Association for the Study of the Liver (EASL). This is an open access article under the CC BY license (http://creativecommons.org/licenses/by/4.0/). JHEP Reports, 2026, 8, 1–10

Outcomes after TIPS in patients with cirrhosis and sarcopenia: A systematic review and meta-analysis

Maria de Brito Nunes[1,2,3,*,†], **Maria Gabriela Delgado**[1,†], Jaume Bosch[1], Annalisa Berzigotti[1]

JHEP Reports **2026**. vol. 8 | 1–10

Background & Aims: Transjugular intrahepatic portosystemic shunt (TIPS) is an established treatment for complications of portal hypertension (variceal bleeding and refractory ascites). Sarcopenia affects 40–70% of patients with cirrhosis. We evaluated the improvement in sarcopenia after TIPS, the proportion of patients developing overt hepatic encephalopathy (HE) after TIPS and its association with sarcopenia, and the association between sarcopenia and mortality after TIPS.

Methods: We conducted a systematic review and meta-analysis according to the Preferred Reporting Items for Systematic Reviews and Meta-analyses (PRISMA) guidelines. Eligible studies included adults with cirrhosis and sarcopenia assessed by computed tomography (CT), using the skeletal muscle index (L3-SMI), or psoas muscle index (L3-PMI). We searched in eight databases for randomized trials, cohort studies, and case–control, cross-sectional, and case series studies.

Results: Twenty studies were included. The pooled prevalence of sarcopenia in patients with cirrhosis undergoing TIPS was 55.4% (95% CI 41.7–68.2%). Among these, sarcopenia improved in 57% of patients (95% CI 48–65%) after TIPS, with a mean L3-SMI increase of 4.53 cm^2/m^2 (range: 2.4–6.9 cm^2/m^2). Sarcopenia was associated with higher odds of overt HE (pooled odds ration [OR] = 3.40, 95% CI 1.85–6.25, p <0.001) and a higher proportion of overt HE (43%, 95% CI 26–61%) than in patients without sarcopenia (12%, 95% CI 7–20%). The proportionate mortality after TIPS was 18.3% (95% CI 14.6–22.8%), across 6–33.6 months of follow-up. The association between sarcopenia and mortality was not significant (HR: 1.95, 95% CI 0.89–4.31, p = 0.078).

Conclusions: In patients with cirrhosis and sarcopenia, TIPS is often followed by an improvement in sarcopenia. Sarcopenia is associated with higher odds of overt HE, whereas the effect of sarcopenia on mortality after TIPS remains uncertain.

© 2025 The Authors. Published by Elsevier B.V. on behalf of European Association for the Study of the Liver (EASL). This is an open access article under the CC BY license (http://creativecommons.org/licenses/by/4.0/).

Introduction

Transjugular intrahepatic portosystemic shunt (TIPS) is an established procedure for managing severe complications of portal hypertension, such as variceal bleeding and refractory ascites.[1–4] Sarcopenia is a prevalent and debilitating condition in cirrhosis, with an estimated prevalence of 40–70%.[5–7] Sarcopenia is characterized by progressive loss of skeletal muscle mass and function, which is quantified using imaging. Computed tomography (CT) at the third lumbar vertebra (L3) is a well-validated method, correlating with whole-body muscle mass.[6,7] This approach measures the cross-sectional area of major muscle groups (*e.g.* psoas, erector spinae, and abdominal muscles) at L3. This area is adjusted for patient height to obtain the L3-Skeletal Muscle Index (L3-SMI, cm^2/m^2), although cut-off values vary by sex, age, and study population. A simpler surrogate marker is the L3-Psoas Muscle Index (PMI), the psoas muscle area at L3 normalized for height (cm^2/m^2).[8]

Sarcopenia is associated with increased morbidity and mortality in patients with cirrhosis, before and after liver transplantation.[9] A recent study identified sarcopenia and portal hypertension as key risk factors for decompensation, ascites, and mortality in patients with cirrhosis,[10] while a meta-analysis reported a 2-fold higher mortality in patients with cirrhosis and sarcopenia.[11] In patients with cirrhosis undergoing TIPS, sarcopenia has been associated with a higher risk of hepatic encephalopathy (HE) and increased mortality.[5,12,13] However, emerging data suggest that TIPS improves sarcopenia, with contrasting effects on overt HE and mortality.[14,15]

Thus, we performed a systematic review and meta-analysis to evaluate: (1) improvement in sarcopenia after TIPS; (2) the proportion of patients developing overt HE after TIPS and its association with sarcopenia; and (3) the association between sarcopenia and mortality after TIPS.

Patients and methods

Protocol registration

This systematic review is reported according to the Preferred Reporting Items for Systematic Reviews and Meta-analyses

* Corresponding author. Address: Hepatology, University Clinic for Visceral Surgery and Medicine, Bern University Hospital, University of Bern, MEM F808, Murtenstrasse 35, CH-3008 Bern, Switzerland. Tel.: +41-31-632-30-26.
E-mail address: maria.debritorodriguesnunes@unibe.ch (M. de Brito Nunes).
† These authors contributed equally to this manuscript.
https://doi.org/10.1016/j.jhepr.2025.101699

(PRISMA) recommendations.[16] The PROSPERO registered protocol number is CRD42025646782.

Selection criteria and search strategy

We included peer-reviewed randomized controlled trials, cohort studies, case–control, cross-sectional studies and case series studies with peer-review. Eligible studies were those that had enrolled patients with cirrhosis who underwent TIPS and had sarcopenia assessed by CT using L3-SMI or L3-PMI.

Primary outcomes in patients with cirrhosis included improvement in sarcopenia after TIPS, the proportion developing overt HE after TIPS and its association with sarcopenia, and the association between sarcopenia and mortality after TIPS.

Sarcopenia was assessed by CT in accordance with the EASL Nutrition Guideline[17] and AASLD Guidance on Malnutrition and Sarcopenia.[18] We excluded studies using magnetic resonance imaging (MRI) because of limited standardization, a lack of validated reference values in cirrhosis, and poorer comparability between studies. The cross-sectional area of skeletal muscle at the L3 level (cm^2) was measured and normalized to patient height to calculate either L3-SMI or L3-PMI.[17]

Improvement in sarcopenia was defined as the transition from sarcopenia to non-sarcopenia, or an increase of at least 10% in L3-SMI or L3-PMI. Estimates were considered statistically significant if the 95% CI excluded the null value. Clinical significance based on functional measures (muscle strength/ performance) was not analyzable because these outcomes were seldom reported. Overt HE was defined as grade II or higher according to the West Haven criteria.[19]

We searched electronic databases from inception to January 31, 2025, with no language restrictions. Text words related to the research question and medical subject headings (MeSH) were used to search in Google Scholar, Medline (OVID interface), Embase (via OVIDSP), PubMed, Cochrane Library, Cochrane Central Register of Controlled Trials (CENTRAL), clinicaltrials.gov, EU Clinical Trials, and citations therein. Full search strategies are provided in Table S1.

Data extraction and quality assessment

Two researchers (MBN and MGD) independently screened titles, abstracts, and full texts for eligibility. Disagreements were resolved by discussion with two additional reviewers (AB and JB). Studies excluded in the full-text analysis were recorded, accompanied by a justification. For studies with multiple publications, we extracted data from the article with the most complete dataset. Using a standardized form, two authors (MBN and MGD) extracted the following data: author/publication year; study design and main outcomes of the study; sample size; duration of follow-up; population characteristics; pre and post-TIPS nutritional supplementation; indication of TIPS; cases of sarcopenia defined by L3-SMI or L3-PMI before TIPS; cases of sarcopenia defined by L3-SMI or L3-PMI after TIPS; cases of overt HE after TIPS; mortality after TIPS; and causes of death.

The risk of bias of each study was assessed by MBN and MGD using the Newcastle-Ottawa Scale (NOS) for cohort studies (Table S2). Studies were classified as high quality (*i.e.* low risk of bias) if ≥7 points, moderate quality if 5 or 6 points, and low quality if ≤4 points (*i.e.* high risk of bias). Twelve studies were classified as high quality,[15,20-30] seven studies were as of moderate quality,[14,29,31–35] and one study was of low quality.[36]

Data analysis

We calculated the odds ratio (OR) with 95% CI to assess the association between sarcopenia and overt HE after TIPS. If studies did not directly report ORs, these were estimated from available event counts and sample sizes. For mortality, we extracted hazard ratios (HRs) with 95% CIs from the original studies and the total number of deaths during the follow-up.

We summarized improvement in sarcopenia, proportion of overt HE, and mortality proportion after TIPS as pooled proportions with 95% CIs. Changes in L3-SMI (cm^2/m^2) were visualized using bar plots showing mean L3-SMI improvements across studies.

Given the expected clinical and methodological heterogeneity, we used random-effects models for all primary analyses and common-effect (fixed-effect) models as sensitivity analyses. We quantified heterogeneity with I^2 and its p value (Cochran's Q). We considered heterogeneity to be substantial when $I^2 \geq 50\%$ or $p \leq 0.10$. If at least 10 studies were available, we explored heterogeneity via subgroup analyses (categorical modifiers) and meta-regression (continuous modifiers). Candidate effect modifiers included age, L3-SMI/L3-PMI cut-offs, TIPS indication, portal pressure gradient, liver disease severity (model of end-stage liver disease score [MELD] score), and follow-up duration after TIPS.

Publication bias was assessed using funnel plots, if ≥10 studies were available. We inspected funnel plot asymmetry visually and, if appropriate, applied Egger's regression test. The quality of evidence was evaluated using the GRADE framework.[37] Details are provided in Table S3. Statistics analyses were performed using R (version 4.2.3, R Foundation for Statistical Computing, Vienna, Austria).

Results

We identified 346 records after database searches, with three additional records from trial registries. After removing 238 duplicated records, 111 records were screened. We analyzed 50 full-text articles, of which 20 met the inclusion criteria (Fig. 1).

After excluding one study with high risk of bias,[36] 19 studies were included in the meta-analysis: two were prospective observational studies[20,28] and 17 were retrospective observational studies.[14,21–27,29–35,38,39] The indication for TIPS was refractory ascites and variceal bleeding in 17 studies,[14,21,22,24,25,27–30,32–35,39] variceal bleeding alone in two studies,[23,39] and refractory ascites alone in one study.[26] The included studies applied different cut-offs values to assess sarcopenia, based on L3-SMI or L3-PMI measured by CT (Table 1).

Information on concomitant nutritional supplementation or physical rehabilitation during follow-up was not reported. Baseline characteristics of the studies are summarized in Table S4.

Improvement in sarcopenia after TIPS

Ten studies involving a total of 1,008 patients (596 with sarcopenia criteria) evaluated improvement in sarcopenia after

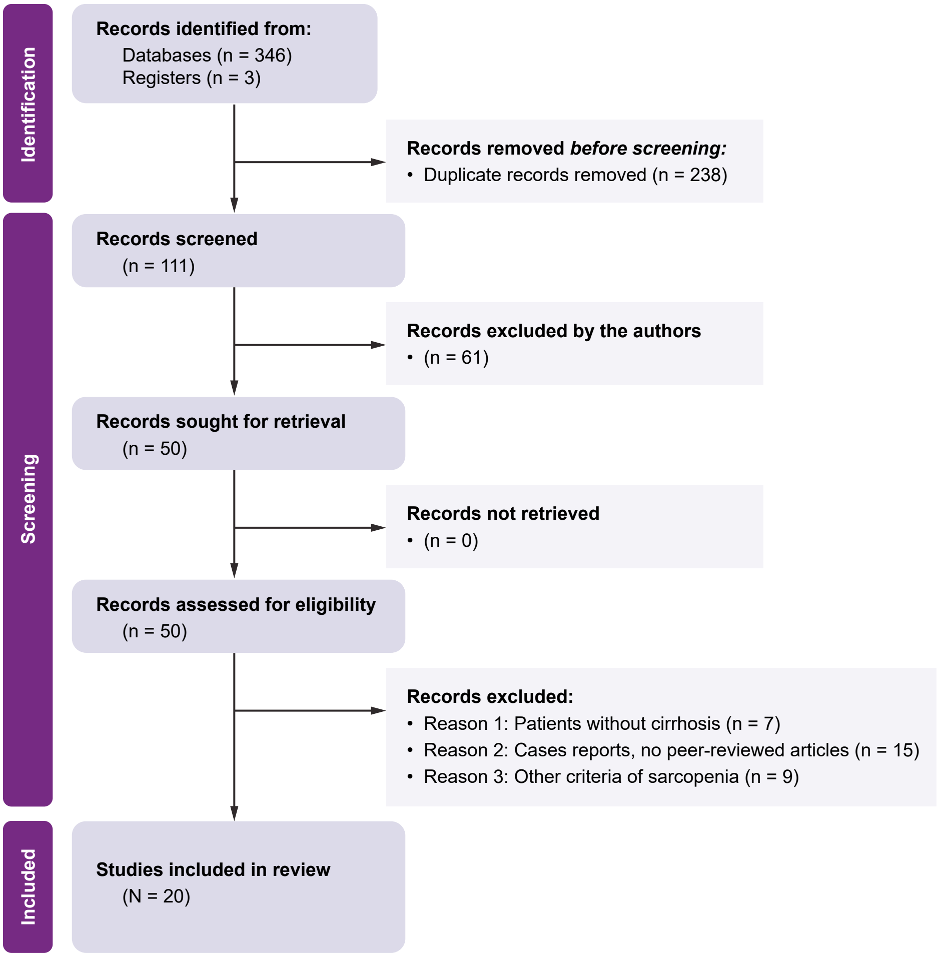

Fig. 1. PRISMA flow diagram of the systematic literature search process. PRISMA, Preferred Reporting Items for Systematic Reviews and Meta-analyses.

Table 1. Cut-off values for defining sarcopenia used by included studies.

Sarcopenia definition	Female (cm^2/m^2)	Male (cm^2/m^2)	Studies
EASL/AASLD (L3-SMI)	<39	<50	11 studies[14,21,22,25–27,30,32,35]
Chinese specific (L3-SMI)	<32.50	<44.77	2 studies[15,31]
JSH (L3-SMI)	<38	<42	1 study[33]
Alternative cut-offs (L3-SMI)	<39.5	<52	1 study[34]
	<38.5	<50	1 study[20]
	<38.5	<52.4	1 study[28]
	<41	<53	1 study[24]
L3-PMI	<3.2	<4.4	1 study[29]

JSH, Japanese Society of Hepatology; L3-PMI, psoas muscle index at the third lumbar vertebra; L3-SMI, skeletal muscle index at the third lumbar vertebra.

TIPS, using L3-SMI or L3-PMI measurements. The baseline prevalence of sarcopenia in patients with cirrhosis undergoing TIPS, assessed in eight studies,[20,23,25–27,33–35] was 55.4% (95% CI: 41.7–68.2%). Two studies[14,21] including only patients with sarcopenia were excluded from this prevalence estimate. The corresponding forest plot is shown in Fig. S1.

The pooled proportion with improvement in sarcopenia after TIPS was 57% (95% CI 48–65) using a random-effects model. A common-effect (fixed-effect) analysis provided similar results (Fig. 2). Heterogeneity was substantial (I^2 = 68.5%, p <0.0008). Funnel plot asymmetry was suggested visually (Fig. S2) but not confirmed by Egger's test (z = 1.23, p = 0.22). Subgroup analysis by follow-up (≤6 months, >6–12 months, and >12 months) showed no significant differences (p = 0.86; Fig. S3). The median L3-SMI improvement after TIPS in patients with sarcopenia was 4.53 cm^2/m^2 (range: 2.4–6.9 cm^2/m^2) across seven studies (Fig. 3).

Other nonstudied factors, such as having concomitant nutritional supplementation, physical rehabilitation, severity of liver disease, or L3-SMI or L3-PMI cut-offs, could influence this outcome. Subgroup analysis by liver disease severity (MELD score) could not be performed, because separate MELD

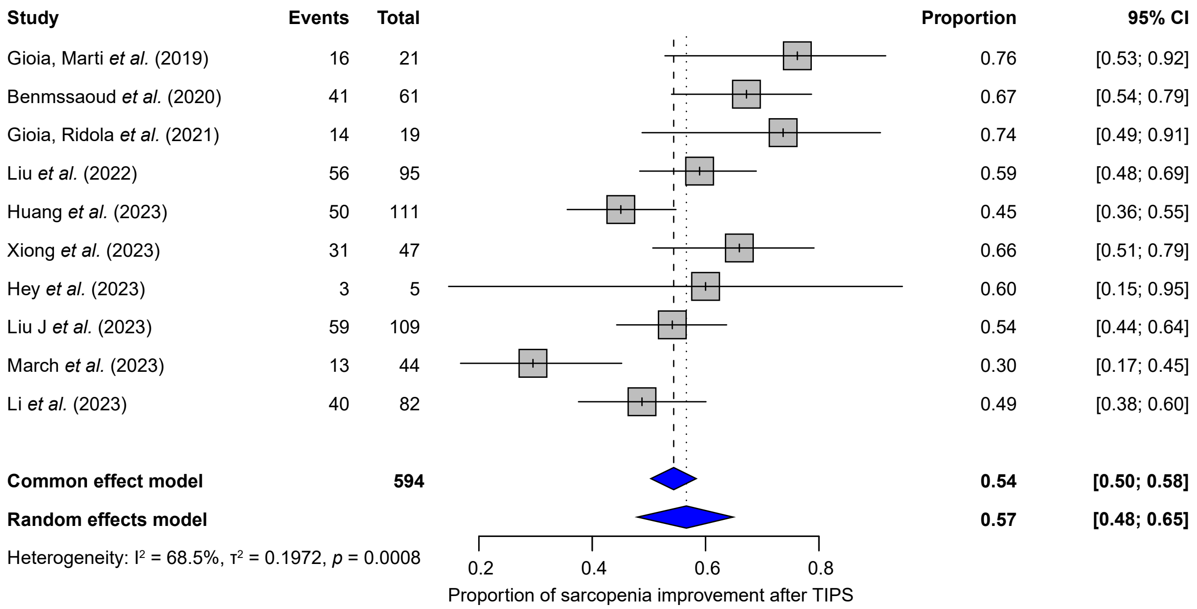

Fig. 2. Proportion of patients with improvement of sarcopenia and 95% CI in patients with sarcopenia undergoing TIPS. TIPS, transjugular intrahepatic portosystemic shunt. Between-study heterogeneity was assessed with I^2 and Cochran's Q test (*p* value).

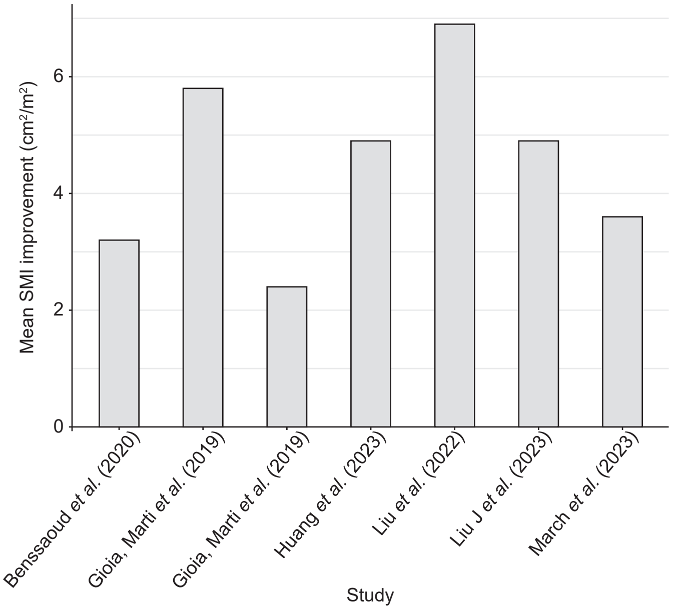

Fig. 3. Mean L3-SMI improvement by study. The pooled mean change was estimated separately using a random-effects meta-analysis. L3-SMI, skeletal muscle index at the third lumbar vertebra.

scores for patients with and without sarcopenia were unavailable. Subgroup analysis by sarcopenia cut-offs was not performed because of limited statistical power, given that some cut-offs appeared in only one or two studies.

Overt hepatic encephalopathy after TIPS

Eleven studies,[20,22,23,25–28,30–32,35] including 1,839 patients (1,080 with sarcopenia), reported the proportion of overt HE after TIPS. The pooled proportion of overt HE after TIPS was 31.5% (95% CI 23.5–40.6%), as estimated by the random-effects model. A common-effect (fixed-effect) analysis gave similar results (30.8%, 95% CI 28.7–32.9), with high heterogeneity (I^2 = 85.7%, *p* <0.01; Fig. S4).

The proportion of overt HE after TIPS was higher in patients with sarcopenia (43%, 95% CI 26–61%) than in patients without sarcopenia (12%, 95% CI 7–20%). The random-effects model confirmed a statistically significant difference (χ^2 = 11.72, degrees of freedom = 1, *p* <0.01). Substantial heterogeneity was observed in the group with sarcopenia (I^2 = 82%, *p* <0.01), whereas it was low in the group without sarcopenia (I^2 = 18.4%, *p* = 0.14), suggesting consistency among studies (Fig. 4).

Visual inspection suggested funnel plot asymmetry (Fig. S5), but Egger's test did not indicate small-study effects (*p* = 0.51). Observed proportions of 0.00 in several small studies[20,25,27,28] reflected no overt HE events in one of the subgroups.

Sarcopenia was associated with higher odds of developing overt HE after TIPS (pooled OR, 3.40; 95% CI 1.85–6.25; *p* <0.001; Fig. 5). A continuity correction of 0.5 was applied to handle zero HE events in the group without sarcopenia, especially seen in studies with low samples sizes. Three individual studies reported associations consistent with this finding. Kapoor *et al.*[22] reported higher odds of overt HE after TIPS in patients with sarcopenia (OR = 2.8, 95% CI 1.08–7.4, *p* = 0.02). On univariate logistic regression model, low L3-SMI was a risk factor for overt HE after TIPS (OR = 0.94, 95% CI 0.89–0.99, *p* = 0.03). Nardelli *et al.*[28] reported an increased risk of overt HE after TIPS associated with sarcopenia on multivariate competing risk regression analysis (subdistribution HR = 31.3, 95% CI 4.5–218.07, *p* <0.001). Benmassaoud *et al.*[26] found an HR of 0.95 (95% CI 0.91–0.99, *p* = 0.01) on multivariate Cox regression analysis, indicating that each

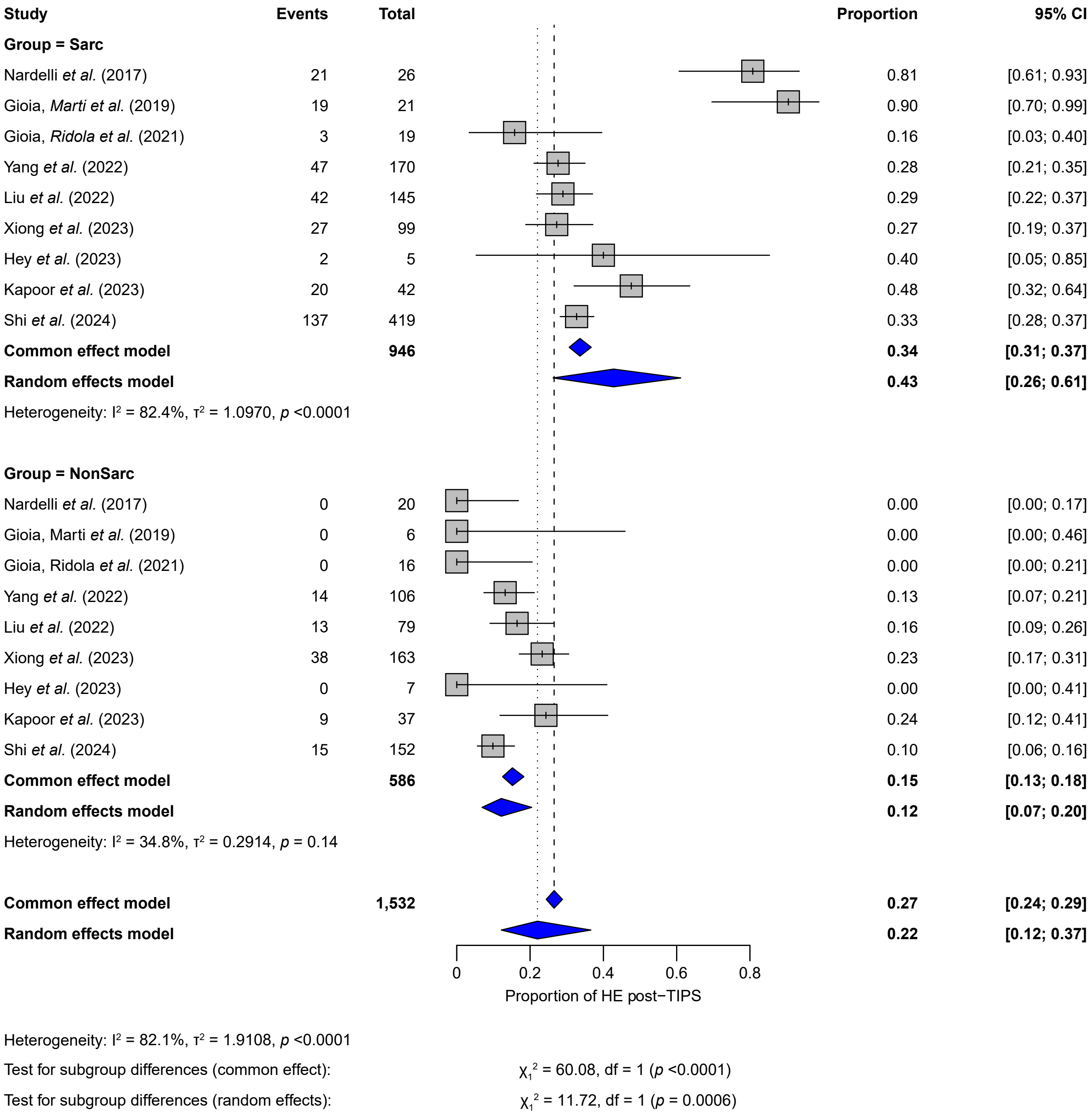

Fig. 4. Subgroup analysis comparing the proportion of HE after TIPS between patients with sarcopenia (upper panel) and without sarcopenia (lower panel). Proportions with 95% CIs are shown for each study. Between-study heterogeneity was assessed with I^2 and Cochran's Q test (*p* value). HE, Hepatic encephalopathy; TIPS, transjugular intrahepatic portosystemic shunt.

incremental increase (per unit) in L3-SMI reduced the risk of developing overt HE, and higher L3-SMI was protective against HE. Only Wang *et al.*[31] reported a nonsignificant association of sarcopenia measured by L3-SMI and overt HE after TIPS in either male patients (OR = 0.94, 95% CI 0.87–1.02, $p = 0.142$) or female patients (OR = 0.95, 95% CI 0.87–1.04, $p = 0.272$).

A subgroup analysis (Fig. S6) comparing studies reporting previous HE (n = 7) *vs.* those not reporting it (n = 4) found no differences in the proportion of overt HE after TIPS (incidence 28.7% *vs.* 31.7%). Heterogeneity remained substantial (I^2 = 84.5%). The test for subgroup differences was nonsignificant under either the common-effect ($p = 0.19$) or random-effects model ($p = 0.94$). In meta-regression (Fig. S7), follow-up time was not associated with the proportion of overt HE after TIPS ($p = 0.454$) and did not explain heterogeneity (R^2 = 0.00%). Given limited available data, we could not assess whether patients with sarcopenia had a higher prevalence of overt HE before TIPS compared with patients without sarcopenia. Subgroup analysis based on liver disease severity, L3-SMI/L3-PMI cut-offs, age, or portal pressure gradient were not performed, for the same reasons.

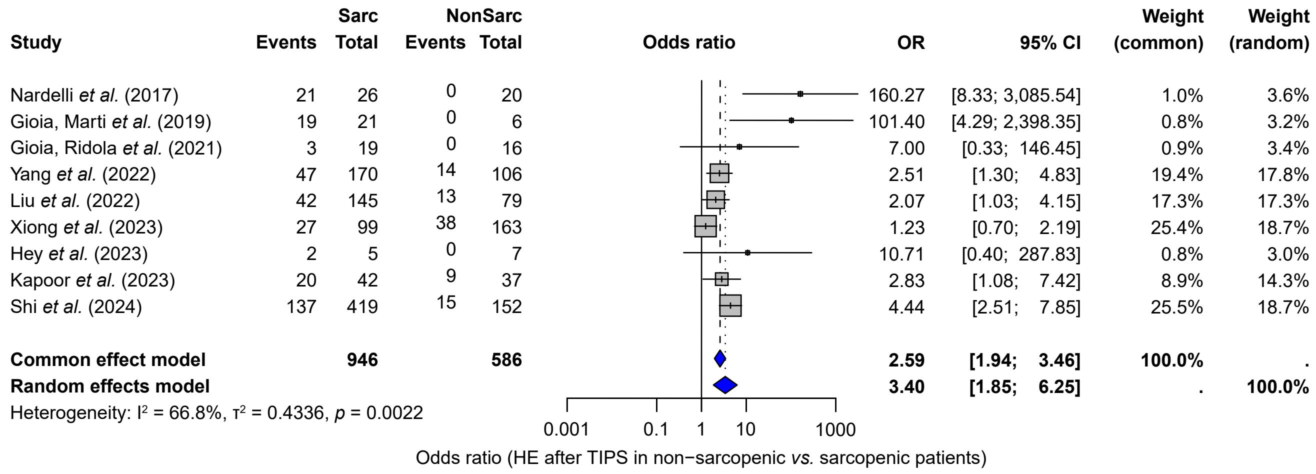

Fig. 5. ORs for the development of overt HE after TIPS in patients with and without sarcopenia. ORs with 95% CIs are shown for each study. Between-study heterogeneity was assessed with I^2 and Cochran's Q test (*p* value). HE, Hepatic encephalopathy; OR, odds ratio; TIPS, transjugular intrahepatic portosystemic shunt.

Mortality after TIPS

Eight studies[20,23,24,26,28,34,35,39] reported overall mortality after TIPS, including 1,194 patients and 211 deaths. The overall mortality proportion was 18.31% (95% CI 14.57–22.77%) according to a random-effects model. Comparable results were obtained considering a common-effect (fixed-effect) model (Fig. 6). The follow-up duration varied across studies, ranging from 6 to 33.6 months (median: 16.5 months).

The pooled HR from five studies comparing HRs for mortality after TIPS in patients with and without sarcopenia was 1.95 (95% CI 0.89–4.31, *p* = 0.0783), not reaching statistical significance, under a random-effects model (Fig. 7A). In the subset of studies reporting adjusted HRs, the pooled adjusted HR was 2.86 (95% CI 1.51–5.40, *p* = 0.5131; Fig. S9). A meta-analysis of unadjusted HRs for the same give studies also showed no significant association (HR = 2.04, 95% CI 0.84–4.83, *p* = 0.0828; Fig. S8). In a meta-regression pooling adjusted and unadjusted HRs, adjustment status did not influence effect size (HR = 1.12; 95% CI 0.69–1.84; *p* = 0.64; Fig. S10).

In a meta-analysis of four studies reporting HRs per unit increase in skeletal muscle indices (L3-SMI or L3-PMI), the pooled HR was 0.97 (95% CI 0.88–1.06, *p* = 0.34) under a random-effects model, indicating no statistically significant association (Fig. 7B). However, the common-effect (fixed-effect) model showed a significant association (HR = 0.97, 95% CI 0.95–0.99, *p* = 0.013), reporting a 3% reduction in mortality risk per cm^2/m^2 increase in muscle mass. Heterogeneity was substantial (I^2 = 71.7%, *p* = 0.014). All HRs in this analysis were derived from univariate Cox regression models. Given that only

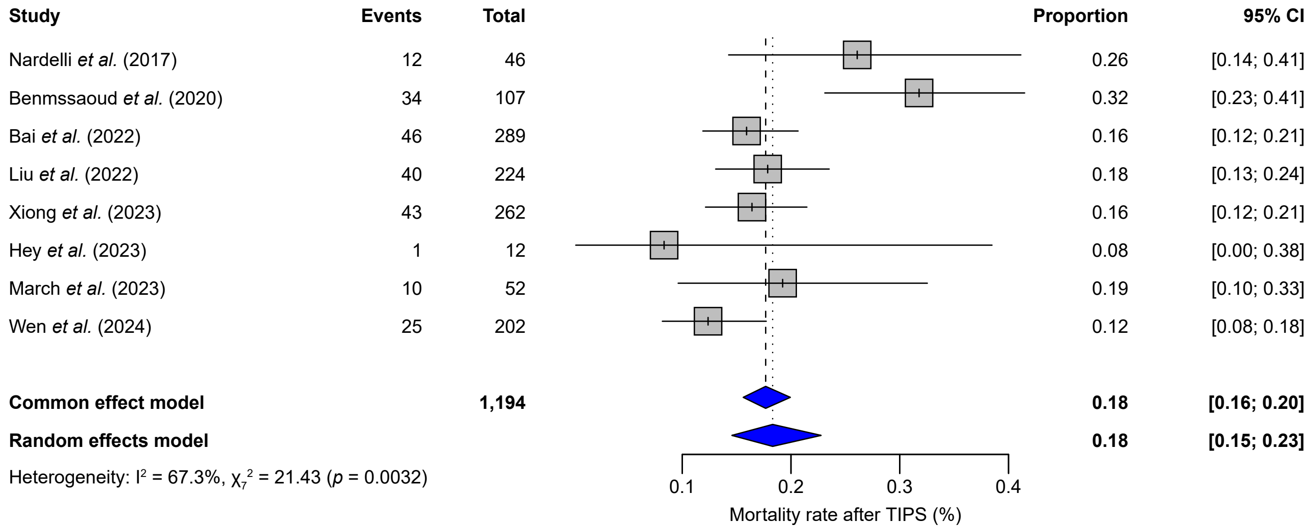

Fig. 6. Overall proportionate mortality after TIPS in patients with cirrhosis. Proportions with 95% CIs are shown for each study. Between-study heterogeneity was assessed with I^2 and Cochran's Q test (p value). Mortality was reported at the last available follow-up, which ranged from 6 to 33.6 months across studies. TIPS, transjugular intrahepatic portosystemic shunt.

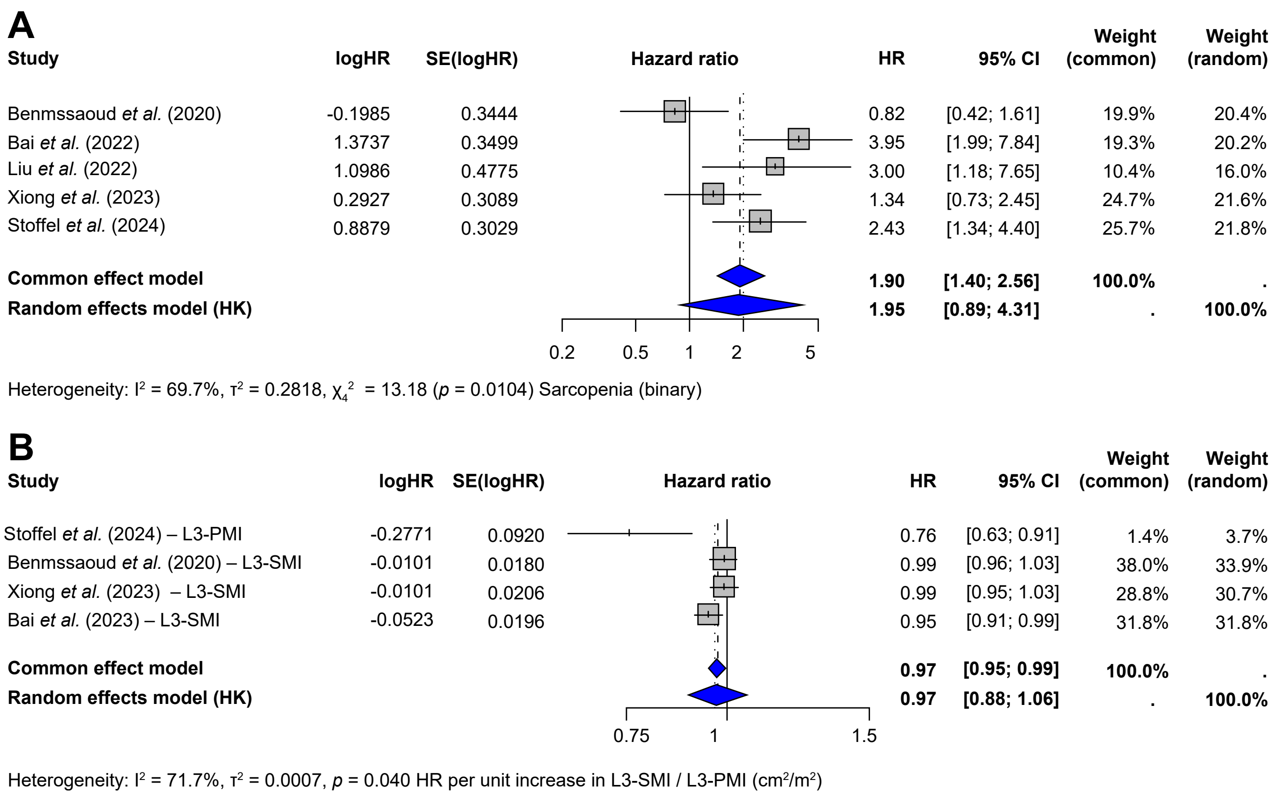

Fig. 7. Association between sarcopenia and mortality after TIPS. (A) HRs for mortality comparing patients with and without sarcopenia (binary variable), regardless of adjustment status (univariate and multivariate Cox regression model). (B) HRs per unit increase in L3-SMI/L3-PMI (continuous variable), calculated by univariate cox regression model. HRs with 95% CIs are shown for each study. Between-study heterogeneity was assessed with I^2 and Cochran's Q test (*p* value). HR, hazard ratio; L3-PMI, psoas muscle index at the third lumbar vertebra; L3-SMI, skeletal muscle index at the third lumbar vertebra; TIPS, transjugular intrahepatic portosystemic shunt.

five studies reported HRs for mortality after TIPS, subgroup analyses or meta-regression by L3-SMI/L3-PMI cut-offs, liver disease severity (MELD), or follow-up duration after TIPS were not feasible.

Discussion

This study provides a comprehensive summary of outcomes in patients with cirrhosis and sarcopenia undergoing TIPS. The pooled prevalence of sarcopenia in patients undergoing TIPS was 55.4% (95% CI 41.7–68.2%). Among 596 patients with sarcopenia, 57% (95% CI 48–65%) demonstrated an improvement in sarcopenia after TIPS, with a mean L3-SMI increase of 4.53 cm^2/m^2. The pooled proportion of overt HE after TIPS was 31.5% (95% CI 23.5–40.6%). This proportion was higher in patients with sarcopenia (43%) than in those without sarcopenia (12%) ($p < 0.01$). Sarcopenia was associated with higher odds of overt HE after TIPS (pooled OR, 3.40, $p < 0.001$). The overall mortality proportion after TIPS was 18.3% (95% CI 14.6–22.8%). There was no significant association between sarcopenia and mortality (HR = 1.64, 95% CI 0.91–2.98, $p = 0.087$).

Half of patients with baseline sarcopenia experienced improvement in sarcopenia after TIPS. This aligns with the systematic review by Gădža *et al.*,[40] which reported improvements in ascites-free body weight, BMI, or body mass area in 10 studies evaluating nutritional outcomes after TIPS. Artru *et al.*.[41] also observed changes in body composition after TIPS using transversal psoas thickness/height (TPMT/height), a surrogate marker of sarcopenia. Their findings are consistent with ours, but differences in methodology precluded inclusion in our pooled analysis. TIPS reduces portal hypertension, resulting in decreased intestinal congestion and likely improved absorption of nutrients. This is supported by the observation that TIPS can decrease bacterial translocation and associated systemic inflammation.[42,43] Recent studies showed that TIPS placement leads to a significant reduction in proinflammatory cytokines, particularly IL-6, which has a central role in the inflammatory response in cirrhosis.[42] By contrast, elevated levels of IL-8 predicted worse clinical outcomes after TIPS.[44] The degree of improvement in sarcopenia might still depend on baseline liver function, nutritional status and supplementation, physical activity, and length of follow-up after TIPS, factors that were not reported in the included studies.

The pooled OR for overt HE after TIPS in patients with sarcopenia was 3.40 ($p < 0.001$), indicating ~3-fold higher odds of overt HE after TIPS. Our findings align with those of Ahmed *et al.*,[45] who reported a pooled risk ratio of 1.68 (95% CI 1.48–1.91; $p < 0.004$), identifying sarcopenia as a risk factor of HE after TIPS. This study included heterogeneous definitions of sarcopenia, whereas our analysis focused on L3-SMI or L3-PMI measured by CT. The stronger association observed in

our study could be attributed to this stricter imaging-based classification and the higher methodological homogeneity in how sarcopenia was measured. These findings support pre-procedural assessment of sarcopenia as a key element in HE risk stratification. The pathophysiological mechanisms underlying this association are well described. First, sarcopenia reduces ammonia detoxification capacity, because skeletal muscle is an extrahepatic site for ammonia metabolism and conversion to glutamine.[46] The reduction in extrahepatic ammonia metabolism contributes to the accumulation of neurotoxins after TIPS placement, increasing the risk of HE. Second, hyperammonemia accelerates muscle wasting by inducing mitochondrial dysfunction, cellular stress, and transcriptional upregulation of myostatin.[47,48] Hyperammonemia is an important negative regulator of protein synthesis and cell proliferation and differentiation.[46,48]

In our meta-analysis, only three studies[25,27,30] specified that patients did not receive HE prophylaxis before TIPS, even though HE prophylaxis can reduce overt HE after TIPS. A double-blind, placebo-controlled, randomized, multicenter trial showed that rifaximin, started 2 weeks before TIPS placement and continued for at least 6 months, reduced the incidence of overt HE after TIPS by ~50%.[49] A recent meta-analysis comparing the effectiveness of rifaximin, lactulose, lactitol, L-ornithine-L-aspartate (LOLA), albumin, and combinations reported that rifaximin plus lactulose was associated with fewer HE events after TIPS.[50] EASL Clinical Practice Guidelines on TIPS[51] recommend using lactulose to treat and prevent the recurrence of HE, and rifaximin can be added if lactulose is ineffective or not tolerated. The BAVENO VII consensus[1] suggests considering rifaximin for preventing HE in patients with a history of HE undergoing elective TIPS.

A previous meta-analysis by Ahmed *et al.*[45] reported an association (relative risk: 1.70, 95% CI 1.13–1.54, p <0.001) between sarcopenia and mortality after TIPS, but our results do not support those findings. Another meta-analysis by Dajti *et al.*,[11] focusing on patients with cirrhosis (regardless of whether they underwent TIPS), showed that sarcopenia defined by EASL/AASLD CT-based criteria is an independent predictor of mortality. This discrepancy could result from the inclusion in our meta-analysis of two studies, by Benmassaoud *et al.*[26] and Xiong *et al.*,[15] reporting no association between sarcopenia and mortality after TIPS. In addition, the improvement in sarcopenia observed in 57% of patients after TIPS in our study could attenuate the adverse prognostic effects of sarcopenia.

The main limitation of our study is the substantial heterogeneity observed across analyses. Although random-effects models were used, our ability to explore the sources of heterogeneity was limited. Most studies did not report information separately for patients with and without sarcopenia, which restricts subgroup analyses. Key effect modifiers, such as concomitant nutritional supplementation, physical rehabilitation, prophylactic use of pharmacological treatment to prevent HE (rifaximin or lactulose), or prevalence of HE stratified by sarcopenia were not systematically reported, even though these factors can influence the outcomes after TIPS. Even with a uniform imaging approach (L3-SMI/L3-PMI measured by CT), heterogeneity persisted, in part, because of different cut-off values for sarcopenia that reflect ethnic differences in body composition. An individual patient data meta-analysis would allow harmonized sarcopenia definitions, adjustment for key confounders, and subgroup analyses by MELD, age, and portal pressure gradient. This was not feasible because of the lack of access to individual patient data. The use of CT imaging enhances internal consistency, but it can limit extrapolation to settings that estimate sarcopenia with other methods (*e.g.* bioelectrical impedance analysis, dual-energy X-ray absorptiometry, or MRI).

This study has important strengths. All included studies assessed sarcopenia using L3-SMI or L3-PMI measured by CT, which is recognized as a reliable method to quantify muscle mass. The meta-analysis was conducted following rigorous methodological practice, including duplicate screening, data extraction, assessment of risk of bias using the NOS, and adherence to PRISMA reporting standards. Finally, this systematic review and meta-analysis evaluated the three key outcomes after TIPS (improvement in sarcopenia, overt HE, and mortality) allowing a balanced appraisal of both benefits and risks in patients with cirrhosis and sarcopenia.

Our results show that sarcopenia is associated with higher odds of overt HE after TIPS, whereas TIPS is followed by improvement in sarcopenia in approximately half of patients. This dual effect challenges the view that TIPS is linked only to adverse outcomes in cirrhosis with sarcopenia, suggesting a potential benefit through improvement in sarcopenia (increase in muscle mass indices). Caution is warranted in generalizing these findings to settings where sarcopenia is assessed using methods other than L3-SMI or L3-PMI measured by CT.

Prospective studies are needed to confirm whether patients who experienced improvement in sarcopenia after TIPS are protected from the elevated risk of overt HE and mortality. Identifying predictive factors of improvement in sarcopenia after TIPS is crucial for accurate risk stratification and patient management. Future studies should incorporate standardized sarcopenia definitions; control for confounders, such as nutritional status and rehabilitation, or administration of prophylactic medication for overt HE; and report longitudinal changes in muscle mass and clinical outcomes.

In summary, patients with cirrhosis and sarcopenia undergoing TIPS have higher odds of overt HE, whereas TIPS is also followed by improvement in sarcopenia in approximately half of patients. Recognizing this dual effect is important for clinical decision-making. However, the association between sarcopenia and mortality after TIPS remains uncertain. Our findings support the importance of individualized risk assessment, careful patient selection, and post-procedural monitoring. Sarcopenia and malnutrition should be systematically evaluated with a nutritional assessment before TIPS to guide both prognosis and follow-up strategies.

Affiliations

[1]*Department of Visceral Surgery and Medicine, Inselspital, Bern University Hospital, University of Bern, Switzerland;* [2]*Department of Internal Medicine, Hospital of Fribourg, Switzerland;* [3]*Graduate School for Health Sciences (GHS), University of Bern, Bern, Switzerland*

Abbreviations

CT, computed tomography; HE, hepatic encephalopathy; HR, hazard ratio; JSH, Japanese Society of Hepatology; L3-PMI, Psoas muscle index at the third lumbar vertebra; L3-SMI, skeletal muscle index at the third lumbar vertebra; LOLA, L-ornithine-L-aspartate; MELD, model for end-stage liver disease; MeSH, Medical subject headings; MRI, magnetic resonance imaging; NOS, Newcastle–Ottawa Scale; OR, odds ratio; PRISMA, Preferred Reporting Items for Systematic Reviews and Meta-analyses; TIPS, Transjugular intrahepatic portosystemic shunt; TPMT, transversal psoas thickness.

Financial support

MBN was supported by the Swiss Foundation for Liver Diseases.

Conflicts of interest

The authors have no conflicts of interest to report.
Please refer to the accompanying ICMJE disclosure forms for further details.

Authors' contributions

Conceptualization: MBN, MGD, AB. Methodology: MBN, AB. Investigation: MGD. Data curation: MBN, MGD. Formal analysis: MBN, JB. Validation: JB. Writing (original draft): MBN, MGD. Writing – review & editing: JB, AB. Critical review for intellectual content: JB, AB. Supervision: AB.

Data availability

All data underlying this study are included in the article and its supplementary materials. Additional extraction tables are available from the corresponding author upon reasonable request.

Acknowledgements

The authors thank Jonas Schropp for his advice regarding the statistical analysis of mortality.

Declaration of generative AI and AI-assisted technologies in the writing process

During the preparation of this work, the authors used language-editing tools, including ChatGPT and Grammarly, to improve spelling and syntax. After using this tool/service, the authors reviewed and edited the content as needed and take full responsibility for the content of the publication.

Supplementary data

Supplementary data to this article can be found online at https://doi.org/10.1016/j.jhepr.2025.101699.

References

Author names in bold designate shared co-first authorship

[1] de Franchis R, Bosch J, Garcia-Tsao G, et al. Baveno VII – renewing consensus in portal hypertension. J Hepatol 2022;76:959–974.
[2] Bosch J. Small diameter shunts should lead to safe expansion of the use of TIPS. J Hepatol 2021;74:230–234.
[3] García-Pagán JC, Saffo S, Mandorfer M, et al. Where does TIPS fit in the management of patients with cirrhosis? JHEP Rep 2020;2:100122.
[4] Garcia-Tsao G, Abraldes JG, Berzigotti A, et al. Portal hypertensive bleeding in cirrhosis: risk stratification, diagnosis, and management: 2016 practice guidance by the American Association for the study of liver diseases. Hepatology 2017;65:310–335.
[5] Carey EJ, Lai JC, Wang CW, et al. A multicenter study to define sarcopenia in patients with end-stage liver disease. Liver Transpl 2017;23:625–633.
[6] Cruz-Jentoft AJ, Bahat G, Bauer J, et al. Sarcopenia: revised European consensus on definition and diagnosis. Age Ageing 2019;48:16–31.
[7] Ebadi M, Bhanji RA, Mazurak VC, et al. Sarcopenia in cirrhosis: from pathogenesis to interventions. J Gastroenterol 2019;54:845–859.
[8] Li T, Wang Z, Liu Y, et al. Psoas muscle index in sarcopenia following transjugular intrahepatic portosystemic shunt: a multicenter, retrospective study. Portal Hypertens Cirrhosis 2024;3:173–183.
[9] Kim G, Kang SH, Kim MY, et al. Prognostic value of sarcopenia in patients with liver cirrhosis: a systematic review and meta-analysis. PLoS ONE 2017;12:e0186990.
[10] Dajti E, Renzulli M, Ravaioli F, et al. The interplay between sarcopenia and portal hypertension predicts ascites and mortality in cirrhosis. Dig Liver Dis 2023;55:637–643.
[11] Dajti E, Rodrigues SG, Perazza F, et al. Sarcopenia evaluated by EASL/AASLD computed tomography-based criteria predicts mortality in patients with cirrhosis: a systematic review and meta-analysis. JHEP Rep 2024;6:101113.
[12] Delgado MG, Mertineit N, Bosch J, et al. Combination of Model for End-Stage Liver Disease (MELD) and sarcopenia predicts mortality after transjugular intrahepatic portosystemic shunt (TIPS). Dig Liver Dis 2024;56:1544–1550.
[13] Ronald J, Bozdogan E, Zaki IH, et al. Relative sarcopenia with excess adiposity predicts survival after transjugular intrahepatic portosystemic shunt creation. Am J Roentgenol 2020;214:200–205.
[14] **Huang S**, **Liu J**, Cai J, et al. Predictors of improvement of sarcopenia after transjugular intrahepatic portosystemic shunt creation in cirrhotic patients. J Vasc Interv Radiol 2023;34:639–644.
[15] Xiong B, Yang C, Zhu X, et al. The added value of sarcopenia on existing risk scores to predict mortality after tips placement: a multicenter study. Acad Radiol 2023;30(Suppl 1):S246–S256.
[16] Page MJ, Moher D, Bossuyt PM, et al. PRISMA 2020 explanation and elaboration: updated guidance and exemplars for reporting systematic reviews. BMJ 2021;372:n160.
[17] Merli M, Berzigotti A, Zelber-Sagi S, et al. EASL Clinical Practice Guidelines on nutrition in chronic liver disease. J Hepatol 2019;70:172–193.
[18] Lai JC, Tandon P, Bernal W, et al. Malnutrition, frailty, and sarcopenia in patients with cirrhosis: 2021 Practice Guidance by the American Association for the Study of liver diseases. Hepatology 2021;74:1611–1644.
[19] Montagnese S, Rautou P-E, Romero-Gómez M, et al. EASL Clinical Practice Guidelines on the management of hepatic encephalopathy. J Hepatol 2022;77:807–824.
[20] Hey P, Chapman B, Wong D, et al. Transjugular intrahepatic portosystemic shunt insertion improves muscle mass but not muscle function or frailty measures. Eur J Gastroenterol Hepatol 2023;35:997–1003.
[21] Liu J, Yang C, Yao J, et al. Improvement of sarcopenia is beneficial for prognosis in cirrhotic patients after TIPS placement. Dig Liver Dis 2023;55:918–925.
[22] Bhatia Kapoor P, Benjamin J, Tripathi H, et al. Post-transjugular intrahepatic portosystemic shunt hepatic encephalopathy: sarcopenia adds insult to injury. Turk J Gastroenterol 2023;34:406–412.
[23] Xiong B, Yang C, Zhou C, et al. TIPS placement as the first-line therapy to prevent variceal rebleeding in patients with cirrhosis and sarcopenia. Eur J Radiol 2023;158:110630.
[24] Bai Y, Liu J, Yang C, et al. Inclusion of sarcopenia improves the prognostic value of MELD score in patients after transjugular intrahepatic portosystemic shunt. Eur J Gastroenterol Hepatol 2022;34:948–955.
[25] Gioia S, Ridola L, Cristofaro L, et al. The improvement in body composition including subcutaneous and visceral fat reduces ammonia and hepatic encephalopathy after transjugular intrahepatic portosystemic shunt. Liver Int 2021;41:2965–2973.
[26] Benmassaoud A, Roccarina D, Arico F, et al. Sarcopenia does not worsen survival in patients with cirrhosis undergoing transjugular intrahepatic portosystemic shunt for refractory ascites. Am J Gastroenterol 2020;115:1911.
[27] Gioia S, Merli M, Nardelli S, et al. The modification of quantity and quality of muscle mass improves the cognitive impairment after TIPS. Liver Int 2019;39:871–877.
[28] Nardelli S, Lattanzi B, Torrisi S, et al. Sarcopenia is risk factor for development of hepatic encephalopathy after transjugular intrahepatic portosystemic shunt placement. Clin Gastroenterol Hepatol 2017;15:934–936.
[29] Stoffel E, Hwang SY, Qian X, et al. Sarcopenia is an independent risk factor for short-term mortality in patients undergoing transjugular intrahepatic portosystemic shunt. Eur J Gastroenterol Hepatol 2024;36:1010–1015.
[30] Shi W, Xu W, Fan N, et al. Body compositions correlate with overt hepatic encephalopathy after transjugular intrahepatic portosystemic shunt: a multicentre cohort study. J Clin Gastroenterol 2025;59:262–268.
[31] Wang C, Teng Y, Gao J, et al. Low adipose tissue index as an indicator of hepatic encephalopathy in cirrhotic patients following transjugular intrahepatic portosystemic shunt. Abdom Radiol 2023;48:1454–1467.

[32] Yang C, Zhu X, Liu J, et al. Development and validation of prognostic models to estimate the risk of overt hepatic encephalopathy after TIPS creation: a multicenter study. Clin Transl Gastroenterol 2022;13:e00461.
[33] Li T, Liu J, Zhao J, et al. Sarcopenia defined by psoas muscle thickness predicts mortality after transjugular intrahepatic portosystemic shunt. Dig Dis Sci 2023;68:1641–1652.
[34] March C, Thormann M, Geipel S, et al. Increase of radiologically determined muscle area in patients with liver cirrhosis after transjugular intrahepatic portosystemic shunt. Sci Rep 2023;13:17092.
[35] Liu J, Ma J, Yang C, et al. Sarcopenia in patients with cirrhosis after transjugular intrahepatic portosystemic shunt placement. Radiology 2022;303:711–719.
[36] Wu C-H, Ho M-C, Kao J-H, et al. Effects of transjugular intrahepatic portosystemic shunt on abdominal muscle mass in patients with decompensated cirrhosis. J Formos Med Assoc 2023;122:747–756.
[37] Balshem H, Helfand M, Schünemann HJ, et al. GRADE guidelines: 3. Rating the quality of evidence. J Clin Epidemiol 2011;64:401–406.
[38] Vanderschueren E, Meersseman P, Wilmer A, et al. Sarcopenia in patients receiving TIPS is independently associated with increased risk of complications and mortality. Dig Liver Dis 2025;57:549–557.
[39] Wen Z, Tuo S, Liu Y, et al. Validating the prognostic value of muscle changes in patients with cirrhosis undergoing transjugular intrahepatic portosystemic shunt. Hepatobiliary Surg Nutr 2024;13:1010–1014.
[40] Gazda J, Di Cola S, Lapenna L, et al. The impact of transjugular intrahepatic portosystemic shunt on nutrition in liver cirrhosis patients: a systematic review. Nutrients 2023;15:1617.
[41] Artru F, Miquet X, Azahaf M, et al. Consequences of TIPSS placement on the body composition of patients with cirrhosis and severe portal hypertension: a large retrospective CT-based surveillance. Aliment Pharmacol Ther 2020;52:1516–1526.
[42] Kornfehl A, Tiede A, Hemetsberger P, et al. Decreasing interleukin-6 levels after TIPS predict outcomes in decompensated cirrhosis. JHEP Rep 2025;7:101308.
[43] Tiede A, Maasoumy B. TIPS insertion and systemic inflammation: is it ever too late to lower portal pressure? Correspondence to editorial on "Insertion of a transjugular intrahepatic portosystemic shunt leads to sustained reversal of systemic inflammation in patients with decompensated liver cirrhosis". Clin Mol Hepatol 2025;31:e176–e179.
[44] Liu G, Wang X, Yang T, et al. High interleukin-8 levels associated with decreased survival in patients with cirrhosis following transjugular intrahepatic portosystemic shunt. Front Med 2022;9:829245.
[45] Ahmed Z, Badal J, Gangwani MK, et al. Sarcopenia is a risk factor for post-transjugular intrahepatic portosystemic shunt hepatic encephalopathy and mortality: a systematic review and meta-analysis. Indian J Gastroenterol 2024;43:748–759.
[46] Lattanzi B, D'Ambrosio D, Merli M. Hepatic encephalopathy and sarcopenia: two faces of the same metabolic alteration. J Clin Exp Hepatol 2019;9:125–130.
[47] Davuluri G, Allawy A, Thapaliya S, et al. Hyperammonaemia-induced skeletal muscle mitochondrial dysfunction results in cataplerosis and oxidative stress. J Physiol 2016;594:7341–7360.
[48] Qiu J, Thapaliya S, Runkana A, et al. Hyperammonemia in cirrhosis induces transcriptional regulation of myostatin by an NF-κB–mediated mechanism. Proc Natl Acad Sci USA 2013;110:18162–18167.
[49] Bureau C, Thabut D, Jezequel C, et al. The use of rifaximin in the prevention of overt hepatic encephalopathy after transjugular intrahepatic portosystemic shunt: a randomized controlled trial. Ann Intern Med 2021;174:633–640.
[50] Liang A, Brar S, Almaghrabi M, et al. Primary prevention of hepatic encephalopathy post-TIPS: a systematic review and meta-analysis. Medicine 2023;102:e35266.
[51] Bureau C, Larrue H, Cortes-Cerisuleo M, et al. EASL Clinical Practice Guidelines on TIPS. J Hepatol 2025;83:177–210.

Keywords: Transjugular intrahepatic portosystemic shunt (TIPS); Cirrhosis; Portal hypertension; Sarcopenia; Hepatic encephalopathy; Mortality after TIPS.

Received 7 August 2025; received in revised form 18 November 2025; accepted 24 November 2025; Available online 29 November 2025

Journal of Hepatology, Volume *8*

Supplemental information

Outcomes after TIPS in patients with cirrhosis and sarcopenia: A systematic review and meta-analysis

Maria de Brito Nunes, Maria Gabriela Delgado, Jaume Bosch, and Annalisa Berzigotti

Outcomes after TIPS in patients with cirrhosis and sarcopenia: A systematic review and meta-analysis

Maria de Brito Nunes, Maria Gabriela Delgado, Jaume Bosch, Annalisa Berzigotti

Table of contents

Table S1……………………………………………………………………………………2
Table S2……………………………………………………………………………………6
Table S3…………………………………………………………………………………....12
Table S3……………………………………………………………………………………13
Fig. S1………………………………………………………………………………………22
Fig. S2………………………………………………………………………………………23
Fig. S3………………………………………………………………………………………24
Fig. S4………………………………………………………………………………………25
Fig. S5………………………………………………………………………………………26
Fig. S6………………………………………………………………………………………27
Fig. S7………………………………………………………………………………………28
Fig. S8………………………………………………………………………………………29
Fig. S9………………………………………………………………………………………29
Fig. S10…………………………………………………………………………………....30

Table S1- Search strategy

	**Free Text**	**MeSH (controlled vocabulary)**	**Search**
**P**: patients with cirrhosis and **sarcopenia**	sarcop*.ti,ab,kw.	exp Sarcopenia/	Search 5
**I:** Transjugular Intrahepatic Portosystemic Shunt (TIPS)	(“Transjugular Intrahepatic Portosystemic Shunt” OR “tips” OR “tipss”).ti,ab,kw	exp Portasystemic Shunt, Transjugular Intrahepatic/	Search 10
**O:** Sarcopenia Reversal defined by L3-SMI or L3-PMI improvement, *de novo* Hepatic Encephalopathy, and overall Mortality post-TIPS			

Key articles identified in google scholar:

Nardelli, S., Lattanzi, B., Torrisi, S., Greco, F., Farcomeni, A., Gioia, S., ... & Riggio, O. (2017). Sarcopenia is risk factor for development of hepatic encephalopathy after transjugular intrahepatic portosystemic shunt placement. *Clinical Gastroenterology and Hepatology*, *15*(6), 934-936.

Liu, J., Ma, J., Yang, C., Chen, M., Shi, Q., Zhou, C., ... & Xiong, B. (2022). Sarcopenia in patients with cirrhosis after transjugular intrahepatic portosystemic shunt placement. *Radiology*, *303*(3), 711-719.

Li, T., Liu, J., Zhao, J., Bai, Y., Huang, S., Yang, C., ... & Xiong, B. (2023). Sarcopenia defined by psoas muscle thickness predicts mortality after transjugular intrahepatic portosystemic shunt. *Digestive Diseases and Sciences*, *68*(4), 1641-1652.

Tsien, C., Shah, S. N., McCullough, A. J., & Dasarathy, S. (2013). Reversal of sarcopenia predicts survival after a transjugular intrahepatic portosystemic stent. *European journal of gastroenterology & hepatology*, *25*(1), 85-93.

Benmassaoud, A., Roccarina, D., Arico, F., Leandro, G., Yu, B., Cheng, F., ... & Tsochatzis, E. (2020). Sarcopenia does not worsen survival in patients with cirrhosis undergoing transjugular intrahepatic portosystemic shunt for refractory ascites. *Official journal of the American College of Gastroenterology| ACG*, *115*(11), 1911-1914.

Search Strategy

WITHOUT FILTERS TO ENGLISH

Ovid MEDLINE(R) ALL <1946 to January 31, 2025>

1 exp Sarcopenia/ 9673

2 sarcop*.ti,ab,kw. 42847

3 1 or 2 43606

4 exp Portasystemic Shunt, Transjugular Intrahepatic/ 3322

5 ("Transjugular Intrahepatic Portosystemic Shunt" or "tips" or "tipss").ti,ab,kw. 39694

6 4 or 5 40426

7 3 and 6 82

(exp Sarcopenia/ or sarcop*.ti,ab,kw.) and (exp Portasystemic Shunt, Transjugular Intrahepatic/ or ("Transjugular Intrahepatic Portosystemic Shunt" or "tips" or "tipss").ti,ab,kw.)

Ovid MEDLINE(R) ALL <1946 to January 31, 2025>

1 exp Sarcopenia/ 9617

2 limit 1 to english language 9208

3 sarcop*.ti,ab,kw. 42688

4 2 or 3 41304

5 exp Portasystemic Shunt, Transjugular Intrahepatic/ 3316

6 ("Transjugular Intrahepatic Portosystemic Shunt" or "tips" or "tipss").ti,ab,kw. 39594

7 5 or 6 38577

8 4 and 7 81

Found: 81 articles

PUBMED RESEARCH

QUERY SEARCH 04.02.2025

("Sarcopenia"[MeSH Terms] OR "sarcop*"[Title/Abstract]) AND (Portasystemic Shunt [MeSH Terms] OR Transjugular Intrahepatic[MeSH Terms] OR "Transjugular Intrahepatic Portosystemic Shunt"[Title/Abstract] OR "tips"[Title/Abstract] OR "tipss"[Title/Abstract])

86 results

EMBASE (VIA OVIDSP)

Search Query

('Sarcopenia'/ or sarcop*.ti,ab,kw.) AND ('Portasystemic Shunt, Transjugular Intrahepatic'/ or "Transjugular Intrahepatic Portosystemic Shunt".ti,ab,kw or "tips".ti,ab,kw or "tipss".ti,ab,kw.)

Embase <1974 to January 31, 2025>

('Sarcopenia'/ or sarcop*.ti,ab,kw.) and ('Portasystemic Shunt, Transjugular Intrahepatic'/ or "Transjugular Intrahepatic Portosystemic Shunt".ti,ab,kw. or "tips".ti,ab,kw. or "tipss".ti,ab,kw.) 179

Or

Embase <1974 to January 31, 2025>

1 ('Sarcopenia'/ or sarcop*.ti,ab,kw.) and ('Portasystemic Shunt, Transjugular Intrahepatic'/ or "Transjugular Intrahepatic Portosystemic Shunt".ti,ab,kw. or "tips".ti,ab,kw. or "tipss".ti,ab,kw.) 179

2 'Sarcopenia'/ 21234

3 sarcop*.ti,ab,kw. 55021

4 2 or 3 57907

5 'Portasystemic Shunt, Transjugular Intrahepatic'/ 5912

6 "Transjugular Intrahepatic Portosystemic Shunt".ti,ab,kw. 4779

7 "tips".ti,ab,kw. 47698

8 "tipss".ti,ab,kw. 680

9 6 or 7 or 8 49413

10 4 and 9 143

COCHRANE LIBRARY:

Search 04.02.2025

Trials and reviews

"Sarcopenia" OR sarcop* AND "Portasystemic Shunt, Transjugular Intrahepatic" OR "Transjugular Intrahepatic Portosystemic Shunt" OR tips OR tips

clinicaltrials.gov

search: 03.02.2025

condition/Disease: Sarcopenia in Liver Cirrhosis

intervention/treatment: Transjugular intrahepatic portosystemic shunt \(TIPS\)

NO FILTERS USED

all studies, 3 studies found

EU clinical Trials

0 STUDIES FOUND, No filters used

Table S2- Risk bias assessment considering the selected studies (Newcastle-Ottawa Scale for cohort studies)

	**Representativeness of the exposed cohort**	**Selection of the non-exposed cohort**	**Ascertainment of exposure**	**Demonstration that outcome of interest was not present at start of study**	**Comparability**	**Assessment of outcome**	**Was follow-up long enough for outcomes to occur**	**Adequacy of follow-up of cohorts**	**TOTAL SCORE**
The Added Value of Sarcopenia on Existing Risk Scores to Predict Mortality after TIPS Placement: A Multicenter Study.	*	No applicable (NA)	*	*	*	*	*	*	7
Transjugular intrahepatic portosystemic shunt insertion improves muscle mass but not muscle function or frailty measures.	*	NA	*	*	*	*	*	*	7

Improvement of sarcopenia is beneficial for prognosis in cirrhotic patients after TIPS placement.	*	*	*	*	**	*	*	*	9
Post-transjugular Intrahepatic Portosystemic Shunt HepaticEncephalopathy: Sarcopenia Adds Insult to Injury	*	NA	*	*	**	*	*		7
Predictors of Improvement of Sarcopenia after Transjugular Intrahepatic Portosystemic Shunt Creation in Cirrhotic Patients		NA	*	*	*	*	*	*	6
TIPS placement as the first-line therapy to prevent variceal rebleeding in patients with cirrhosis and sarcopenia.	*	*	*	*	**	*	*		8
Inclusion of sarcopenia improves the prognostic value	*	*	*	*	**	*	*	*	9

of MELD score in patients after transjugular intrahepatic portosystemic shunt									
Sarcopenia in Patients with Cirrhosis after Transjugular Intrahepatic Portosystemic Shunt Placement.	-	NA	*	*	*	*	*	*	6
The improvement in body composition including subcutaneous and visceral fat reduces ammonia and hepatic encephalopathy after transjugular intrahepatic portosystemic shunt	-	NA	*	*	*	*	*	*	7
Sarcopenia Does Not Worsen Survival in Patients With Cirrhosis Undergoing Transjugular Intrahepatic Portosystemic Shunt	*	NA	*	*	*	*	*	*	7

for Refractory Ascites.									
Study 11 The modification of quantity and quality of muscle mass improves the cognitive impairment after TIPS.	*	NA	*	*	*	*	*	*	7
Sarcopenia Is Risk Factor for Development of Hepatic Encephalopathy After Transjugular Intrahepatic Portosystemic Shunt Placement.	*	NA	*	*	*	*	*	*	7
Increase of radiologically determined muscle area in patients with liver cirrhosis after transjugular intrahepatic portosystemic shun	*	NA	*	*	*	*	*	-	6
Sarcopenia Defined by Psoas Muscle Thickness Predicts Mortality After TIPS	*	NA	*	*	*	*	*	-	6
	-	NA	-	*	-	*	*	*	4

Effects of transjugular intrahepatic portosystemic shunt on abdominal muscle mass in patients with decompensated cirrhosis									
Development and Validation of Prognostic Models to Estimate the Risk of Overt Hepatic Encephalopathy After TIPS Creation: A Multicenter Study	*	NA	*	-	*	*	*	*	6
Low adipose tissue index as an indicator of hepatic encephalopathy in cirrhotic patients following transjugular intrahepatic portosystemic shunt.	*	NA	*	-	*	*	*	*	6
Sarcopenia is an independent risk factor for short-term mortality in patients undergoing transjugular	*	*	*	*	*	*	*	*	8

intrahepatic portosystemic shunt									
Body Compositions Correlate With Overt Hepatic Encephalopathy after Transjugular Intrahepatic Portosystemic Shunt	-	*	*	*	*	*	*	*	**7**
Validating the prognostic value of muscle changes in patients with cirrhosis undergoing transjugular intrahepatic portosystemic shunt	-	*	*	*	*	*	*	*	**7**

Table S3- GRADE Summary of Findings

Outcome	No. of Studies (Patients)	Effect Estimate (95% CI)	Certainty of Evidence	Main Limitations
**Sarcopenia improvement after TIPS**	10 studies (1,008 patients; 596 with sarcopenia)	-57% improvement (95% CI: 48–65%)<br>-Mean SMI ↑ 4.53 cm²/m²	●●○○<br>Low	Observational design; substantial heterogeneity (I^2 = 68.5%); limited subgroup analyses
**Overt hepatic encephalopathy after TIPS**	11 studies (1,839 patients; 1,080 with sarcopenia)	-Sarcopenia vs. no sarcopenia: OR = 3.40 (95% CI: 1.85–6.25), $p < 0.001$<br>-Incidence: 43% vs. 12%	●●○○<br>Low	Observational studies; high heterogeneity (I^2 = 85.7%); visual funnel asymmetry (not confirmed)
**Mortality after TIPS**	8 studies (1,194 patients; 211 deaths)<br>5 with HRs	-HR = 1.95 (95% CI: 0.89–4.31), p = 0.078<br>-Per unit ↑ SMI: HR = 0.97 (95% CI: 0.88–1.06), p = 0.34	●●○○<br>Low	Wide CIs; non-significant results; substantial heterogeneity (I^2 = 71.7%); limited stratified data

Note: The certainty of evidence was assessed using the GRADE approach. All outcomes start at low certainty due to observational study designs. Further downgrades were applied for inconsistency and imprecision where applicable.

Table S4 – Baseline characteristics of the included studies

Study	Title	Authors	Year of publication	Design	Sample size	Population characteristics	Indication of TIPS	Duration of follow-up	Main Outcome
1	The Added Value of Sarcopenia on Existing Risk Scores to Predict Mortality after TIPS Placement: A Multicenter Study.	Xiong, B. et al	2023	Retrospective	386 derivation cohort 198 validation cohort	Derivative cohort gender: 249 (64.5%) male Etiology: 228 (59.1) Hepatitic B CHILD 7.5 MELD score 11.5<br><br>validation cohort gender: 120 (60.6%) male Etiology: 61 (52.1%) Hepatitic B CHILD 7.28 MELD score 11.3	Derivative cohort: variceal bleeding: 349 refractory ascites: 37	24 months (1,3,6, 12 months after TIPS Then annually until OLT, death or end of the study)	FIPS score was highly correlated with the severity of sarcopenia and sarcopenia reversal after TIPS.<br><br>Sarcopenia could improve the prognostic ability of existing scores
2	Transjugular intrahepatic portosystemic shunt insertion improves muscle mass but not muscle function or frailty measures.	Hey, P. et al	2023	Prospective	12	Gender: 8 (67%) male Etiologie: 50% ArLD, 25% MASLD MELD score 16 ± 5	Refractory Ascites: 11 patients recurrent variceal bleeding: 1 patient	6 months	Muscle mass increased following TIPS insertion
3	Improvement of sarcopenia is beneficial for prognosis in cirrhotic patients after TIPS placement.	Liu, J. et al	2023	Retrospective	109	Gender: male 79 (72.5%) Etiology: HBV 71 (65%) CHILD 7.0 (6.0 - 8.0)	Varicela bleeding:103 (94.5%) refractory ascites: 6 (5.5%)	60 Months (1 month, 3 months, 6 months and every 1 year thereafter)	Reversal of sarcopenia or significant SMI improvement by TIPS was associated with a reduced risk

						MELD score: 11.4 ± 3.6			of death and overt HE.
4	Post-transjugular Intrahepatic Portosystemic Shunt HepaticEncephalopathy: Sarcopenia Adds Insult to Injury	Kapoor, P. et al	2023	Retrospective	79	Gender: male 68 (86%)<br>Etiology: ArLD (56%), MASH 16 (20%)<br>CHILD A/B/C: 3 (4%) / 56 (71%) / 20 (25%)<br>MELD score 15.75 ± 6.38	refractory ascites 56 (71%)<br>Variceal bleed 23 (29%)	± 9 months	Increased skeletal muscle index post-TIPS is associated with decreased risk of hepatic encephalopathy
5	TIPS placement as the first-line therapy to prevent variceal rebleeding in patients with cirrhosis and sarcopenia.	Xiong, B. et al	2023	Retrospective	262	Total<br>gender: 173 (66%) male<br>Etiology: Hepatitis B:157 (59.9%)<br>CHILD 7.4 (1.5)<br>MELD 11.2 (3.4)<br>MELD Na score 12.2 (4.3)<br><br>Non-sarcopenia<br>gender: 94 (57.7%) male<br>Etiology: Hepatitis B: 93 (57.1%)<br>CHILD 7.2 (1.4)<br>MELD 10.7 (2.8)<br>MELD Na score 11.8 (4.0)	Prevention of rebleeding	36 Months (1, 3, 6, 12 months after TIPS. Then annually until OLT, death or end of the study)	Baseline sarcopenia did not increase the risk of post-TIPS mortality and overt HE

						Sarcopenia gender: 79 (79.8%) male<br>Etiology: Hepatitis B: 64 (64.6%)<br>CHILD 7.8 (1.6)<br>MELD 12 (3.9)<br>MELD Na score 12.9 (4.6)			
6	Increase of radiologically determined muscle area in patients with liver cirrhosis after transjugular intrahepatic portosystemic shunt	March, C. et al	2023	Retrospective	52	Gender: 42 (80.8%) male<br>Etiology: ArLD (69.2%), MASH<br>CHILD A/B/C: 3 (7.7%)/26 (66.7%)/ 10 (25.6%)<br>MELD score 12.8 ± 4.6<br>Sarcopenia Pre-TIPS: 84.6% men, 92.3% women	Refractory ascites: 39 (75%), variceal bleeding: 11 (21.1%)	median follow-up of 16.5 months (6-93)	TIPS procedure has a positive impact on muscle area in patients with liver cirrhosis and sarcopenia. Higher albumin levels were observed in patients with increased SMI after TIPS compared to patients without an increase
7	Low adipose tissue index as an indicator of hepatic encephalopathy in cirrhotic patients following transjugular	Wang, C. et al	2023	Retrospective	191	Gender: 116 male<br>Etiology: HBV 79 (41.4%), Schistosomiasis 42 (22%)<br>CHILD 7.4 ± 1.7<br>MELD score 10.7 ±	variceal bleeding (179, 93.7%)	median follow-up time was 28 months	Body composition indexes (VFAI, SFAI, SMI) could be utilized to predict the HE

						3.4<br>MELD-Na score 11.3 ± 4.2<br>Sarcopenia Pre-TIPS: 73 (38.2%) patients			risk of cirrhotic patients after TIPS
8	Sarcopenia Defined by Psoas Muscle Thickness Predicts Mortality After TIPS	Li, T. et al	2023	Retrospective	249	Gender: 162 (65.1%) male<br>Etiology: HBV 150 (60.2%)<br>CHILD: 7.5 ± 1.58<br>MELD score 11.78 ± 1.58 (A: 62/B 164/ C 23 patients)<br>Meld-Na score: 12.81 ± 5.0<br>Sarcopenia Pre-TIPS 82 (32.9%) patients	variceal bleeding (219, 88%), refractory ascites (3, 12%)	mean follow-up time was 22.4 ± 15.9 months (1, 6, and 12 months, every 12 months)	Overall survival was significantly lower in the sarcopenia group
9	Predictors of Improvement of Sarcopenia after Transjugular Intrahepatic Portosystemic Shunt Creation in Cirrhotic Patients	Huang, S. et al	2022	Retrospective	111	Gender: 81 (73%) male<br>Etiology: HBV 71 (64%)<br>CHILD 7.5 ± 1.5<br>MELD score 11.4 ± 3.6	Variceal bleeding: 105 patients refractory ascites: 6 patients	6 months	Pre-TIPS SMI and changes in portal pressure gradient were found to be independent risk factor for experiencing substantial improvement in post-TIPS SMI.

10	Inclusion of sarcopenia improves the prognostic value of MELD score in patients after transjugular intrahepatic portosystemic shunt	Bai, Y-W. et al	2022	Retrospective	289	Total<br>Gender: 191 (66.1%) male<br>Etiology: Hepatitis B:187 (64.7%)<br>CHILD 7.6 ± 1.8<br>MELD 11.9 ± 4.0<br>MELD Na score 12.8 ± 5.1<br><br>Non-sarcopenia<br>Gender: 93 (61.6%) male<br>Etiology: Hepatitis B: 97 (64.2%)<br>CHILD 7.3±1.8<br>MELD 11.3 ± 3.4<br>MELD Na score 12.1 ± 4.5<br><br>Sarcopenia<br>Gender: 98 (71%) male<br>Etiology: Hepatitis B: 90 (65.2%)<br>CHILD 7.9 ± 1.8<br>MELD 12.6 ± 4.4<br>MELD Na score 13.7 ± 5.5	Variceal bleeding: 256 (88%), refractory ascites 33 (11.4%)	24 months	Sarcopenia is independently correlated with post-TIPS mortality.<br><br>MELD-Sarcopenia score showed the best performance in predicting post-TIPS mortality than the traditional predictive models

11	Sarcopenia in Patients with Cirrhosis after Transjugular Intrahepatic Portosystemic Shunt Placement.	Liu, J. et al	2022	Retrospective	224	Total gender: 159 male Etiology: Hepatitis B:153 (68%) CHILD 7.7 ± 1.6 MELD 11.9 ± 3.9 MELD Na score 12.9 ± 4.8 Sarcopenia Pre-TIPS: 145 patients	Variceal bleeding: 193 (86.2%), refractory ascites 31 (13.8%)	12 Months (2, 5 and 12 months after TIPS)	In cirrhotic patients with sarcopenia, skeletal muscle, and fat mass increased after TIPS placement.<br>The reversal of sarcopenia could reduce the mortality
12	Development and Validation of Prognostic Models to Estimate the Risk of Overt Hepatic Encephalopathy After TIPS Creation: A Multicenter Study	Yang, C. et al	2023	Retrospective	Derivation cohort: 276 Validation cohort 182	Derivation cohorte: Gender: 181 (65.6%) male Etiology. Viral 187 (67.8%) CHILD 7.68 (1.67) MELD score 11.8 (3.86) MELD-Na score 12.6 (4.77) Sarcopenia Pre-TIPS: 170 (61.6%) patients	variceal bleeding (248, 89.9%), refractory ascites (28, 10.1%)	3 months	Sarcopenia was associated with an increased risk of post-TIPS overt HE
13	The improvement in body composition including subcutaneous and visceral fat reduces ammonia and hepatic encephalopathy after transjugular	Gioia, S. et al	2021	Retrospective	35	Gender: 28 male etiology:Alcohol 16 patients, viral 12 patients CHILD (A/B/C): 17/16/2 MELD score 11.9 ± 4	variceal bleeding (16 patients), refractory ascites (19 patients)	mean follow-up 19 ± 15 months (1,3, 6 month and every 6 months)	Overt HE was significantly lower in the patients with improved SMI and in

	intrahepatic portosystemic shunt					sarcopenia Pre-TIPS 19 patients			the patients with improved subcutaneous adipose tissue
14	Sarcopenia Does Not Worsen Survival in Patients With Cirrhosis Undergoing Transjugular Intrahepatic Portosystemic Shunt for Refractory Ascites.	Benmassaoud, A. et al	2020	Retrospective	107	Total<br>Gender: 65 (60.71%) male<br>Etiology: ArLD: 69 (64.5%)<br>CHILD 8 (2) /B: 96, C:11<br>MELD Na score 11 (8)<br><br>Non-sarcopenia<br>Gender: 20 (43.5%) male<br>Etiology: ArLD: 26 (56.5.2%)<br>CHILD 8 (2)<br>MELD Na score 12 (6)<br><br>Sarcopenia<br>Gender: 45 (73.8%) male<br>Etiology: ArLD: 43 (70.5%)<br>CHILD 8 (2)<br>MELD Na score 11 (8)	refractory ascites	5 years	Sarcopenia<br><br>is not associated with de novo HE or increased mortality post-TIPS

15	The modification of quantity and quality of muscle mass improves the cognitive impairment after TIPS.	Gioia, S. et al	2019	Retrospective	27	Gender: 23 male<br>Etiology: Viral 12, ArLD 8<br>CHILD 7.1 ± 13<br>MELD score 11.3 ± 4.2<br>Sarcopenia Pre-TIPS: 21 (78%) patients	refractory ascites (15 patients), variceal bleeding (12 patients)	8.3 months (range 5.8-19)	Overt HE was significantly lower in the patients with the improvement of sarcopenia during the follow-up
16	Sarcopenia Is Risk Factor for Development of Hepatic Encephalopathy After Transjugular Intrahepatic Portosystemic Shunt Placement.	Nardelli, S. et al	2017	Prospective	46	Gender: 34 male<br>Etiology: ArLD, viral<br>CHILD 7.6 ± 1.5<br>MELD score 11.3 ± 3.3<br>Sarcopenia Pre-TIPS: 26 (57%) patients	variceal bleeding, refractory ascites (equal)	7 ± 9 months	MELD and sarcopenia were associated independently with the development of HE after TIPS placement
17	Sarcopenia is an independent risk factor for short term mortality in patients undergoing transjugular intrahepatic portosystemic shunt	Stoffel, E. et al	2024	Retrospective	232	Gender: 105 male<br>Etiology: hepatitis C, ArLD, MASH<br>Sarcopenia Pre-TIPS: 136 patients<br>MELD score12.82 ± 4.08	variceal bleeding (29.74%), refractory ascites (47.84%)	12 months or until death	Sarcopenia was an independent risk factor for 1-year mortality in patients undergoing TIPS
18	Body Compositions Correlate With Overt Hepatic Encephalopathy after Transjugular	Shi, W. et al	2024	Retrospective	571	Gender: 439 male<br>Etiology: hepatitis, ArLD, cholestatic	variceal bleeding and ascites	12 months	SATI and myosteatosis are independent risk factors for

	Intrahepatic Portosystemic Shunt								post-TIPS overt HE
19	Validating the prognostic value of muscle changes in patients with cirrhosis undergoing transjugular intrahepatic portosystemic shunt	Wen, Z. et al	2024	Retrospective	202	Gender:109 male<br>Etiology: viral hepatitis, ArLD, autoimmune<br>MELD: 10.0 (8.0-12.0)<br>CHILD A 36.6%, CHILD B 58.4%, CHILD C 5%	Variceal bleeding (99%)	Median of 33.6 months	The presence of muscle changes before TIPS significantly increased the risk of post-TIPS mortality but did not increase the risk of decompensation events
20	Effects of Transjugular intrahepaticpostosystemic shunt on abdominal muscle mass in patients with decompensated cirrhosis	Wu et al	2023	Retrospective	25	Gender: 19 male<br>Etiology: viral hepatitis, alcohol liver disease<br>MELD 11<br>CHILD A 32%, CHILD B 56%, CHILD C 12%	Variceal bleeding and refractory ascites and hydrothorax	6 and 12 months	The combination of sarcopenia and reduced BMI were prognostic factors for reduced overall survival in multivariate analysis

TIPS: transjugular intrahepatic portosystemic shunt. ArLD: Alcohol related liver disease. MASLD: metabolic dysfunction associated steatotic liver disease. VFAI: visceral fat area index subcutaneous fat area index. SFAI: subcutaneous fat area index, SMI: skeletal muscle index, SATI: subcutaneous adipose tissue

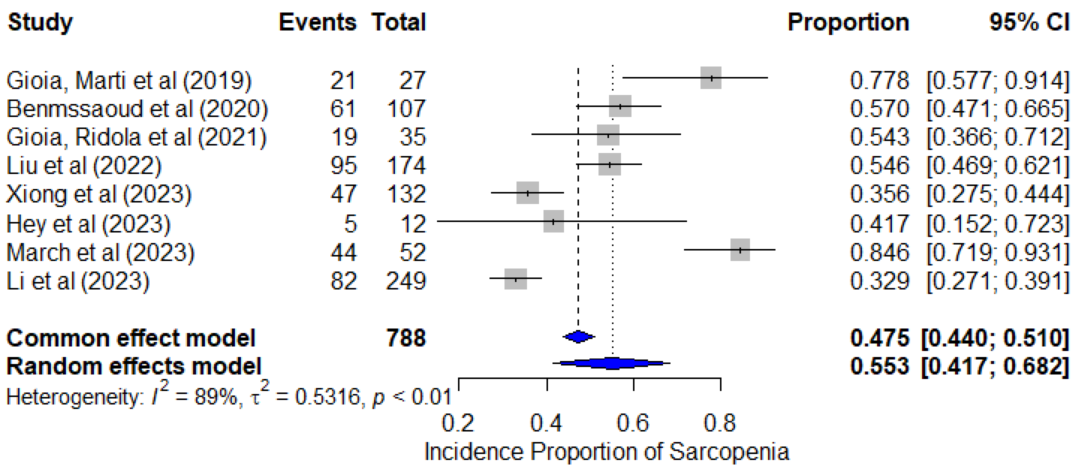

Fig. S1- Forest plot illustrating the pooled prevalence of sarcopenia across the studies. Proportions with 95% confidence intervals (CIs) are shown for each study.

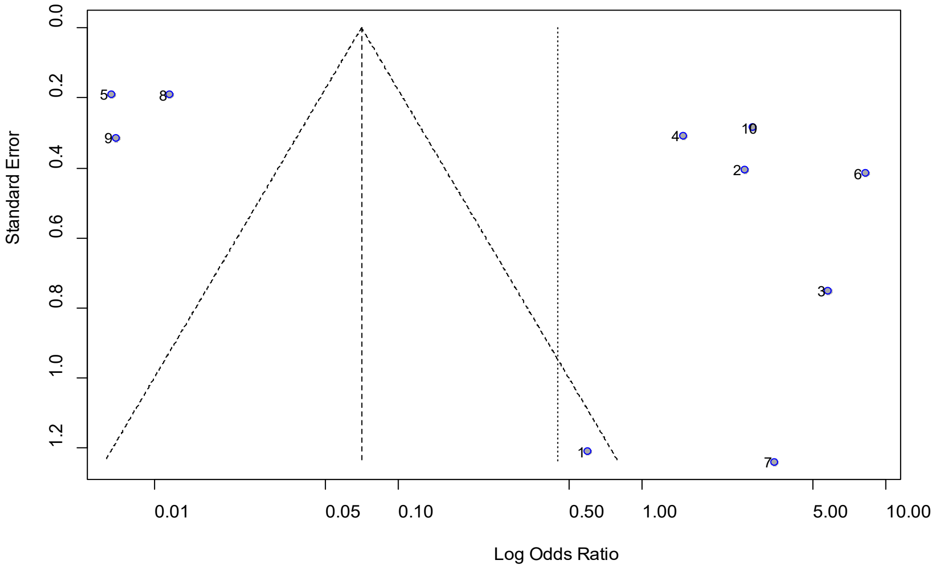

Fig. S2 - Funnel plot assessing potential publication bias among studies evaluating the proportion of patients demonstrating improvement in sarcopenia after TIPS. Asymmetry in the distribution of studies may suggest the presence of publication bias or small-study effects.

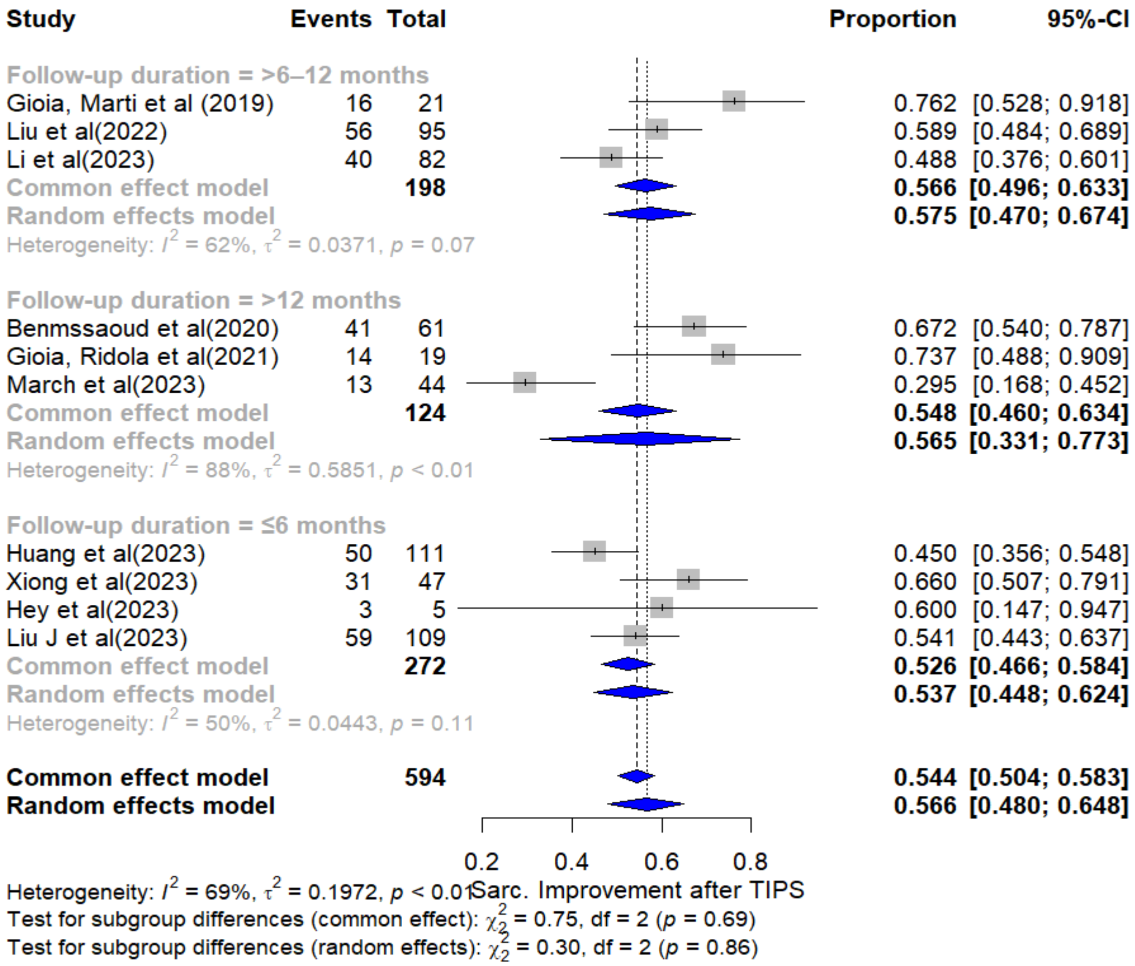

Fig. S3-Forest plot showing a pooled and subgroup analysis of the proportion of sarcopenia improvement after TIPS, with results stratified by follow-up duration. Proportions with 95% confidence intervals (CIs) are shown for each study.

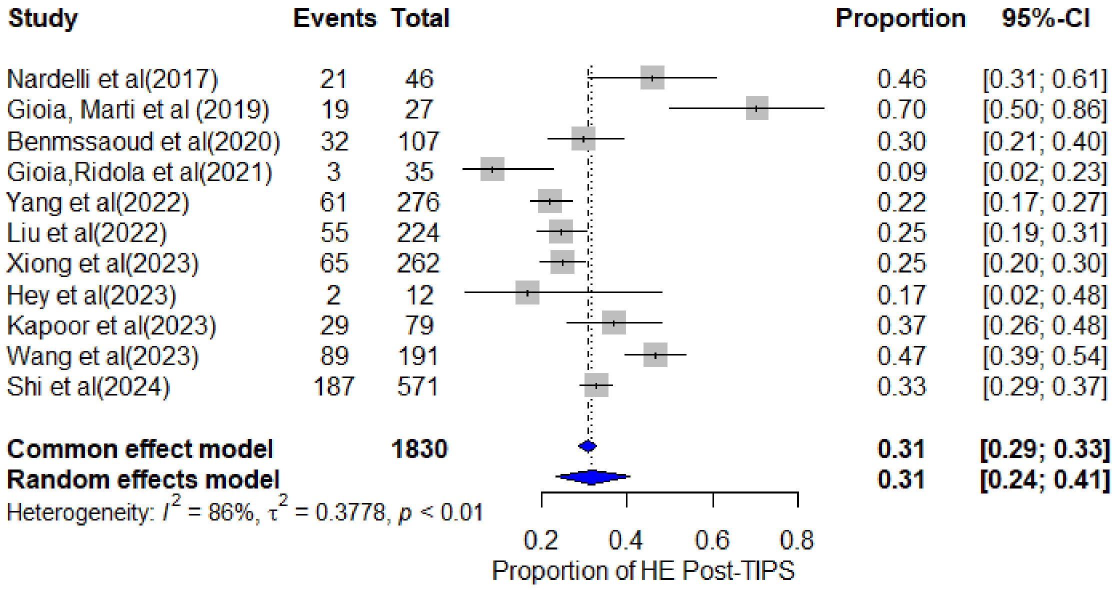

Fig. S4-Forest plot showing the proportion of hepatic encephalopathy after TIPS. Proportions with 95% confidence intervals (CIs) are shown for each study.

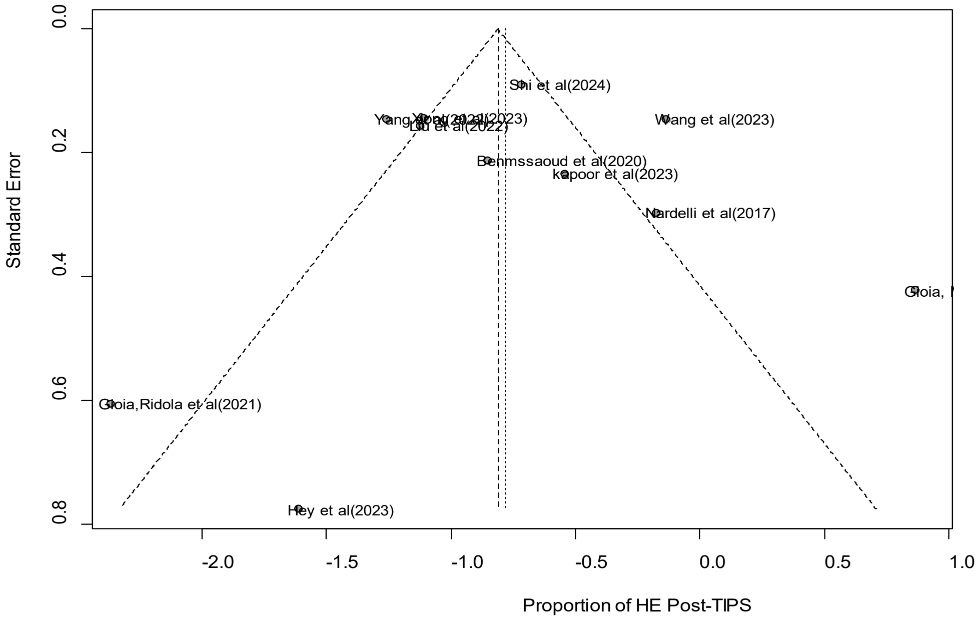

Fig. S5-Funnel plot assessing potential publication bias among studies evaluating the proportion of HE after TIPS in patients with and without sarcopenia. Asymmetry in the distribution of studies may suggest the presence of publication bias or small-study effects.

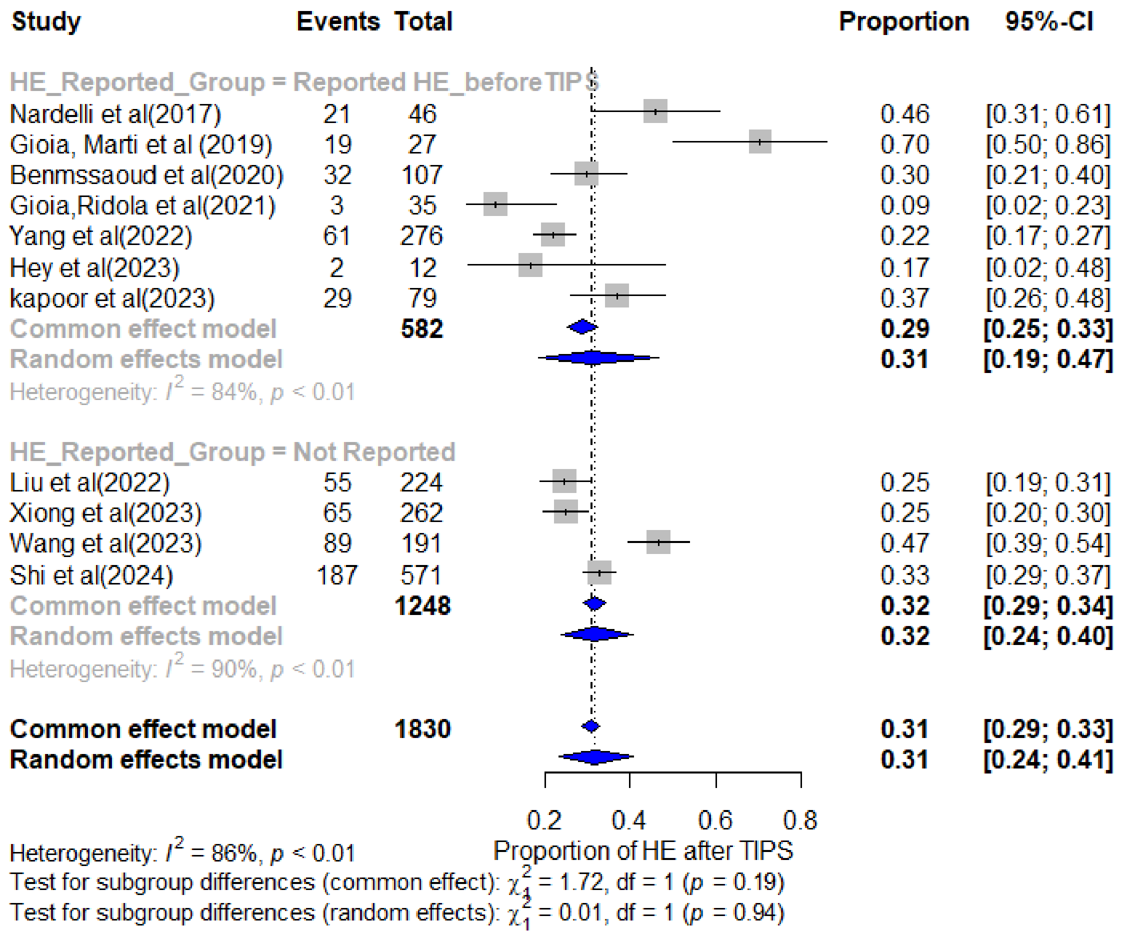

Fig. S6- Forest plot of subgroup analysis comparing studies reporting and studies not reporting presence of HE before TIPS. Proportions with 95% confidence intervals (CIs) are shown for each study.

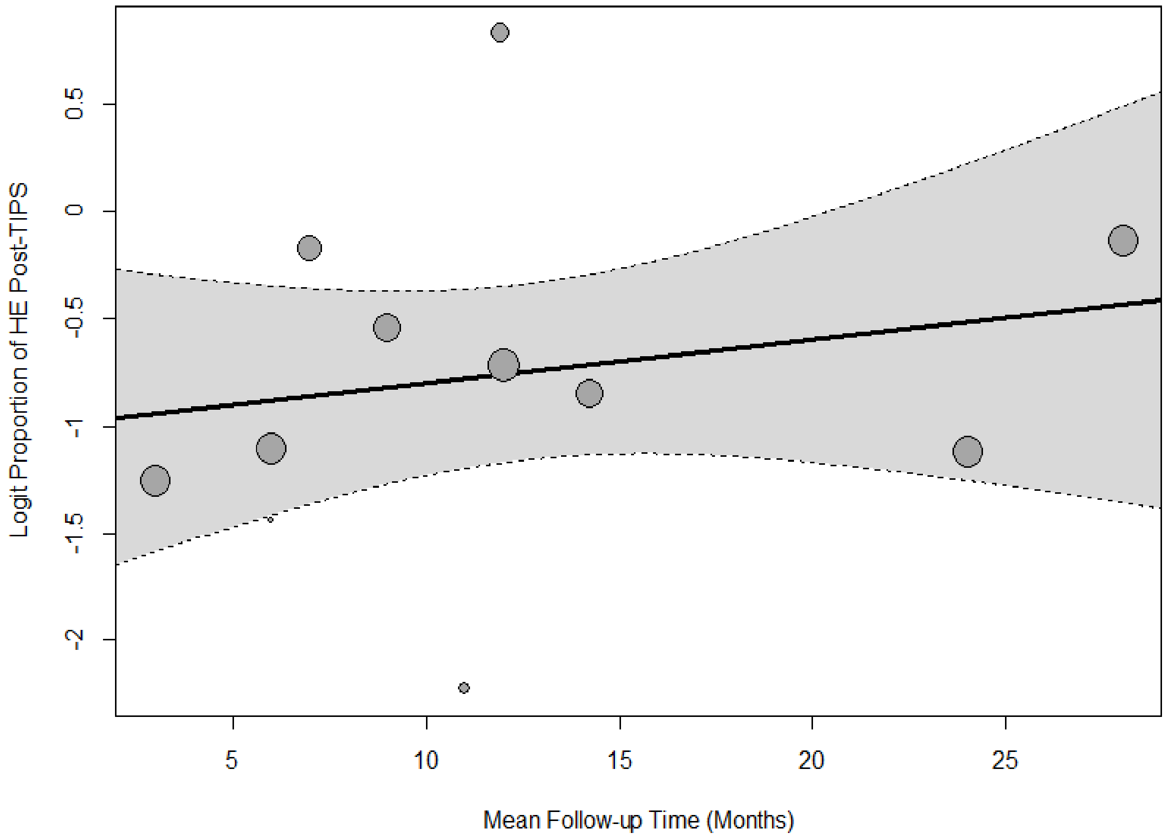

Fig. S7- Bubble plot showing a meta-regression analysis of the association between mean follow-up duration (months) and the logit-transformed proportion of hepatic encephalopathy (HE) events after TIPS. Each circle represents an individual study, with circle size proportional to the study weight in the analysis. The solid line represents the fitted regression line, and the shaded area indicates the 95% confidence interval.

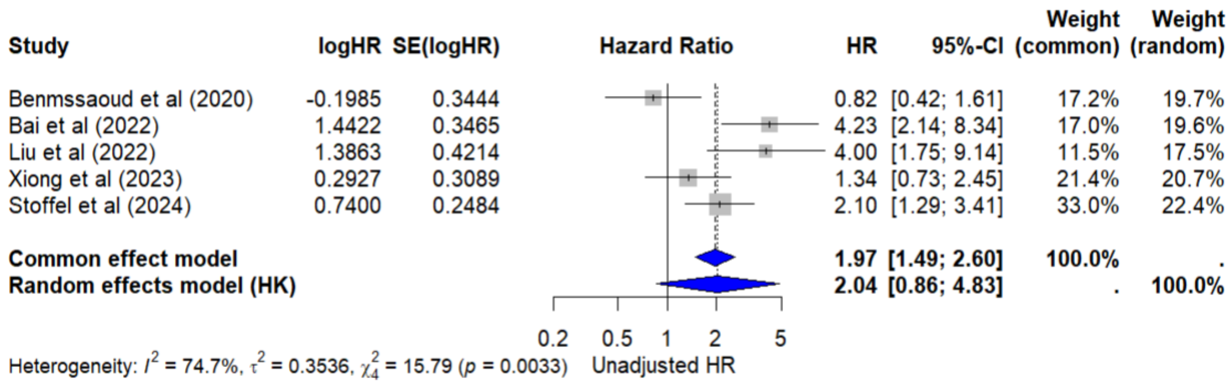

Fig. S8- Forest plot showing unadjusted Hazard Ratios (HR) for the association between sarcopenia and mortality after TIPS. Unadjusted HR was obtained in each study by univariate cox regression model. HR with 95% confidence intervals (CIs) are shown for each study.

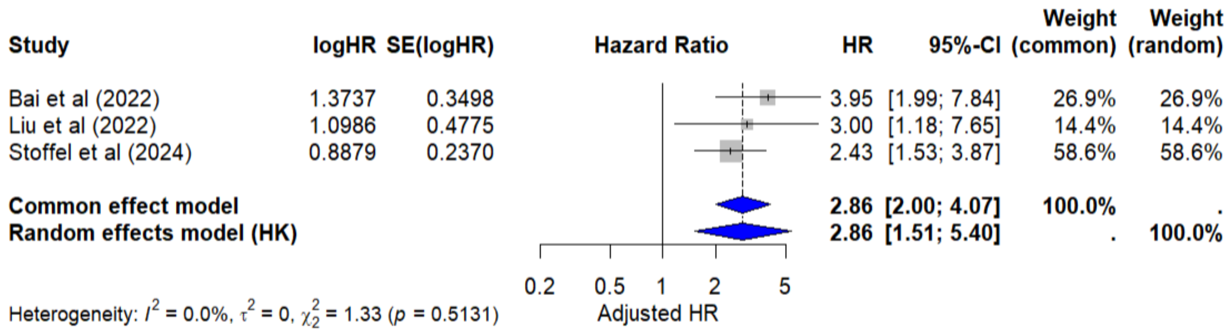

Fig. S9- Forest plot showing adjusted Hazard Ratios (HR) for the association between sarcopenia and mortality after TIPS. Adjusted HR was obtained in each study by multivariate cox regression model. HR with 95% confidence intervals (CIs) are shown for each study.

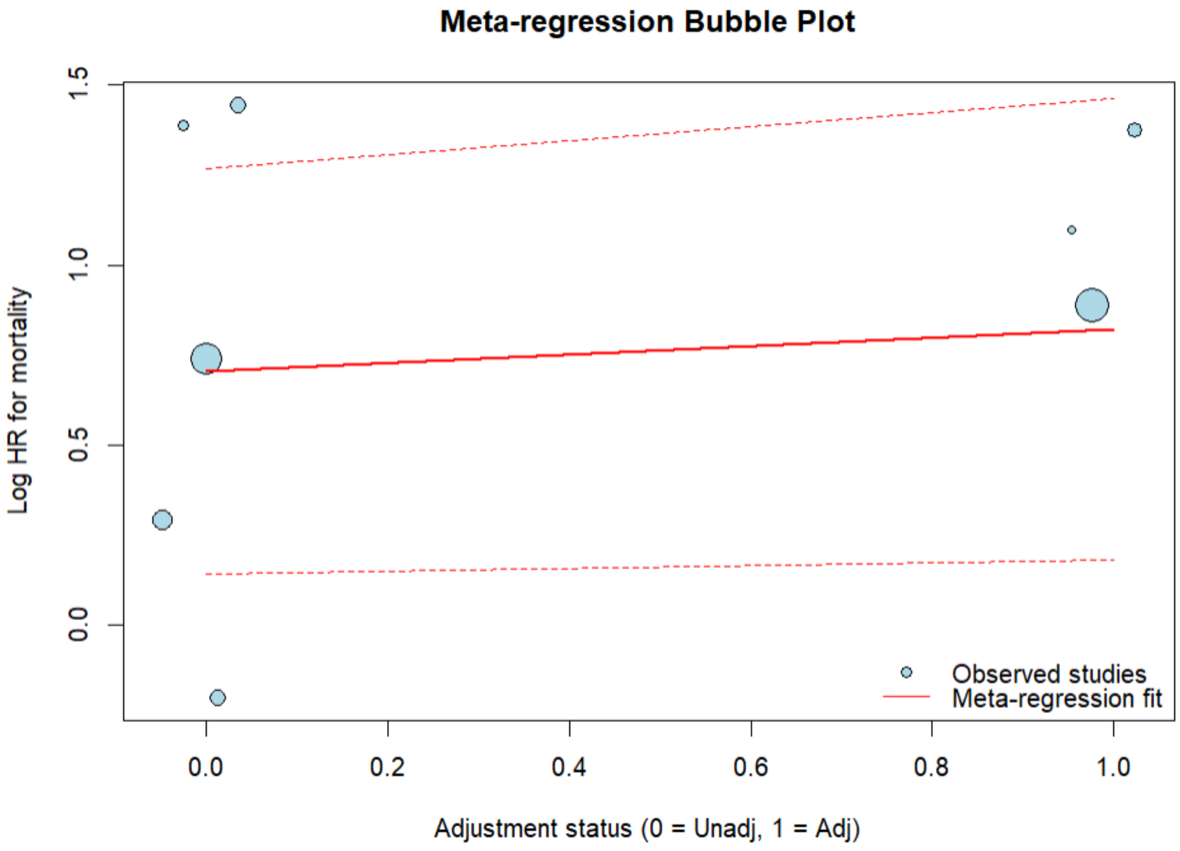

**Multivariate Meta-Analysis Model (k = 8; method: REML)**	logLik Deviance AIC BIC AICc<br>-4.5456 9.0912 15.0912 14.4665 27.0912
**Variance Components**	estim sqrt nlvls fixed factor

	sigma^2 0.3049 0.5521 5
**Test for Residual Heterogeneity**	QE(df = 6) = 17.1209, p-val = 0.0088
**Test of Moderators (coefficient 2)**	QM(df = 1) = 0.2161, p-val = 0.6420
**Model Results**	**estimate se zval pval ci.lb ci.ub**<br>**intrcpt 0.7051 0.2873 2.4546 0.0141 0.1421 1.2681 ***<br>**adjusted 0.1169 0.2515 0.4649 0.6420 -0.3760 0.6098**

Fig. S10- Meta-regression bubble plot showing hazard ratios (HRs) for mortality after TIPS according to adjustment status. Each bubble represents an individual study's log-transformed HR, with bubble size proportional to the study’s weight (inverse of variance). The horizontal axis indicates the adjustment status (0 = unadjusted; 1 = multivariate adjusted). The solid red line shows the fitted meta-regression slope, with dashed lines representing its 95% confidence interval. The regression line is almost horizontal, reflecting that multivariate adjustments did not modify the association between sarcopenia and mortality risk. This non-significant slope indicates that adjustment for confounders did not influence the reported HRs (β = 0.12; 95% CI: –0.38 to 0.61; p = 0.64).
